# *Crypton *transposons: identification of new diverse families and ancient domestication events

**DOI:** 10.1186/1759-8753-2-12

**Published:** 2011-10-19

**Authors:** Kenji K Kojima, Jerzy Jurka

**Affiliations:** 1 Genetic Information Research Institute, 1925 Landings Drive, Mountain View, CA 94043, USA

**Keywords:** tyrosine recombinase, *Crypton*, domestication, transposon, DUF3504

## Abstract

**Background:**

"Domestication" of transposable elements (TEs) led to evolutionary breakthroughs such as the origin of telomerase and the vertebrate adaptive immune system. These breakthroughs were accomplished by the adaptation of molecular functions essential for TEs, such as reverse transcription, DNA cutting and ligation or DNA binding. *Cryptons *represent a unique class of DNA transposons using tyrosine recombinase (YR) to cut and rejoin the recombining DNA molecules. *Cryptons *were originally identified in fungi and later in the sea anemone, sea urchin and insects.

**Results:**

Herein we report new *Cryptons *from animals, fungi, oomycetes and diatom, as well as widely conserved genes derived from ancient *Crypton *domestication events. Phylogenetic analysis based on the YR sequences supports four deep divisions of *Crypton *elements. We found that the domain of unknown function 3504 (DUF3504) in eukaryotes is derived from *Crypton *YR. DUF3504 is similar to YR but lacks most of the residues of the catalytic tetrad (R-H-R-Y). Genes containing the DUF3504 domain are potassium channel tetramerization domain containing 1 (*KCTD1*), *KIAA1958*, zinc finger MYM type 2 (*ZMYM2*), *ZMYM3*, *ZMYM4*, glutamine-rich protein 1 (*QRICH1*) and "without children" (*WOC*). The *DUF3504 *genes are highly conserved and are found in almost all jawed vertebrates. The sequence, domain structure, intron positions and synteny blocks support the view that *ZMYM2*, *ZMYM3*, *ZMYM4*, and possibly *QRICH1*, were derived from *WOC *through two rounds of genome duplication in early vertebrate evolution. *WOC *is observed widely among bilaterians. There could be four independent events of *Crypton *domestication, and one of them, generating *WOC*/*ZMYM*, predated the birth of bilaterian animals. This is the third-oldest domestication event known to date, following the domestication generating telomerase reverse transcriptase (*TERT*) and *Prp8*. Many *Crypton*-derived genes are transcriptional regulators with additional DNA-binding domains, and the acquisition of the DUF3504 domain could have added new regulatory pathways via protein-DNA or protein-protein interactions.

**Conclusions:**

*Cryptons *have contributed to animal evolution through domestication of their YR sequences. The DUF3504 domains are domesticated YRs of animal *Crypton *elements.

## Background

The structural and mechanistic variety of transposable elements (TEs) is well-documented [1]. They encode proteins that include diverse functional domains involved in catalysis or interaction with DNA, RNA and other proteins. Because of this diverse repertoire, TEs can supply functional modules to generate new genes. "Molecular domestication" of transposable elements [2] led to evolutionary milestones such as the origin of telomerase and the vertebrate adaptive immune system. Telomerase reverse transcriptase (TERT) provides a solution for end replication problems accompanying linear chromosome replication and was derived from a reverse transcriptase (RT) related to *Penelope*-like elements in the very early stage of eukaryote evolution [3,4]. V(D)J recombination is a mechanism used in jawed vertebrates to generate a variety of immunoglobulins and T-cell receptors. It is catalyzed by the recombination activating gene 1 (*RAG1*) derived from a transposase encoded by the *Transib *family of DNA transposons [5]. Different kinds of transposon proteins were domesticated, including transposase, integrase, RT, envelope and gag proteins [6]. Herein we report in-depth studies of another type of transposon enzyme, tyrosine recombinase (YR), which was repeatedly domesticated in the history of animals.

To date four types of enzymes are known to catalyze DNA integration of eukaryotic transposons: DDE-transposase, YR, rolling-circle replication initiator and the combination of RT and endonuclease (EN) [7]. DDE-transposase is the most abundant gene in nature [8] and is carried by many DNA transposon superfamilies, self-synthesizing transposons (*Polinton*), as well as long terminal repeat (LTR) retrotransposons (*Gypsy*, *Copia*, *BEL *and endogenous retroviruses) [1,9-11]. They share three conserved amino acids (DDD or DDE) at their catalytic sites, which are separated by amino acid sequences of varying length. Some domesticated DDE-transposases became DNA-binding proteins, such as CENP-B in mammals and *Daysleeper *in *Arabidopsis thaliana *[12,13]. Non-LTR retrotransposons and *Penelope*-like elements use a combination of RT and EN in their transposition [14-17]. *Helitron *is the only group of eukaryotic transposons encoding rolling-circle replication initiator [9].

YR genes are ubiquitous in prokaryotes but rare in eukaryotes [18,19]. All YRs found in eukaryotes are encoded by mobile elements: yeast 2-micron circle plasmids [20], ciliate *Euplotes crassus *transposons (*Tec1*, *Tec2 *and *Tec3*) [21,22], three groups of retrotransposons (*DIRS*/*Pat*, *Ngaro *and *VIPER*) [23-25], and *Cryptons *[19]. The YR encoded by the yeast 2-micron plasmid, known as "flippase" (FLP), is widely used for site-specific recombination in the FLP-FRT system [26]. *Tec1 *and *Tec2 *transposons encode a DDE-transposase in addition to YR, and therefore the YR domains in these transposons are probably involved in resolving transposition intermediates. To date the only YR-encoding transposons found in the vertebrate genomes are *DIRS *and *Ngaro *retrotransposons. *Cryptons *were originally found in a basidiomycete *Cryptococcus neoformans *and several pathogenic fungi. Their boundaries are difficult to characterize because they have neither terminal inverted repeats (TIRs) nor long direct repeats. Instead they have short direct repeats at both termini. These 4- or 6-bp direct repeats are considered substrates for recombination. By analogy to prokaryotic YR-encoding transposons, Goodwin et al. [19] proposed that *Cryptons *are excised from the host genome as an extrachromosomal circular DNA and integrated at a different locus in the genome. YR typically recognizes recombination sites consisting of two inverted repeats that are 11 to 13 bp long and separated by a segment 6 to 8 bp long [27]. Recently, transposons encoding only a YR have been found in sea urchin, insects and cnidarians and classified as *Cryptons *[28,29]. YR contains four catalytically important residues (R-H-R-Y), but their overall sequence identity is very low among different genes and transposons [18,19]. The conserved tyrosine residue directly binds to DNA in the recombination reaction. In this paper, we report *Cryptons *from various species, including medaka fish, and six human genes originated from ancient domestication events of *Crypton *YRs.

## Results

### The diversity of *Crypton *elements in terms of their sequence and domain structure

**Figure 1:**
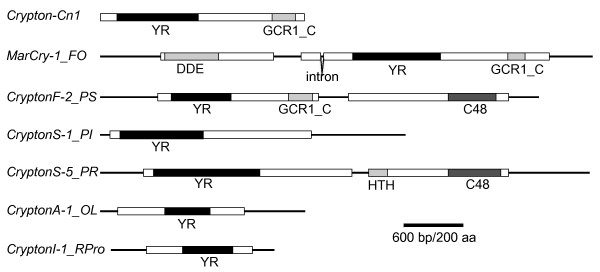
**Schematic structures of *Cryptons***. *Crypton-Cn1 *and *MarCry-1_FO *belong to the *CryptonF *group. YR = tyrosine recombinase; GCR1_C = DNA-binding domain; DDE = DDE-transposase; C48 = C48 peptidase; HTH = helix-turn-helix motif.

**Figure 2:**
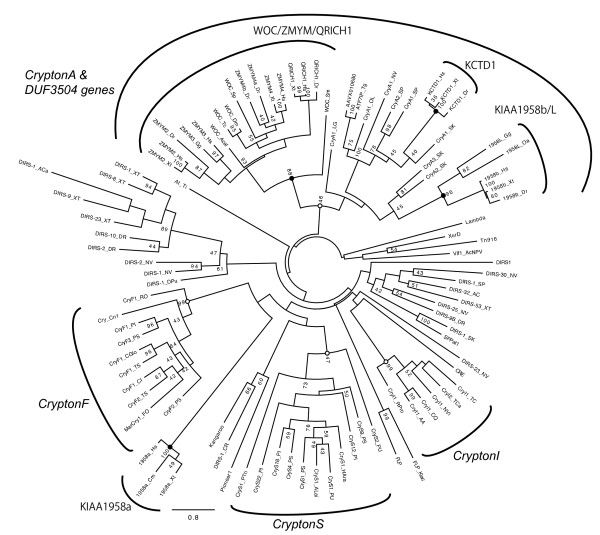
**Phylogeny of *Cryptons*, *DUF3504 *genes and other eukaryotic tyrosine recombinases**. The numbers at nodes are bootstrap values over 40. Open circles indicate the clusters of *Cryptons*, and filled circles show the clusters of *DUF3504 *genes. YR = tyrosine recombinase. Prefixes of names are as follows. Cry = *Crypton*; 1958 = KIAA1958. Accession numbers of *DUF3504 *genes are shown in Additional file 5. Sequences of the transposable elements are deposited in Repbase http://www.girinst.org/repbase/. Other abbreviations and accession numbers are as follows. FLP = FLP recombinase of the 2-micron plasmid in *Saccharomyces cerevisiae *(NP_040488); FLP_Klac = FLP recombinase of the plasmid pKD1 in *Kluyveromyces lactis *(YP_355327); CRE = Cre recombinase of the enterobacteria phage P1 (YP_006472); Vlf1_AcNPV = very late expression factor 1 from the *Autographa californica *nucleopolyhedrovirus (NP_054107); Tn916 = Tn916 transposase from *Enterococcus faecalis *(NP_0687929); XerD = XerD from *Escherichia coli *(NP_417370); Lambda = lambda phage recombinase (NP_040609); At_Ti = recombinase from the *Agrobacterium tumefaciens *Ti plasmid (NP_059767); SpPat1 from *Strongylocentrotus purpuratus *(obtained at http://biocadmin.otago.ac.nz/fmi/xsl/retrobase/home.xsl). Suffixes for species names are as follows. Animals: Hs = human, *Homo sapiens*; Oa = platypus, *Ornithorhynchus anatinus*; Gg = chicken, *Gallus gallus*; Tg = zebra finch, *Taeniopygia guttata*; Ac/ACa = lizard, *Anolis carolinensis*; Xt/XT = frog, *Xenopus tropicalis*; Dr/DR = zebrafish, *Danio rerio*; OL = medaka, *Oryzias latipes*; Cm = chimaera, *Callorhinchus milii*; SP = sea urchin, *Strongylocentrotus purpuratus*; SK = acorn worm, *Saccoglossus kowalevskii*; Dm = fruit fly, *Drosophila melanogaster*; Tc/TC/TCa = beetle, *Tribolium castaneum*; NVi = parasitic wasp, *Nasonia vitripennis*; CQ = southern house mosquito, *Culex quinquefasciatus*; AA = yellow fever mosquito, *Aedes aegypti*; DPu = water flea, *Daphnia pulex*; Acal = sea hare, *Aplysia californica*; Sm = bloodfluke, *Schistosoma mansoni*; NV = sea anemone, *Nematostella vectensis*. Fungi: RO = *Rhizopus oryzae*; CGlo = *Chaetomium globosum*; TS = *Talaromyces stipitatus*; CI = *Coccidioides immitis*; FO = *Fusarium oxysporum. *Stramenopiles: PI = *Phytophthora infestans*; PS = *Phytophthora sojae*; PU = *Pythium ultimum*; HAra = *Hyaloperonospora arabidopsidis*; ALai = *Albugo laibachii*; PTri = *Phaeodactylum tricornutum*. Plants: CR = *Chlamydomonas reinhardtii*.

We identified 94 *Crypton *elements from 24 species representing animals, fungi and stramenopiles that include oomycetes and diatom (Figure 1, Table 1 and Additional file 1). Phylogenetic clustering of *Cryptons *on the basis of their YR domain sequences revealed four groups reflecting the systematics of their hosts (Figure 2, open circles), but two of them were not strongly supported phylogenetically because of the low bootstrap values. Herein we designate them as *CryptonF*, *CryptonS*, *CryptonA *and *CryptonI *to indicate their corresponding hosts: fungi, stramenopiles, animals and insects. *CryptonA *and *CryptonI *are structurally similar; however, *CryptonF*, *CryptonS *and *CryptonA*/*CryptonI *have distinct protein domain structures (see Figure 1 and detailed description in the next three sections). Because of the low resolution of the phylogenetic tree, we could not determine whether there is any relationship between these four *Crypton *groups and to other YR-encoding elements, and we cannot rule out the possibility that they have originated independently.

**Table 1:** Distribution of *Crypton *elements

Classification	Phylum or Class	**Species**^**a**^
Fungi	Basidiomycota	*Cryptococcus neoformans *[19]
	Ascomycota	*Coccidioides posadasii *[19], *Histoplasma capsulatum *[19], *Chaetomium globosum*, *Fusarium oxysporum*, *Ajellomyces capsulatus*, *Coccidioides immitis*, *Microsporan canis*, *Talaromyces stipitatus*, *Neosartorya fischeri*
	Zygomycota	*Rhizopus oryzae*
Animals	Chordata	*Oryzias latipes*
	Echinodermata	*Strongylocentrotus purpuratus *[28]
	Hemichordata	*Saccoglossus kowalevskii*
	Mollusca	*Lottia gigantea*
	Arthropoda	*Nasonia vitripennis *[29], *Tribolium castaneum *[29], *Rhodnius prolixus*, *Aedes aegypti*, *Culex quinquefasciatus*
	Cnidaria	*Nematostella vectensis *[28]
Stramenopiles	Oomycetes	*Phytophthora infestans*, *Phytophthora sojae*, *Phytophthora ramorum*, *Pythium ultimum*, *Saprolegnia parasitica*, *Hyaloperonospora arabidopsidis*, *Albugo laibachii*
	Diatoms	*Phaeodactylum tricornutum*

^a^*Crypton *elements from species without references are found in this study.

### *CryptonF *elements from fungi and oomycetes, and *CryptonF*-derived genes

We identified *CryptonF *elements in nine species of fungi and four species of oomycetes (Table 1 and Additional file 1). These elements encode a protein that includes YR and GCR1_C DNA-binding domains (Figure 1). Most of the fungal *Cryptons *and the five oomycete *Cryptons *are associated with 6-bp terminal direct repeats, which are likely substrates for *Crypton *integration (Additional file 1). In *Fusarium oxysporum*, *Crypton *is fused with a *Mariner*-type DNA transposon and this composite transposon is hearafter named *MarCry-1_FO *(Figure 1). The analysis of four *MarCry-1_FO *copies with more than 97% identity to each other revealed the presence of 16-bp TIRs and target site duplications (TSDs) of the TA dinucleotide, indicating that their *Mariner*-type DDE-transposase is responsible for transposition. *CryptonF-2_PS *from *Phytophthora sojae *and related elements encode a C48 peptidase (*Ulp1 *protease) in addition to a YR (Figure 1). The oomycete *CryptonF *elements are nested in fungal *CryptonF *elements in the phylogenetic tree (Figure 2), indicating a horizontal transfer between fungi and oomycetes.

**Figure 3:**
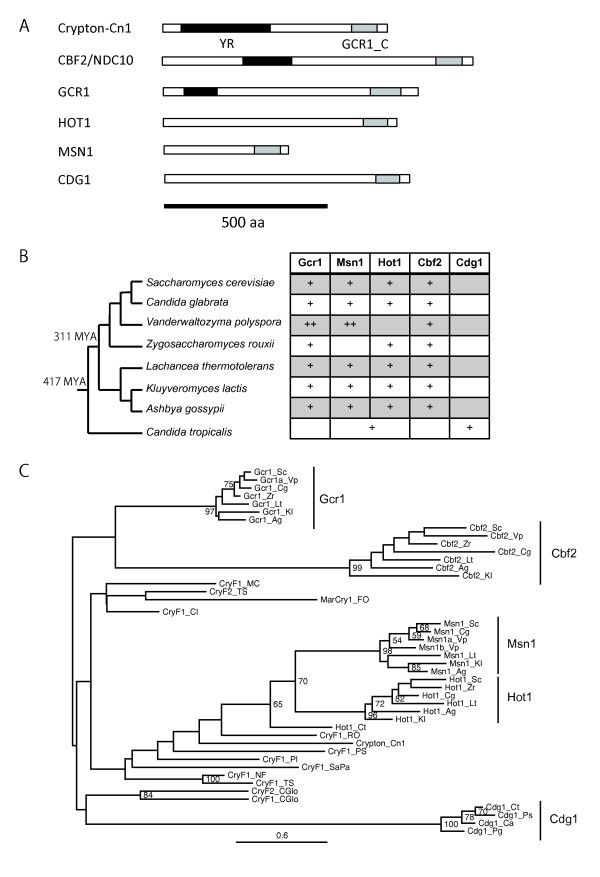
**Distribution and schematic structures of *Crypton*-derived genes in Saccharomycetaceae fungi**. **(A) **Schematic protein structures encoded by *Crypton*-derived genes and *Cryptons*. **(B) **Distribution of *Crypton*-derived genes. Each gene identified in the haploid genome is represented by a plus symbol. **(C) **The phylogeny of *Crypton*-derived genes and *Cryptons *using the GCR1_C domain sequences. The numbers at nodes are bootstrap values over 50. Accession numbers of genes are shown in Additional file 2. "Cry" stands for *Crypton*. Suffixes for species names are as follows. Sc = *Saccharomyces cerevisiae*; Cg = *Candida glabrata*; Vp = *Vanderwaltozyma polyspora*; Zr = *Zygosaccharomyces rouxii*; Lt = *Lachancea thermotolerans*; Kl = *Kluyveromyces lactis*; Ag = *Ashbya gossypii*; Ct = *Candida tropicalis*; Ca = *Candida albicans*; Ps = *Pichia stipitis*; Pg = *Pichia guilliermondii*.

Four genes from *Saccharomyces cerevisiae *were derived from *CryptonF *elements (Figure 3 and Additional files 2 and 3). It was previously reported that the GCR1_C protein domain encoded by *Gcr1*, *Msn1 *and *Hot1 *genes is similar to the C-terminal part of fungal *Cryptons *[19]. In addition to these three genes, we found that *Cbf2*/*Ndc10 *contains a C-terminal domain similar to *CryptonF *proteins. The central portions of *Cbf2 *and *Gcr1 *are similar to *CryptonF *YR domains, but the catalytic site is not preserved (data not shown). *Vanderwaltozyma polyspora *carries two paralogous genes of *Gcr1 *and *Msn1*. *Candida tropicalis *and related species (*Candida albicans*, *Pichia stipitis *and *Pichia guilliermondii*) harbor another gene derived from a *CryptonF *element, represented by XP_002548716 in *C. tropicalis*. It is designated herein as *Crypton*-derived gene 1 (*Cdg1*) (Figure 3). The only domain shared by *CryptonF *elements and all *Crypton*-derived genes is the GCR1_C domain. The phylogenetic analysis of GCR1_C domains (Figure 3C) indicates that *Hot1 *and *Msn1 *are paralogous and that the gene related to *Hot1*/*Msn1 *in *C. tropicalis *represents an outgroup of both genes. Therefore, it is likely that four domestication events (for *Hot1*/*Msn1*, *Gcr1*, *Cbf2 *and *Cdg1*) occurred in this group.

We could not find any *Crypton *insertions in the subphylum Saccharomycotina (including *S. cerevisiae*, *C. tropicalis *and related species). The distribution of *Crypton*-derived genes indicates that *Crypton *was active in the past and that the DNA-binding domain GCR1_C was most likely derived from *Cryptons*.

### *CryptonS*, a new group of *Cryptons *from oomycetes and diatom

We found *CryptonS *elements in seven oomycete and one diatom species (Figure 1, Table 1 and Additional file 1). *CryptonS *elements do not encode any GCR1_C domain, but the C-terminal region is conserved among *CryptonS *elements. *CryptonS *elements are associated with 5- or 6-bp terminal direct repeats. The majority of *CryptonS *elements share TATGG termini. Some *CryptonS *elements encode an additional protein containing a C48 peptidase domain. The peptidases encoded by *CryptonS *and *CryptonF *elements in oomycetes belong to the same family and are related to the *Ulp1 *protease family. Domain shuffling between two groups of *Crypton *elements could explain the similarity, but more data are needed to determine the relationship between these peptidases and other cellular peptidases.

### *Cryptons *in animals (*CryptonA *and *CryptonI *groups)

We identified *Cryptons *in seven metazoan animals belonging to five phyla (Table 1 and Additional file 1). *CryptonI *elements were found only in insects, whereas *CryptonA *elements were found in various animals, including cnidarians. Animal *Cryptons *(both *CryptonA *and *CryptonI*) have no C-terminal domain (Figure 1). We did not find any terminal repeats in animal *Cryptons*. *CryptonI-1_RPro *from *Rhodnius prolixus *hosts a non-autonomous derivative family, *CryptonI-1N1_RPro*, in which 5' 438 bp and 3' 260 bp are 98% identical to those of *CryptonI-1_RPro*. This is the first report of non-autonomous *Crypton *elements. Comparison of 50 copies of *CryptonI-1_RPro *and *CryptonI-1N1_RPro *revealed no terminal repeats (neither direct nor inverted). In medaka, we also found two families of non-autonomous derivatives (*CryptonA-1N1_OL *and *CryptonA-1N2_OL*) of *CryptonA-1_OL*. As in the case of other DNA transposons, *Crypton *non-autonomous elements are much more abundant than their autonomous counterparts.

We can safely rule out the theoretically possible contamination of the genomic sequences from medaka used in this study. First, we identified more than 2,700 copies of autonomous and non-autonomous *Crypton *elements with DNA sequence identities to consensus ranging from 59% to 98%. The nucleotide diversity of *Cryptons *from medaka is consistent with their long-term presence in the medaka lineage. Second, we found many *Crypton *sequences in the database of expressed sequence tags (ESTs) from three different medaka strains: Hd-rR, CAB and HNI (data not shown). We also found several *Cryptons *with inserted medaka-specific transposons such as *piggyBac-N1_OL *and *RTE-1_OL *(Table 2).

**Table 2:** *Crypton *copies containing insertions of other transposable elements

Chromosome	**Start**^**a**^	**End**^**a**^	Element	**Start**^**b**^	**End**^**b**^	**Direction**^**c**^	**Identity**^**d**^
chr1	35100344	35100443	*Crypton-1N1_OL*	470	573	c	0.8614
	35100444	35100648	*piggyBAC-N1_OL*	1	204	d	0.9512
	35100649	35101118	*Crypton-1N1_OL*	1	474	c	0.8728
chr23	4844226	4844361	*Crypton-1N1_OL*	1	147	d	0.9489
	4844362	4844566	*piggyBAC-N1_OL*	1	205	d	0.9854
	4844567	4844980	*Crypton-1N1_OL*	144	570	d	0.9294
chr23	7640079	7640510	*Crypton-1N1_OL*	1	441	d	0.8918
	7640511	7640716	*piggyBAC-N1_OL*	1	205	d	0.9466
	7640717	7640832	*Crypton-1N1_OL*	438	573	d	0.8814
chr5	2033028	2033143	*Crypton-1N1_OL*	3	116	d	0.8966
	2033144	2033348	*piggyBAC-N1_OL*	1	205	c	0.9659
	2033349	2033803	*Crypton-1N1_OL*	113	573	d	0.9132
chr5	22193852	22194261	*Crypton-1N1_OL*	144	573	c	0.9030
	22194262	22194466	*piggyBAC-N1_OL*	1	205	d	0.9707
	22194467	22194605	*Crypton-1N1_OL*	1	147	c	0.9078
chr8	7666866	7667209	*Crypton-1N1_OL*	21	376	d	0.9075
	7667210	7667896	*RTE-1_OL*	2,666	3,352	c	0.9796
	7667897	7668065	*Crypton-1N1_OL*	372	550	d	0.8667

^a^Sequence coordinates in the corresponding chromosome. ^b^Corresponding coordinates of the consensus sequences of repeat elements from Repbase. ^c^Sequence orientation relative to consensus: (d)irect and (c)omplementary orientation. ^d^Identity to the consensus sequences.

### *Crypton*-derived sequences in the *ATF7IP *gene

Identification of *Cryptons *in three deuterostome species (medaka, sea urchin and acorn worm) prompted us to extend analysis of *Cryptons *in chordates, including four sequenced actinopterygian species (*Fugu rubripes*, *Tetraodon nigroviridis*, *Gasterosteus aculeatus *and *Danio rerio*). Although multiple copies of *Crypton *elements were found only in medaka, sequences similar to *Cryptons *were found in various chordate species (Table 3). Most of them do not encode any functional recombinases, owing to frameshifts, deletions and substitutions at catalytically essential residues.

**Table 3:** Molecular fossils of *Cryptons *in chordates

Species	Accession numbers
*Danio rerio*	BX530066*
*Xenopus tropicalis*	NP_001120376, AAMC01135377*, AAMC01082917*
*Callorhinchus milii*	AAVX01521991*, AAVX01068049*, AAVX01132927*
*Ciona intestinalis*	XP_002124034, XP_002125964
*Ciona savignyi*	AACT01002283*, AACT01041791*
*Halocynthia roretzi*	BAB40645
*Oikopleura dioica*	CBY34656
*Branchiostoma floridae*	XP_00260067, XP_002595788, XP_002613958, XP_002613959, XP_002587732, XP_002607491

*Nucleotide sequences including *Crypton *fragments.

**Figure 4:**
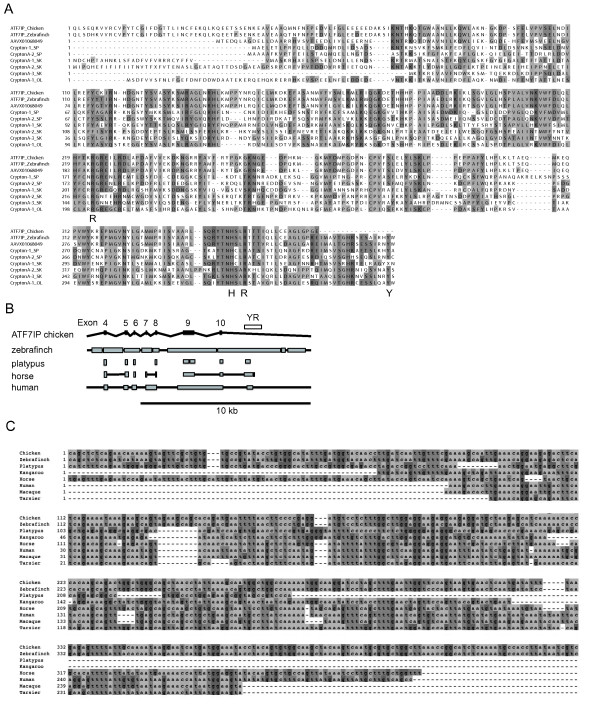
***Crypton*-derived sequence in an intron of *ATF7IP *gene**. **(A) **Alignment of proteins coded by deuterostome *Cryptons *and *Crypton*-derived sequences. Catalytically essential residues are shown below the alignment. **(B) **Illustration of the conservation of *ATF7IP *loci. The position of the YR sequence is indicated by the open box. Black boxes represent exons of the chicken *ATF7IP *gene. Gray boxes indicate conserved blocks between chicken and respective species based on the Net Tracks of the UCSC Genome Browser http://genome.ucsc.edu/. Lines between gray boxes indicate that boxes are connected by unalignable sequences. **(C) **Alignment of nucleotide sequences of *Crypton*-derived sequences.

However, two similar sequences (ABQF01015803 from the zebra finch *Taeniopygia guttata *and AAVX01068049 from the chimaera (elephant shark) *Callorhinchus milii*) include an intact open reading frame of YR (Figure 4A). We did not further analyze the sequence from chimaera, because the sequenced region was only 2,661 bp in length. The *Crypton*-like sequence in zebra finch is inside an intron of a gene coding for activating transcription factor 7 interacting protein (ATF7IP) (Figure 4B). There is a YR sequence at the orthologous locus of chicken *Gallus gallus*, which encodes a protein 97% identical to that of zebra finch, but it contains a frameshift inside the YR region. The orthologous YR sequence from the turkey *Meleagris gallopavo *contains a frameshift at the same position (data not shown). Because the divergence between chicken and zebra finch occurred some 107 million years ago (MYA) [30], this unusually high similarity indicates a strong selection operating on these YR sequences. An exon-intron prediction program would predict alternative splicing in the *ATF7IP *gene from zebra finch, although at present there are no mRNA or ESTs corresponding to the fusion transcript. It is possible that the YR is translated as part of the ATF7IP protein and retains catalytic activity in some birds.

Using the University of California Santa Cruz (UCSC) Genome Browser http://genome.ucsc.edu/, we found that there are partial *Crypton *sequences at the orthologous positions of the *ATF7IP *gene from the human, horse, kangaroo and platypus genomes (Figures 4B and 4C). There are also closely related sequences present in the genomes of rhesus macaque and tarsier. Therefore, the insertion of *Crypton *in the *ATF7IP *gene must have occurred in the common ancestor of amniotes more than 325 MYA [30]. None of the mammalian orthologous sequences encode intact YR proteins, and many mammalian species are missing the YR sequence. This indicates only a slight, if any, selective pressure on this sequence in mammals.

### Ancient domestication of *Cryptons *in animals

**Figure 5:**
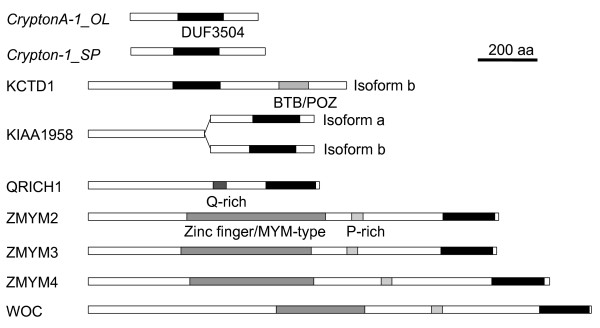
**Schematic structures of DUF3504 proteins**. *KIAA1958 *gene has two isoforms, each of which encodes a DUF3504 domain. The structures of *KCTD1*, *KIAA1958*, *QRICH1*, *ZMYM2*, *ZMYM3 *and *ZMYM4 *are from humans. The structure of *WOC *is from *Drosophila melanogaster*.

**Figure 6:**
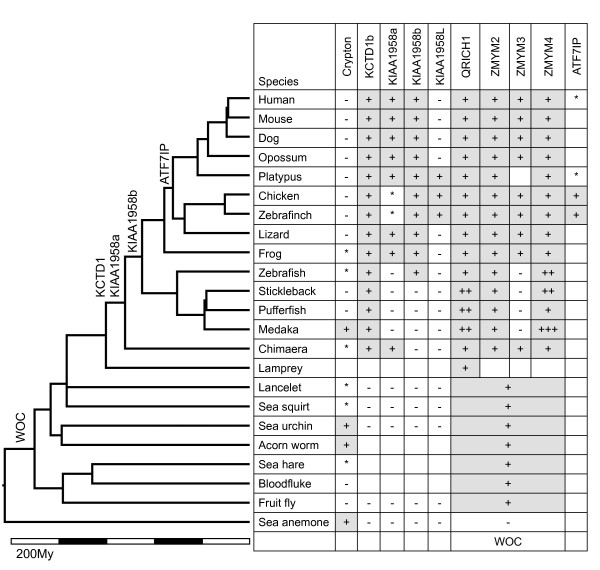
**Distribution of *Cryptons *and *Crypton*-derived genes**. Each gene identified in the haploid genome is represented by a plus symbol. Minus symbols indicate the absence of *Cryptons *or *Crypton*-derived genes. Asterisks indicate the presence of their disrupted fragments. The branch ages are based on TimeTree [30]. The unit of time is indicated. *Crypton*-derived genes listed at nodes of the tree indicate the times of their domestication based on their distribution in different species. *KIAA1958L*, *QRICH1*, *ZMYM2*, *ZMYM3 *and *ZMYM4 *are not shown, because they were likely derived by gene duplications.

Most vertebrate genes similar to *Crypton *code for proteins (Additional file 4). In the human genome, there are seven proteins similar to *Crypton *YRs, which are annotated as parts of six genes (Figure 5 and Additional file 5). The *KIAA1958 *gene contains two isoforms, both of which include YR-derived sequences. The other genes are potassium channel tetramerization domain containing 1 (*KCTD1*), zinc finger, myeloproliferative and mental retardation type 2 (*ZMYM2*)/zinc finger protein 198 (*ZNF198*), *ZMYM3*/*ZNF261*, *ZMYM4*/*ZNF262 *and glutamine-rich protein 1 (*QRICH1*) (Figure 5). A PSI-BLAST search of these proteins against the National Center for Biotechnology Information (NCBI) conserved domain database (CDD) revealed that they share a domain of unknown function (DUF3504 superfamily; *E*-value ≤ 1e-29). The six genes are widespread among vertebrates (Figure 6) and are highly conserved among phylogenetically distant species (Table 4). The phylogenetic relationship of each gene agreed with that of species (data not shown). The nucleotide sequences corresponding to all seven DUF3504 domains were present in the NCBI EST database, indicating their expression. The data clearly show that they are neither pseudogenes nor defective *Cryptons *(see the accession numbers of *DUF3504 *genes in Additional file 5). However, none of them preserve the YR catalytic site. All of them lost the catalytic tyrosine and the second conserved arginine, and all but KCTD1 also lost the conserved histidine.

**Table 4:** Protein identities between DUF3504 domains in two species

Comparison	*KCTD1*	*KIAA1958b*	*QRICH1*	*ZMYM2*	*ZMYM3*	*ZMYM4*
Human-chicken	98% (268 of 274)	99% (282 of 287)	97% (299 of 309)	92% (287 of 313)	85% (265 of 315)	88% (302 of 347)
Human-zebrafish	90% (273 of 304)	86% (245 of 287)	75% (229 of 306)	47% (145 of 310)	-	52% (*ZMYM4a*) (176 of 345) 52% (*ZMYM4b*) (169 of 331)

Numbers in parentheses represent the number of identical residues (left) and the aligned domain length (right). Gaps are excluded in the comparison.

Although the resolution is low because of high divergence and the short length of the YR sequence, animal *DUF3504 *genes tend to cocluster with animal *Cryptons *(*CryptonA*) in the YR phylogenetic tree (Figure 2). There are four independent clusters of *DUF3504 *genes: *KCTD1*, *KIAA1958a*, *KIAA1958b*/*KIAA1958L *and *WOC*/*ZMYM*/*QRICH1 *(Figure 2, filled circles). *KCTD1 *coclusters with several animal *Cryptons*, and the clustering is supported by 100% bootstrap value. *Cryptons *form a paraphyletic cluster, which indicates that the DUF3504 domain of *KCTD1 *was derived from a *Crypton *YR. The position of *KIAA1958a *is distinct from either *CryptonA *or *CryptonI*, and *WOC*/*ZMYM*/*QRICH1 *is clustered as a sister group of all animal *CryptonA *elements. Therefore, phylogeny alone does not support the domestication of animal *Cryptons *leading to *WOC*/*ZMYM*/*QRICH1 *and *KIAA1958a*.

The DUF3504 domain was derived from YR, not vice versa, because DUF3504 lacks the complete catalytic tetrad essential for YR activity. YR is essential for transposition, and repeated generation of active YRs from defective YRs is highly improbable. The distributions of *WOC*/*ZMYM*/*QRICH1 *and *KIAA1958a *are restricted to bilaterians and jawed vertebrates, respectively. Apart from *Cryptons*, the only other possible sources of YRs in animal genomes are the retrotransposon families *DIRS *and *Ngaro *[23,24]. However, all searches of the YRs from *CryptonA-1_OL*, *Crypton-1_SP *and *CryptonA-1_SK *matched the DUF3504 sequence with *E*-values ≤ 8e-12, whereas YRs of *DIRS *and *Ngaro *did not match the DUF3504 sequence at all (even when the threshold *E*-value was set at 100). Several representatives of DUF3504 are actually *Crypton *sequences; for example, XP_001639277 is the protein coded by *Crypton-1_NV*. The patchy distribution of *Cryptons *and the inconsistency between *Crypton *and host phylogenies indicate ancient amplification and extinction events in *Crypton *evolution. The ancient amplifications would have generated many lineages of *Cryptons*, and it is likely that *WOC*/*ZMYM*/*QRICH1 *and *KIAA1958a *derived from lineages of *Cryptons *that are now extinct. We cannot completely rule out the possibility that the two genes and *CryptonA *elements were independently derived from *DIRS*-like retrotransposons or some as yet uncharacterized types of mobile elements, but this implies independent origins of *CryptonA *and other *Crypton *groups (*CryptonF*, *CryptonS *and *CryptonI*). Therefore, four independent domestication events of animal *Cryptons *are the most parsimonious explanation for the origins of animal *DUF3504 *genes.

A representative of DUF3504 from *Halocynthia roretzi *(BAB40645) has orthologs in other tunicates: *Ciona intestinalis *(XP_002125964), *Ciona savignyi *(AACT01002283 and AACT10141791) and *Oikopleura dioica *(CBY34656). They could also represent a domestication event of *Crypton*. Another representative (YP_025778) is coded in the mitochondrion of the green alga *Pseudendoclonium akinetum*. It could also be a candidate *Crypton*-derived gene; owing to the lack of related sequences, however, we did not analyze it further.

All *DUF3504 *genes encode much longer proteins than their DUF3504 domains, and it is possible that the preexisting genes captured entire *Crypton *protein-coding sequences. However, the only recognizable domain encoded by animal *Cryptons *is YR (DUF3504), and there is little sequence similarity beyond YRs among *Cryptons *themselves. Therefore, it is unlikely that any significant sequence similarity was preserved beyond the DUF3504 domains between the DUF3504 and *Crypton *proteins.

### *KCTD1 *gene

The *KCTD1 *gene contains the DUF3504 domain confined within a single exon. Among the vertebrate genes, the *KCTD1 *DUF3504 domain is the closest to the *Crypton *YRs in terms of protein sequence similarity. The sequence identity between the *KCTD1 *DUF3504 domain and the YR of *Crypton-1_SP *is 32%, which exceeds the analogous identity among different lineages of *Cryptons*. For example, *Crypton-1_SP *in the *CryptonA *lineage and *Crypton-1_TC *in the *CryptonI *lineage show less than 30% sequence identity to each other. *KCTD1 *encodes two protein isoforms of different lengths. The longer isoform (isoform b) contains both an N-terminal DUF3504 domain and a C-terminal BTB/POZ (Broad-complex, Tramtrack and Bric-a-brac/poxvirus and zinc finger) domain (Figure 5), whereas the shorter one (isoform a) contains only the BTB/POZ domain. The shorter isoform is approximately 80% identical to the *KCTD15 *gene at the protein level, and related genes are found in various organisms, including lancelet, sea urchin and insects (Additional file 6). The *KCTD15 *gene does not have any DUF3504 domain and is found in gnathostomes from mammals to chimaera. We infer that *KCTD1 *and *KCTD15 *were duplicated from a single gene in the early evolution of vertebrates and after that a *Crypton *copy was inserted upstream of the *KCTD1 *gene, which generated a new transcriptional variant encoding the isoform b. This insertion should have occurred before the branching of the Chondrichthyes (sharks, rays, skates and chimaeras) about 527 MYA [30].

### *KIAA1958 *gene

The *KIAA1958 *gene, of unknown function, is present in two isoforms (a and b) which contain different DUF3504 domains encoded by different exons (Figure 5). DUF3504 domains in isoforms a and b are only 26% identical to each other in the human genome. Although alternative splicing has not been confirmed experimentally, the high conservation of both DUF3504-coding sequences indicates that both encode functional proteins (Table 4 and Additional file 4). Neither of the two DUF3504-coding sequences is interrupted by introns. We found both isoforms in a wide range of tetrapods (Figure 6). Zebrafish lacks isoform a, whereas Chondrichthyes (chimaera) lack isoform b. Some Actinopterygii (medaka, stickleback, fugu and pufferfish) lack both isoforms. Chicken, zebra finch and platypus have an additional gene similar to *KIAA1958 *isoform b, designated *KIAA1958L*. The exons encoding isoform-specific regions of *KIAA1958b *are positioned upstream from those of *KIAA1958a*. The *KIAA1958L *gene likely originated from a duplication of the segment including *KIAA1958b*-specific exons but not including *KIAA1958a*-specific exons, and this duplication event predated the branching between mammals and birds. *KIAA1958L *is less conserved than *KIAA1958 *isoforms a and b. The DUF3504 domains of *KIAA1958L *proteins from platypus and chicken are only 44% identical, whereas those of the *KIAA1958b *are 97% identical between the species. *KIAA1958a *is nonfunctional in chicken and zebra finch, but it is intact in lizard. Isoform b was not found in Chondrichthyes, and it is possible that it originated later in the lineages which branched off Chondrichthyes. *KIAA1958a *might have originated in the common ancestor of gnathostomes. Another possibility is that both isoforms were acquired in the common ancestor of gnathostomes and isoforms a and b had been lost in the lineages of Actinopterygii and Chondrichthyes, respectively.

### *ZMYM2, ZMYM3, ZMYM4 *and *QRICH1 *genes

**Figure 7:**
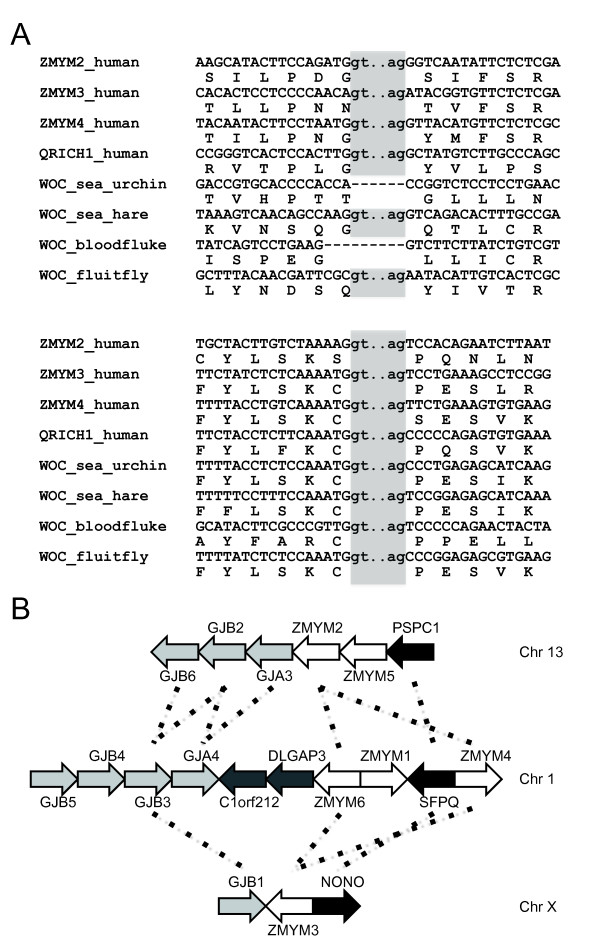
**Paralogous relationships of *WOC/ZMYM/QRICH1 *genes**. **(A) **Two conserved intron positions among *WOC*, *ZMYM2*, *ZMYM3*, *ZMYM4 *and *QRICH1*. Introns are printed in lowercase letters and shaded. Protein sequences are shown below nucleotide sequences. The upper and lower intron positions correspond to the 20th and 22nd introns of human *ZMYM2*, respectively. **(B) **The synteny blocks of *ZMYM2*, *ZMYM3 *and *ZMYM4*. Ohnologous relationships reported by Makino and McLysaght [33] are indicated by dotted lines. *GJB *= gap junction protein β; *GJA *= gap junction protein α; *DLGAP3 *= discs large homolog-associated protein 3; *C1orf212 *= chromosome 1 open reading frame 212. Other gene names are described in the text.

The *ZMYM2*, *ZMYM3 *and *ZMYM4 *genes are present in diverse gnathostomes from human to chimaera (Figure 6). *ZMYM2*, *ZMYM3 *and *ZMYM4 *are similar to arthropod *WOC *in terms of their sequence and structure [31,32]. The DUF3504 domains from the *Drosophila melanogaster WOC *gene and the human *ZMYM2 *gene are 41% identical at the protein level. Some introns are also positioned at the corresponding sites of *ZMYM2 *and *WOC *(Figure 7A). In addition to chordates and arthropods, we found sequence fragments related to *WOC *in echinoderms (*Strongylocentrotus purpuratus*), hemichordates (*Saccoglossus kowalevskii*), mollusks (*Pinctada maxima *and *Aplysia californica*) and platyhelminthes (*Schistosoma mansoni*, *S. japonicum *and *Schmidtea mediterranea*) (Additional file 5). There is no evidence that *WOC *forms multiple gene families in invertebrates. The *ZMYM2*, *ZMYM3 *and *ZMYM4 *genes are listed in the data set of ohnologs reported recently by Makino and McLysaght [33], which means that they were duplicated from a single gene during two rounds of whole-genome duplication in the early evolution of vertebrates before the split between jawed vertebrates and agnathans [34,35]. The synteny blocks of *ZMYM2*, *ZMYM3 *and *ZMYM4 *share several genes in addition to *ZMYM *genes (Figure 7B). The most parsimonious scenario is that the *WOC*/*ZMYM *gene family originated from the domestication of *Crypton *in the common ancestor of bilaterians.

There are three other *ZMYM *genes (*ZMYM1*, *ZMYM5 *and *ZMYM6*) in the synteny blocks (Figure 7B), but they have no DUF3504 domain. The N-terminal part of ZMYM5 is similar to that of ZMYM2, whereas those of ZMYM1 and ZMYM6 are similar to that of ZMYM4. These three genes are present only among eutherian mammals. These data support independent gene duplication events inside each synteny block. It is noteworthy that the C-terminal parts of ZMYM1, ZMYM5 and ZMYM6 derived from transposases of *hAT*-type DNA transposons, but these *hAT*-derived sequences are not close to each other. The C-terminal part of ZMYM6 is close to *Charlie *elements in the human genome, whereas that of ZMYM1 is closer to plant *hAT *elements such as *HAT-1_Mad *from apple (data not shown).

The *QRICH1 *gene was found in diverse vertebrates, including lamprey (Figure 6). The DUF3504 domain in QRICH1 is quite similar to those of ZMYM2, ZMYM3 and ZMYM4. Besides, five of eight introns of *QRICH1 *were at the sites corresponding to those of *ZMYM2*, *ZMYM3 *and *ZMYM4 *(Figure 7A and data not shown). The high structural and sequence similarity between *WOC*, *ZMYM2*/*3*/*4 *and *QRICH1 *indicates that *QRICH1 *originated from either *WOC *or *ZMYM *genes. In the neighborhood of *QRICH1*, we could not find any genes paralogous to genes in the synteny blocks of *ZMYM2*, *ZMYM3 *and *ZMYM4*. However, because *QRICH1 *is present in the lamprey genome, it must have originated at the time close to the whole-genome duplication events.

## Discussion

### Evolution of *WOC*: the third-oldest event of transposon domestication

The most ancient transposon-derived gene known to date is *TERT*, which was generated by the domestication of a *Penelope*-like retroelement [4], and *Prp8*, a spliceosomal component derived from a retrointron (group II self-splicing intron) [36]. TERT retains the catalytic activity of RT, but Prp8 does not. These two genes are shared by almost all eukaryotes. Another example of an ancient domestication event is the *RAG1 *gene [5]. It is distributed widely among gnathostomes, but no *RAG1 *ortholog was found in agnathans, including lamprey and hagfish. Given that agnathans have a different type of adaptive immune system called "variable lymphocyte receptors" [37], the domestication of *RAG1 *likely occurred in the last common ancestor of gnathostomes after their branching from agnathans. Other transposons domesticated in the distant past are in *HARBI1 *and *PBDG5 *genes, both of which are present in vertebrates from humans to actinopterygian fish [38,39]. The *KCTD1b*, *KIAA1958a *and *KIAA1958b *genes are as old as or older than the *HARBI1 *and *PBDG5 *genes (Figure 6). A transposon-derived CENP-B, a highly conserved mammalian centromere, and three CENP-B-like proteins (Abp1, Cbh1 and Cbh2) in fission yeast resemble each other in terms of their sequences and functions, but they derived independently from different *pogo*-like transposases [40]. The human genome harbors a significant number of genes derived from transposons [6]. Some of them were domesticated in the distant past, and there are no traces of related repetitive sequences or TEs from which they were derived. For example, the *HARBI1 *gene was derived from *PIF/Harbinger *and *PHSA *(THAP domain-containing protein 9, or *THAP9*) from a *P*-like element [38,41]. Both *HARBI1 *and *PHSA *were found by screening mammalian genes against DNA transposons from zebrafish. Similarly, the key to our findings of *Crypton*-derived genes was screening of genes against *Cryptons *preserved in medaka, because there are only a few remnants of *Cryptons *left in vertebrate genomes sequenced to date, except in medaka.

The ancestral gene for *WOC*/*ZMYM *probably originated in the common ancestor of all bilaterians more than 910 MYA [30]. This is the third-oldest transposon domestication event known to date, following the two domestication events of RT [4,36]. Our study indicates that domestication of *Crypton*-like elements in eukaryotes was relatively common in the distant past. This implies that *Cryptons *are very ancient and, given their rare occurrence in the genomic fossil record and their great diversity, they were probably much more active in the distant past than in more recent evolutionary history.

### Functional implications for domesticated *Crypton *YRs

No function of DUF3504 domains has been reported to date. Even so, the YR origin of DUF3504 domains implies their functions to some extent. YR forms a multimer when it binds substrate DNA during recombination [42]. On that basis, we can envision two possible functions derived from YRs: DNA binding and protein-protein interaction. There are several indications for functions of domesticated YRs. First, many genes derived from YRs are transcriptional regulators. *Gcr1*, *KCTD1*, *WOC*, *ZMYM2*, *ZMYM3 ZMYM4*, and *ATF7IP *are either transcriptional activators or repressors [43-48]. Cbf2 acts as a centromeric protein directly binding to centromere-specific sequences and is essential for spindle pole body formation [49,50]. Although these proteins usually contain a DNA-binding domain other than DUF3504, exemplified by the GCR1_C domain of *Gcr1 *and *Cbf2*, the DUF3504 domain could also work as a DNA-binding domain. Second, there is an interesting resemblance between functions of domesticated DDE-transposases and YRs. *Daysleeper *is a transcription factor derived from a *hAT *DDE-transposase and binds a specific motif for transcription regulation [12]. CENP-B is a centromere protein derived from the DDE-transposase of a *pogo*-like transposon [13]. In these genes, transposase-derived domains act as DNA-binding domains. Third, a large family of prokaryotic transcriptional activators, *AraC*/*XylS*, shows structural similarity to YRs. The overall fold of the 129-amino acid protein MarA, a member of the *AraC*/*XylS *family, almost entirely recapitulates the YR domain of Cre recombinase [51]. MarA can simultaneously bind RNA polymerase II and DNA to form a ternary complex [52]. These data support the putative function of DUF3504 to be DNA or protein binding.

To date relatively little is known about *Cryptons*. There have been no studies of their transposition, transcription, translation or regulation. The sequence similarity between *Cryptons *is very low, especially in their non-protein-coding regions. We compared DNA sequences of *Cryptons *from different species, but we could not find any conserved nucleotide sequences among them. Furthermore, all *Crypton *domestication events are very old. Therefore, it is very difficult to propose any specific functions of DUF3504 domains. Instead, herein we propose potential pathways in which DUF3504 domains could be involved.

*KCTD1 *and *KCTD15 *are paralogs that have diverged during the early evolution of vertebrates (Additional file 6). *KCTD1 *isoform b, generated by an insertion of *Crypton *upstream of the original *KCTD1 *gene, is widely conserved among jawed vertebrates (Figure 6), although it is unclear whether the agnathans carry the *KCTD1b *gene. The high conservation of *KCTD1b *(Table 4) indicates its essential function shared among jawed vertebrates. KCTD1 represses the activity of the AP-2α transcription factor, and the BTB/POZ domain is responsible for the interaction [46]. AP-2α plays an essential role in neural border (NB) and neural crest (NC) formations during embryonic development [53]. NB is the precursor of NC. *KCTD15 *is expressed in NB and inhibits NC induction [54]. The NC cells are a transient, multipotent, migratory cell population unique to vertebrates. They give rise to diverse cell lineages. We can speculate that by adding a new protein-protein or protein-DNA interaction *KCTD1b *can contribute to the network of NC formation through the regulation of AP-2α.

Among *DUF3504 *genes, the function of *WOC*/*ZMYM *is of special interest because two of the genes in this group, *ZMYM2 *and *ZMYM3*, are linked to human diseases. A chromosomal translocation between *ZMYM2 *and fibroblast growth factor receptor 1 (*FGFR1*) causes lymphoblastic lymphoma and a myeloproliferative disorder [55]. A translocation involving *ZMYM3 *is associated with X-linked mental retardation [56]. Mutations of their ortholog, *WOC*, cause larval lethality in *D. melanogaster *[31].

*WOC*/*ZMYM *gene-encoded proteins are involved in various processes, including transcription, DNA repair and splicing. WOC is a transcriptional regulator that colocalizes with the initiating forms of RNA polymerase II [31,32]. The WOC proteins also colocalize with all telomeres, and mutants of *WOC *are associated with frequent telomeric fusions [31,32]. ZMYM2, ZMYM3 and ZMYM4 are components of a multiprotein corepressor complex, including histone deacetylase 1 (HDAC1) and HDAC2 [47,48]. ZMYM2 binds to various transcriptional regulators including Smad proteins [57]. It also binds to proteins involved in homologous recombination, such as RAD18, HHR6A and HHR6B, which are human orthologs of the yeast RAD proteins [58], and to spliceosomal components including SFPQ (splicing factor, proline- and glutamine-rich) [59].

Interestingly, the *SFPQ *gene is a component of the syntenic cluster of *ZMYM4 *(Figure 7B). The paralog of *SFPQ *in the cluster of *ZMYM3 *is *NONO *(non-POU domain-containing, octamer-binding), which is a partner of SFPQ in heteromers [60]. *PSPC1 *(paraspeckle component 1) present in the cluster of *ZMYM2 *also shows similarity to *SFPQ *and *NONO *genes. In addition to their involvement in splicing, the SFPQ proteins contribute to DNA repair by interacting with RAD51 [61]. They are also recruiting HDAC1 to the STAT6 transcription complex [62]. Therefore, it is likely that WOC/ZMYM and SFPQ/NONO/PSPC1 proteins cooperatively act in transcription regulation, splicing and DNA repair, and that they have coevolved by maintaining their functional relationships. Their DUF3504 domains may contribute to some of the protein-protein interactions.

### Evolution of *Cryptons*

To date *Cryptons *have been identified in a limited number of fungi and animal species. Herein we report the presence of *Cryptons *in new species, but information regarding their overall distribution continues to be patchy (Table 1). *CryptonF *elements are present in three phyla of fungi (Ascomycota, Basidiomycota and Zygomycota) and two orders of oomycetes (Peronosporales and Saprolegniales). Our phylogenetic analysis supports the horizontal transfer of *CryptonF *elements between fungi and oomycetes (Figure 2), which is consistent with frequent horizontal transfer of genes between them [63]. *CryptonS *elements are also present in two oomycete orders and one species of diatoms. Both oomycetes and diatoms are lineages of stramenopiles, and the origin of *CryptonS *elements could date back to their common ancestor.

Animal *Cryptons *(*CryptonA *and *CryptonI*) were found in six phyla: Chordata, Echinodermata, Hemichordata, Arthropoda, Mollusca and Cnidaria. *CryptonI *elements have the same overall structure as *CryptonA *elements and were observed only in insect genomes. It is possible that *CryptonI *elements constitute a branch of *CryptonA *but they have evolved more rapidly in insects. The overall distribution in fungi, oomycetes and animals indicates that *Cryptons *were long present in these three eukaryotic groups, probably with some contribution of a horizontal transfer. It is likely that *Cryptons *originated in the common ancestor of these three groups, although because of the low resolution of the YR phylogeny, we cannot rule out the possibility of their independent origins.

The identification of *Crypton *elements in medaka is surprising. The nucleotide diversity of *Cryptons *in the medaka genome clearly shows that *Cryptons *were maintained in the lineage leading to medaka for a long time. It is possible that *Cryptons *invaded the medaka population after the split of medaka from the three actinopterygian fish species (*Gasterosteus*, *Takifugu *and *Tetraodon*), whose genomes have been sequenced. The vertical transfer of *Cryptons *in the lineage leading to medaka is a preferable scenario because of the domestication of *Crypton *in the common ancestor of bilaterian animals, which led to the origin of *WOC *genes. In most identified host organisms, *Cryptons *are preserved in very low copy numbers (Additional file 1). We found several fragments of *Cryptons *in various vertebrates, including zebrafish (Table 3). The origin of *Crypton*-derived genes took place at different times during the evolution of vertebrates (Figure 6). This is consistent with the hypothesis that *Cryptons *continued to maintain very low copy numbers in the vertebrate genomes and were occasionally amplified in certain lineages.

## Conclusions

This study has revealed the diversity of a unique class of DNA transposons, *Cryptons*, and their repeated domestication events. The DUF3504 domains are domesticated YRs of animal *Crypton *elements. Our findings add a new repertoire of domesticated proteins and provide further evidence for an important role of transposable elements as a reservoir for new cellular functions.

## Methods

### Data source

Genome sequences of various species were obtained mostly from GenBank, and sequences of known *Cryptons*, *DIRS *and *Ngaro *were obtained from Repbase http://www.girinst.org/repbase/. All characterized *Cryptons *have been deposited in Repbase.

### Sequence analysis

Characterization of new *Cryptons *was achieved by repeated BLAST [64] and CENSOR [65] searches using genome sequences of various species with *Cryptons *as queries. All analyses were done with default settings. The consensus sequences of elements were derived using the majority rule applied to the corresponding sets of multiple aligned copies of *Cryptons*. Alignment gaps were manually adjusted to maximize similarity to other related elements. Characterization of *DUF3504 *genes was performed by BLAST searches against both protein and nucleotide databases with known *DUF3504 *genes as queries. We predicted exon-intron boundaries with the aid of SoftBerry FGENESH:

http://linux1.softberry.com/berry.phtml?topic=fgenesh&group=programs&subgroup=gfind and manually adjusted them through the comparison to orthologous sequences in other species.

### Sequence alignment and phylogenetic analysis

We used MAFFT [66] with the linsi option or MUSCLE [67] with default settings to align both nucleotide and protein sequences of various *Cryptons *and *Crypton*-derived proteins. We constructed maximum likelihood trees by using PhyML [68,69] with 100 bootstrap replicates [70] for the amino acid substitution model LG. We also constructed trees with other substitution models, WAG, RtREV and DCMut, and with the Neighbor-joining method, but the resolution did not improve. The tree topology search method was Nearest Neighbor Interchange (NNI), and the initial tree was BIONJ. The phylogenetic trees were drawn with FigTree 1.3.1 software http://tree.bio.ed.ac.uk/software/figtree/.

## Abbreviations

bp: base pair; EN: endonuclease; MYA: million years ago; RT: reverse transcriptase; TE: transposable element; TERT: telomerase reverse transcriptase; TIR: terminal inverted repeat; TSD: target site duplication; YR: tyrosine recombinase.

## Competing interests

The authors declare that they have no competing interests.

## Authors' contributions

KKK initiated the research. KKK and JJ performed the research and wrote the manuscript. Both authors read and approved the final manuscript.

## Supplementary Material

Additional file 1 **PDF file listing *Crypton *elements found in this study**. Click here for file

Additional file 2 **PDF file listing *Crypton*-derived genes in fungi**. Click here for file

Additional file 3 **PDF file showing alignment of *Cryptons *and *Crypton*-derived genes in Saccharomycetaceae fungi in fasta format**. Click here for file

Additional file 4 **PDF file showing alignment of *Cryptons *and *Crypton*-derived genes in animals in fasta format**. Click here for file

Additional file 5 **PDF file listing the accession numbers for *DUF3504 *genes**. Click here for file

Additional file 6 **PDF file showing alignment of KCTD1, KCTD15 and related protein sequences in fasta format**. Click here for file
