# Supplementary material for: Crypton transposons: identification of new diverse families and ancient domestication events
Source: Mob DNA. 2011 Oct 19;2:12. doi: 10.1186/1759-8753-2-12 (PMC3212892; doi:10.1186/1759-8753-2-12)
Supplement: Additional file 1 — PDF file listing Crypton elements found in this study. [file 1759-8753-2-12-S1.PDF]

Additional file 1. *Crypton* elements found in this study.

Name	Species	Length (bp)	Copy number[1]	Terminal repeats
CryptonF-1_CGlo	*Chaetomium globosum*	3597	2	n.d.
CryptonF-2_CGlo	*Chaetomium globosum*	3618	2	TTGRTA
MarCry-1_FO	*Fusarium oxysporum*	5000	15	16-bp TIRs
CryptonF-1_ACap	*Ajellomyces capsulatus*	3953	8	n.d.
CryptonF-2_ACap	*Ajellomyces capsulatus*	4251	4	ATAACT
CryptonF-1_CI	*Coccidioides immitis, Coccidioides posadasii*	4439	19	TAACTA
CryptonF-1_MC	*Microsporum canis*	4611	8	TAACTA/ TACCTA
CryptonF-1_TS	*Talaromyces stipitatus*	3173	6	n.d.
CryptonF-2_TS	*Talaromyces stipitatus*	2873	2	AGGTAG/ AGWTAT
CryptonF-1_NF	*Neosartorya fischeri*	3122	2	n.d.
CryptonF-1_RO	*Rhizopus oryzae*	3484	13	CAGTAT
CryptonF-1_PI	*Phytophthora infestans*	3161	7	GATAAT
CryptonF-2_PI	*Phytophthora infestans*	4137	24	602-bp TIRs
CryptonF-3_PI	*Phytophthora infestans*	2513	10	CTGGTATT/ CTGATACTT
CryptonF-4_PI	*Phytophthora infestans*	2492	15	n.d.
CryptonF-5_PI	*Phytophthora infestans*	4901	3	ATTATC
CryptonF-6_PI	*Phytophthora infestans*	2919	5	
CryptonF-1_PS	*Phytophthora sojae*	4982	7	ATTATC
CryptonF-2_PS	*Phytophthora sojae*	4450	5	n.d.
CryptonF-3_PS	*Phytophthora sojae*	5999	6	n.d.
CryptonF-4_PS	*Phytophthora sojae*	3172	5	n.d.
CryptonF-5_PS	*Phytophthora sojae*	3032	6	TACTTA
CryptonF-1_PR	*Phytophthora ramorum*	1775	3	n.d.
CryptonF-1_SaPa	*Saprolegnia parasitica*	?[2]	1	n.d.
CryptonS-1_PI	*Phytophthora infestans*	3094	29	TATGG
CryptonS-2_PI	*Phytophthora infestans*	3197	21	TATGG
CryptonS-3_PI	*Phytophthora infestans*	3172	14	TATGG
CryptonS-4_PI	*Phytophthora infestans*	3181	10	TATGG
CryptonS-5_PI	*Phytophthora infestans*	3870	11	n.d.
CryptonS-6_PI	*Phytophthora infestans*	3090	3	TATGG
CryptonS-7_PI	*Phytophthora infestans*	2973	14	n.d.
CryptonS-8_PI	*Phytophthora infestans*	3636	11	TATGG
CryptonS-9_PI	*Phytophthora infestans*	3744	5	TATGG
CryptonS-10_PI	*Phytophthora infestans*	3736	19	TATGG
CryptonS-11_PI	*Phytophthora infestans*	2839	20	TATGG
CryptonS-12_PI	*Phytophthora infestans*	3103	4	TATGG
CryptonS-13_PI	*Phytophthora infestans*	3521	10	TATGG
CryptonS-14_PI	*Phytophthora infestans*	3139	27	TATGG
CryptonS-15_PI	*Phytophthora infestans*	3008	13	TATGG
CryptonS-16_PI	*Phytophthora infestans*	3362	10	TATGG
CryptonS-17_PI	*Phytophthora infestans*	3008	30	TATGG
CryptonS-18_PI	*Phytophthora infestans*	5872	4	TATGG
CryptonS-19_PI	*Phytophthora infestans*	4024	7	TATGG
CryptonS-20_PI	*Phytophthora infestans*	3995	7	TATGG
CryptonS-21_PI	*Phytophthora infestans*	3160	6	TATGG
CryptonS-22_PI	*Phytophthora infestans*	3285	18	TTACTG

CryptonS-23_PI	*Phytophthora infestans*	2901	4	TTACTG
CryptonS-24_PI	*Phytophthora infestans*	2828	4	n.d.
CryptonS-1_PS	*Phytophthora sojae*	3319	11	TATGG
CryptonS-2_PS	*Phytophthora sojae*	3419	4	n.d.
CryptonS-3_PS	*Phytophthora sojae*	3549	9	n.d.
CryptonS-4_PS	*Phytophthora sojae*	4300	4	TATGG
CryptonS-5_PS	*Phytophthora sojae*	3180	22	TATGG
CryptonS-6_PS	*Phytophthora sojae*	5306	8	TATGG
CryptonS-7_PS	*Phytophthora sojae*	3307	7	TATGG
CryptonS-8_PS	*Phytophthora sojae*	3083	4	TATGG
CryptonS-9_PS	*Phytophthora sojae*	2915	11	TATGG
CryptonS-10_PS	*Phytophthora sojae*	3016	25	n.d.
CryptonS-11_PS	*Phytophthora sojae*	2930	4	TTACTG
CryptonS-N1_PS	*Phytophthora sojae*	1436	7	TATGG
CryptonS-N2_PS	*Phytophthora sojae*	1443	6	n.d.
CryptonS-N3_PS	*Phytophthora sojae*	1656	11	TATGG
CryptonS-N4_PS	*Phytophthora sojae*	1319	13	TATGG
CryptonS-N5_PS	*Phytophthora sojae*	1281	2	TATGG
CryptonS-N6_PS	*Phytophthora sojae*	465	32	TATGG
CryptonS-1_PR	*Phytophthora ramorum*	2904	10	TATGG
CryptonS-2_PR	*Phytophthora ramorum*	2846	12	TATGG
CryptonS-3_PR	*Phytophthora ramorum*	3761	4	TATGG
CryptonS-4_PR	*Phytophthora ramorum*	2960	6	n.d.
CryptonS-5_PR	*Phytophthora ramorum*	4966	3	TATGG
CryptonS-1_PU	*Pythium ultimum*	3130	4	TATGG
CryptonS-2_PU	*Pythium ultimum*	2885	5	TAATG
CryptonS-1_HAra	*Hyaloperonospora arabidopsidis*	4344	12	TATGG
CryptonS-2_HAra	*Hyaloperonospora arabidopsidis*	4191	10	TATGG
CryptonS-2N1_HAra	*Hyaloperonospora arabidopsidis*	700	15	TATGG
CryptonS-3_HAra	*Hyaloperonospora arabidopsidis*	2827	17	TATGG
CryptonS-1_ALai	*Albugo laibachii*	2541	5	TATGG
CryptonS-1N1_ALai	*Albugo laibachii*	686	16	TATGG
CryptonS-2_ALai	*Albugo laibachii*	3505	1	TATGG
CryptonS-2N1_ALai	*Albugo laibachii*	755	5	TATGG
CryptonS-1_SaPa	*Saprolegnia parasitica*	?[2]	1	n.d.
CryptonS-1_PTri	*Phaeodactylum tricornutum*	4671	4	CTGTAAG
CryptonA-1_OL	*Oryzias latipes*	2073	7	n.d.
CryptonA-1N1_OL	*Oryzias latipes*	554	705	n.d.
CryptonA-1N2_OL	*Oryzias latipes*	353	95	n.d.
CryptonA-2_SP	*Strongylocentrotus purpuratus*	2263	8	n.d.
CryptonA-1_SK	*Saccoglossus kowalevskii*	1248	3	n.d.
CryptonA-2_SK	*Saccoglossus kowalevskii*	2289	7	n.d.
CryptonA-3_SK	*Saccoglossus kowalevskii*	1504	5	n.d.
CryptonA-1_LG	*Lottia gigantea*	1970	12	n.d.
CryptonI-1_RPro	*Rhodnius prolixus*	1657	9	n.d.
CryptonI-1N1_RPro	*Rhodnius prolixus*	709	83	n.d.
CryptonI-1_AA	*Aedes aegypti*	1980	3	n.d.
CryptonI-1_CQ	*Culex quinquefasciatus*	1528	2	n.d.

1 Copies with >90% identity and >50% length are counted.

2 We found a single copy in the sequenced genome, and therefore the length and sequence identity are uncertain.
