# Supplementary material for: Crypton transposons: identification of new diverse families and ancient domestication events
Source: Mob DNA. 2011 Oct 19;2:12. doi: 10.1186/1759-8753-2-12 (PMC3212892; doi:10.1186/1759-8753-2-12)
Supplement: Additional file 2 — PDF file listing Crypton-derived genes in fungi. [file 1759-8753-2-12-S2.PDF]

Additional file 2. *Crypton*-derived genes in fungi.

Gene	Species	Accession numbers
Gcr1	*Saccharomyces cerevisiae*	NP_015250
	Candida glabrata	XP_447030
	Vanderwaltozyma polyspora DSM 70294	XP_001646487
		XP_001644747
	Zygosaccharomyces rouxii	XP_002496603
	Lachancea thermotolerans	XP_002554024
	Kluyveromyces lactis	XP_455009
	Ashbya gossypii ATCC 10895	NP_984616
Msn1	*Saccharomyces cerevisiae*	NP_014525
	Candida glabrata	XP_445780
	Vanderwaltozyma polyspora DSM 70294	XP_001642661
		XP_001644772
	Lachancea thermotolerans	XP_002551517
	Kluyveromyces lactis	XP_451326
	Ashbya gossypii ATCC 10895	NP_983944
Hot1	*Saccharomyces cerevisiae*	NP_013895
	Candida glabrata	XP_447184
	Zygosaccharomyces rouxii	XP_002499250
	Lachancea thermotolerans	XP_002556033
	Kluyveromyces lactis	XP_453563
	Ashbya gossypii ATCC 10895	NP_985426
Msn1/Hot1	*Candida tropicalis MYA-3404*	XP_002550141
Cbf2	*Saccharomyces cerevisiae*	NP_011656
	Candida glabrata	XP_447688
	Vanderwaltozyma polyspora DSM 70294	XP_001643026
	Zygosaccharomyces rouxii	XP_002498831
	Lachancea thermotolerans	XP_002555136
	Kluyveromyces lactis	XP_454115
	Ashbya gossypii ATCC 10895	NP_985870
Cdg1	*Candida tropicalis MYA-3404*	XP_002548716
	Candida albicans SC5314	XP_712451
	Pichia stipitis CBS 6054	XP_001384672
	Pichia guilliermondii ATCC 6260	XP_001485136
