# Supplementary material for: Crypton transposons: identification of new diverse families and ancient domestication events
Source: Mob DNA. 2011 Oct 19;2:12. doi: 10.1186/1759-8753-2-12 (PMC3212892; doi:10.1186/1759-8753-2-12)
Supplement: Additional file 3 — PDF file showing alignment of Cryptons and Crypton-derived genes in Saccharomycetaceae fungi in fasta format. [file 1759-8753-2-12-S3.PDF]

Additional file 3. Alignment of *Cryptons* and *Crypton*-derived genes in Saccharomycetaceae fungi in fasta format.

```
>Cbf2_Sc
MRSSILFLLKLMKIMDVQ-------------------------------------------QQQEAMSSEDRFQELVDSLKPRTAHQYKTYYTKYIQWCQLNQIIP-----T
PEDNSVNSVPYKDLPISAELIHWFLLDTLITDDKPGEKREETEDLDEEEEN--------------------------------------------------------------S--------FKIA
TLKKI-------IGSLNFLSKLCKVHE--------NPNANIDTKYLESVTKLHTHWIDSQKAITTNETNNTNTQ-------VLCPPLLKVSLNLWN
PETNHL-SEKFFKTCSEKLRFLVDFQLRSYLNLS-FEERSKIRFGSLKLGKRD---------RDAIIYHKVTHSAEKK------DTPGHHQLLA
LLPQDCPFICPQTTLAAYLYLRFYGIPSVS---KGDGFPNLNADENGSL----------------LQDIPILR--GKSLTTYPREETFSNYYTTVFRYC
HL-PYKRREYFNKCN--------LVYPTWDEDTFRTFFNEENHGNWLEQPEAFA-------FPDKIPFDFKKIMNFKSPYTSYST-----NAKKD
PFPPPKDLLVQIFPEIDEYKRHDYEGLSQ-----------------------------------------NSRDFLDLMEVLRERF------LSNLPWIYKFFPNHDIFQD---
-----PIFGNSDFQSYFNDKTIHSKGSPIL--------SFDILPGFNKIYKNKTNFYSLLIERPSQLTFASS--------------HNPDTHPTQKQESEGP
LQMSQL----DTTQLNELLKQQSFEYVQFQTLSNFQILLSVFNKIFEK----LEMKKSSRGYILHQLNLFKIT----L---------------------------------
----------------------------DERIKKSKIDDADKFIRDN---------------QPIKKEENIVNEDGPNTSRRTKRPKQIRLL------------SIAD------------------------
-------------------------------SSDESSTEDSNVFKKDGESIEDGAYG--------------------------------------------------------------------------------------------------------------
--------------------------------------------------------------ENE--DENDSEMQEQLKSMINELINSKISTFLRDQMDQFELKINALLDKILEEKVTRIIE
QKLGSHTGKFSTL----KRPQLYMTEEHNVGFDMEVP--------------------------------------------------------------KKLRTSGKYAETVKDNDDHQAM
STTAS--------------PSPEQDQEAKSYTD---------EQEFMLDK-SIDSIEGIILEWFTPNAKYANQCVHSMNKSGN-KSWRA-----NCEALYKE
RKSIVEFYIYLVN----HESLDRYKA-----------------------------VDICEKLRD---QNEGSFSR-----L-------------------------------------------------------------------
----------------------------------------------------------AK--FLRKWRHDHQNSFDGLLVYLSN-----------------------------------------------------------------------------------
--------------------------------------------

>Cbf2_Vp
HN----------------------------------------------------LPTQCIKMTASSNMDVDLQVTNLMGSLSARTANQYKSYYTKYIKWCHSKGFILD----T----E
DTEFVYKHLPVSSNLFHWFLMDDFITNTDAADSHSTIESSNLDHESV-------------------------------------------------------------S---------LKIGTLKK
I-------VSSLKFLAKICSIHKEG--------TEDVHIDEKYLESIIRSHTYWYNELTAFNNTINMT------------NTHPAILKVSINLWNPQTLNL-S
DKIFKTGLEKSRFLVDFHFTNYLHLN-YSKRSIIKLFQLNGNKEK--------------NCITLDQYDETS-----------RTHSPLLLLPQSCPFLCPI
TSLATYLYLRFYGIRNVY---KGDGFPDLSNTIKQSKMDYSSVTKLSANLIHWLDLPLIR--GKSPLDYPKDETMSNYYSIVFRYCHL-
PYKRREYFQKTR--------VEFPTWTDEEFNEF------DNILKESMSTA-------YKYKVPYDFAKILNLKSPFIPYDN---VDNSLTNESMLPA
SLLDQLFPEIDQYKRHS-NILSK-----------------------------------------EAKNFLNLMETLRNAL------LKNLPWIFHFFPHHDIFKD---------SLFS
NSDFQSYFQEVIGNSVKQNPM-----NQLPFDILPGINKYTEN--DIYDVLIEPPLKTTSISS--------------STLVNLPSIQSGIASNLDTSSI---
-----ISDEIMGPAFQFVQYQTASNFKLLLTTLSKVFNK---LDMKKSSREFLNQQMNSLSNI-----L------------------------------------------------------
------VEKINSSKPSDVKKIESGNDDEENLKIEELQSKE------------------KEHAKPKPKFGLL------------DLDS--------------------------------------------
-----------SSSEESEEESDREDINE-------------------------------------------------------------------------------------------------------------------------------------------------------
------------------------------DNE--DDEDRDMQEELKFMVNELVGEKVRATFKIQMSEYEKKLKMSMEKLVEEKISQTLKTQTSTIEGTINDI
LDKRKRDQDRIEKEEEEERERNRI--------------------------------------------------------------KKAR-------------------LEPVSV--------------ELPALESNKQR
EEN---------SLKFIINS-DIDTVEGVILEWFTPNAEFNNESIHSMNKKHG-KEWRI-----PFKSLYKERKIIIEFYIFLVN----QKQVDRYKA
-----------------------------VQICESLRTKKDTKDKSLSS-----L----------------------------------------------------------------------------------------------------------------------
-----AH--YLKDSKVANNNSFEGILETLSS-------------------------------------------------------------------------------------------------------------------------------
>Cbf2_Lt
M------------------------------------------------------------PDVPSRPMDPHLHIKALVDTLAPRTAHQYKLYHTKFIQWCRDNKLLRR--------DPGADH
PYRDIVVTATLVHCFVLSQYVCVSDS--------------------------------------------------------------------------------Q---------FSASVLRKM--------ISAFKFLHR
VCCAYEP---------DYPYELDHDYLEAVARLHVSASAEN-----------------------PPFSPLHLVSVNMWTPHSNRL-SEKYFKGGLERLRFLVDF
HVQQYWHLP-FADRARLKLGDLRFAADP--------------GMLHVVRQGQHGA-------DAPETVQQLALLPQRVPWICPLVSLAAYLYLRFY
GAPKAY---KGDGFPDLLGADE--------------------WAFLPMVR--GKSLDKYPREETMTNYYAHVFRHCHL-PYKRREYFYNKS-----VDYC
QYPEIRRTELDQLQEI------APAQQREL-------FPHLIPLDFLRHMNHFPVYEPPSE-----NHYADLANVPKSLLVQVFPEIEEYKRSS-IP
LGE-----------------------------------------QAQHFIDALQLLRSAL------VSALPLFYHFFPEHDLFKD---------PMFQNAEFQGYFHQK-----------------
---------------------------IEQLRATDQLQSYDPAA--------FGLVEAERDFAAPSQPPQALNVPPSADTQDLQIYLRDQTFRMVQFQTTSNFHL
LLQSLSRIFEK----LETKKSNREYIIHQLGSLEQT-----L-------------------------------------------------------------QDQIAASKPQDVKTEEETSSSSTN
GPKQETRESLPAGKLSRP-----------------------------------------------------------------------------------------------AVFDSDAEDDDDYKEEDDNE---------------
------------------------------------------------------------------------------------------------------------------------------------------------------------------DND--DEVDPNLQNEL
QALVSQVMDTRFKSAVEQQTAQIERHVQSLISAQVKEEVRKQLAHLLNQQSST----------------------------------------------------------------------------
-------------------------------PTPQPSQQPVTIAP--------------KRPREEQEPPSTAG---------ESTFAMSP-QLTSIEDIILEWFTPNPEQGNECVHTM
NKKFG-KTWRA--GSQEATHLYKQRKLIIEFYICLVN----QRQMDRYEA-----------------------------VAVCEKLR-----DNASIQE-----F---------------
--------------------------------------------------------------------------------------------------------------SN--MLKSWKKGHNNSFDGLG-----------------------------------------
--------------------------------------------------------------------------------------------

>Cbf2_Cg
M--------------------------------------------------------------------ISKDNEIRKLMESVAPIKVHQYKSYYMKYIEWCEQKELLSF--------ESNSSIPYENV
PLLPQLVHLFLLECVIKWPIV-------------------------------------------------------------------------------A--------SDVNELQIY-------IESFRFLKKLCDIHS
E--------VDPNSEFDDYITDVLELHRRWDSLLKDESQS----------------SQLNKMATLSINMWNSNTQEL-SDKYFKTSMEKLRFLTDYH
FYTLLNWP-YKERSKLKLSHLKVHNVE---------------------NDENSLNAYRLIIINDDGAVQGTVIPHDCPLICPITTLAAYLYLRFYGVKPI
Y---RGDGFPELTK-----------------------DFDLPLIR--GKSLKDYPREETLGNYYSSAFKYCSL-EYKRRVYLHGSSDEKNGVKNIRYPDTSN
PAYKEFLTD--YPNNEEMKFDRL--------FPAHIPLDYERIFN----YSNYDGVQDGLTIPEITELPPNDLLVQVFPEIEKYKRESYNILTT---
--------------------------------------KSKEFLKVLEVLRNVL------VINLPWIYRYFPDHEIFSDQT------SIFQNSDFVSYFNERIALISNNDSTIDIN
SIPPPLRNIPGYHTGLIENNIMLQYLVEPNFKAGVKSDSIPSVHDSIYNIPVTGNEGQSSSVALAPPITN-------------NSGWKKEAFKLV
QFQTLSNFNPMIDTFKKVFEK----LDMKRSTREFIINKFETLTKY-----V-------------------------------------------------------------NARLNSVTSEEIT
SYFDDILEKKNGLRYINKESNSVDKQISGRDVVDNLKRKKQKQKRRFKLLSV----------------------------------------------------------------------DDS
SSGTDDANEDSVNDSSTESDSST---------------------------------------------------------------------------------------------------------------------------------------------------
------------------------DDL--HEEENAMQEQISSMIDELVTKKLSTLFDVKFEQMEMKLESLVKNTVSEKFNHYIAQESKKRSYEGDFEID
NTKKIRKSIDDGIKE--------------------------------------------------------------------------------------------------KF--------------NDKVNTSSTGAEEE---------HFV
FRMAD-TLDSIDEIIQEWYTPDPKQGNMCVHSMNKKYK-SKWRT-----GFEKIYRERKPIVDFFIYLVN---MEKLSRSKA------------------
-----------LSICWELKS---QNNFTVSE-----L----------------------------------------------------------------------------------------------------------------------------SA--YLK
SWKEKNNSTFVGLLNQIQHT------------------------------------------------------------------------------------------------------------------------------
>Cbf2_Zr
M------------------------------------------------------------------------EEIRKLVDTLSPRTAHQYRSYCKRYVEWLVQKGIVEQ----ELREETLDAEQYAKL
PISTQLVHWHLIDTMG-------------------------------------------------------------------------------------T---------GSLPSIRKC-------ISSLKFLDKLCSIHREV
DASTGDTAAVTKLDEKYLENITRVREVREQEEEQG--------------------QLAQNLHIISANLWNPHSQL--PEKYFRNCFEQLRFLVDFQW
RLYTKMS-YMQRSRIRIAELVGHADG------------GGISVELDTWSQLGEYVNGVEPQLRSRQPVIVVSHANPLVCPLVSLASYFFLRFH-
```

--------DGGGFPELRDANGS------------------WREMPIVR--GKSPGDYPREETLGNYYAAVFRYCRL-NYKRREWFGQSK--------LQYPSW
RPEEYEFFKRY-------QPELQEA-------FPMNVPRDFVECFNLGVGRGTVDD---------KIVGGRRDLLVQLFPEIESYKRHG--GLDQ------
--------------------------------DAQRFVDLIEVLRIHL------LRALPVLYRLFPDHDLFKT--------DTLSKPDFKSFYADVETNDYGMGELVLGQ
G--LQENERERESVPIPAPASSSDV------------------FQLVQFQTVSNFEILLKLLSQIFDR----LEMKK
SSREFAIHQLSLVHDT-----L---RDRIKSSKPGDEKGFGQE---

--EDE--EEEASEDEEELKNMVEQLVNAQIRT---QVSALENRLERLVDEKIDAKLEKLLG
KRSAEEDG---RV-------------EKKLKPDLEPKE----------
PSVFKLDP-DIESVEAVVLEWFTPNPMMGNECVHSMNKKD--KSWRN-----GFESLYKERKTIVEFYIYLVN----HRGMDRYKA--------
-------------------VDLCERIR-----GGGDLTD-----F--AQ--L
VKDWRREH-ETFDGLGDL---
>Cbf2_Ag
M---ELQLDRAFSRFSSRDVHRYKSYVKQYLEWCRERGLDVE-------------SCSAAELWG
SARRLHWFLQSKWGSG--I--------ESVAEMKSV-------VNCIQLLGREL------------
-ATGAQLDKAYIDNVVRLHECTSVLHENG-------------------ALRGQFEKICVNVWNVRTPSL-KEKYFKTCLDKVKWLLDYQLNYYTN
AA-LDERKTWVLKDFEVAEEA----------------------------------VLVVRRRWAVLPQPHPLFCPLFTLAVYLHLRFYGVKKQY---RGDGFPDL
SRPDL-------------------WEELPIIR--GKSLTKFPRVETLGNYYPAVFQYCQL-PYKKRLYFQGRL------EEPVFPAMNDDASGLDEQY-------
--------------FVKGVGRDFILGMNRYRLQDKFPS----------IEIPDDPVLYELFPDLDRFETEQ---TARPFVQ
MMILLRKVM------FRSLPVLYSCFPEHDLFQD--------PIFKKPQFIAYLNAADSLCDVSVPL----------------------------HLLVEPPSTTHATA
SAAPA---------VTQTSAPAAPNGSVAPVQQAANPP-------DADLRKDTLQFVKDQTLSNFTILIQLLSKLFEK----VETRKSNKLMIRSEL
DALHST-----L--KRKISSLDALPAAPHDDERPTKK--
---HKSSSPQDISNLSDLDDQSA---
--GDDEPDDVEELQQLVQQLVQRQVT---QASQYILDKVRSDIRDIIREELASLG
RAYPSP--AA--------------DPKPAPAEPPSHKG--------P
PVFRLQS-DLATIEEIILEWFTPNPSFDGECVHSMNKKYG-KAWRT--NSGQEPIYKSRKVIVEFYIHLVN----DLGIDRHDA--------------
-------------VDHCERLKA-----SQTPLE-----F---SQ--WL
KTYKQTHGNCFPNIT---
>Cbf2_Kl
MS--KLDSLLKELPTRTAHLYRSIWHKYTEWLKTMP------------------------DLTGADLKL
FLSQKYI--V--------KYIASHDDIAKDPLPTCDAMIWFSRALDIE-----------NND
VLVLQQRLYGLVKLLEFDYS------------------------NVIAILQKISINLWNPSTDSL-QSKHFKTCQDKLKLLLDFQWKFNTNVS-FEDRTT
VSLKDLQCILDD--------------------ENGKCGL------AHSSKPNFVLVPNFQSPFTCPIFTMAVYYYLRFHGVKKYY--KGDGYQILSQ-------
--------------LEHIPIIR--GKSLDQYPRELTLGNWYPTIFKYCQL-PYTKKHWFQVNQ------EWPQFPDFSDSSENTSTLAESDSE----------
------NTIGIPDFYIEKMN-------------------RTKLQPCPQVHVHLFP--TDLPPDIQAVFDLLNSVL------
VTSLPLLYRVFPTHDIFLD--------PSLKTPQNIAFLTGTLPLDIESQE-----------------------------HLLAQLIDKTGTVSELVPNP---------VKID
QNEHTLTPIGTSLSQTDIPM-------LDQLKTELQKLIQLQTSTGFSQLITVLLEIFQR----LDFKKSNKQFVIDLLQSCRKD-----M---------
--RNKLMDPCSLSTNFADELSDDE-----------------------------NEKGNKTGAIYDP------------ETDN-------------
--GNEESVSDYEPSFKKSKPNY---
--TEE--PLSDDENMEEVAQLVNQLITRKFNENLEQQTDRIISSIQPTLRDMVRTEVADAL
NLSLREASRDS---VN--------------KKNSDENFQNYSLT---
-----EDHFEMNP-DCSDIKSIILEWFTPN----RECVHSMNKKYG-NKWRL---TEPNLSLYRIRKPIVQYYIHLIN----VENLNKFDA--------
-------------------LNKLEAVL----EKHHSVPL-----L--SS--
FLEKAKKNGYASVT--
>Hot1_Sc
MSGMGIAILCIVRTKIYRITISFDYSTLMSPFFLFLMMPTTLKDGYRMNSQVNEDAIGINLDLSLPTHISPTTGS----------------------------
--ESASGSNASTLRNDGNALDG
GLLRTSAAISAPTGTSQPTETIGEKLSNEERVNSNVSASNSTTAGT-------GRMLSQSLTNDSPSNEIST-----------------------------DQLKIF
QRMDEM---SARMIEME--
---ESFNKLSNKIAEQNTMVLNLKQD---------------------------------------NY
KVMNKLNILLKLV-------AQPSARPSTNNAQNK---
--------------------------------------LAIEL----LNSISAVSSAYLQKMQNNGSG-RQHTADLCTGDSNTHSGINQHRTTNGTIDVNTNTAQLNN
QFSNALNTILPDQQHNRNNVSQNINQSLPNRQLGPVINTQANQNQSQVLIHNTNTHQQVNRSPIS------FPNASTDKPFKLNPNGI
KRRRRNTQSNNNASTNDHASAAQKPISALSPLTNSHNSTTSMNYTNSSIH------------SGVTSASNSFHDLNSLNNFGTTTALSL-------
-----------------------PSLALDNASFPPNQNVIPPIINNTQQPLSFSQLINQDSTTSELLPSGKSGVNTNIVNRNRAST-----------------------LPSY
PKPMTVKSNVDDDGYQEDDDDDGDDEGDGRD----------------------------NEEDSTAEEDEVDDEIETDMKN-------------------------------------
--ASINKRRRSLHHKKSNSLNGRR-------------------KLHGESATKPNINS--------DL
HYRILK-APTDVKTIWEEYDT--GIRGKPSIKHLEAKYG-NKWR----LNKNKKTFSRRKRLYKFI---------LNGMERGKTA----QEM-------
-----------IETLENKRLYKDDEDGEVKKRTIGWL---Q
E--SLAGI---
>Hot1_Lt
M-------------------------------------GSSAPAMANGNQASEVRGSLSNDALNLGLNLALPADTHDTAKTVG--
--TVSGSSLADILGSNATPQAGGENGHASGTSNA
TPASALNEPTHAQIHSASPAQNAGIGP------------------QRYGIIQQVSGSSELGTGNSTT-----------------------TSVQICQRMDEV----------------
--SARMIAME---
---EMFNKLYFKINEQEATIQHLRLQ---------------------------------------SEAFLTSILSEVRQV-------
NHNMPILDNPDDNEKDG---
------FVTDL---LNSITNVSSNYLKKVNTKHSQ-KYKRQRQSMGGAGAASTDKPSKDAFGEAATSSSTRAINGTAGVAPNNGASAGQ
ANLAA--QSSKRAAPDSEHSKTHNSSFAESGGQTG---QQLVQESHSYPVSEASQLPLPHYLTQQSFTLNPNGIKRRRANT---------------
---ATKPSFSQSHQDISSLNGIGSMSL---PNLTLENLTRKNQPHSLGFNVN
SDHNVSAGAR--------LRHRNIEIQVGSDNVDSHELD---------------------LDSSEDEEGYQEDDEDRNSDRSAEAA----------------------------D
AGRSYGARGNVINAAEEAEDETESYGGLFEPRNRSPGQQIHQRREDRRNFDRG--
----------RPVRAPGPTEQPLKATQVNAETKTAAA-------------PRSGGPAADEHHTR----LRERDLNYTLLK-APANVRAIWQEYIE--GIDG
QPAVKFLEEVYG-NKWR----LKKNVKTFARRKRLYKFI---------INGIKHGRSA----DDM-------------------IKILEDKRIYK-NEHGEVKKR
TIGWL---QQ--SLSGI---

>Hot1_Cg
MS--
---DAGDSKLAKEQSDASTG-------TCNGNGTGNGTGSGTATANATTTPTPTPTSIPTAAG
SVTDVTA---TSSMVLAKLDDI---
------------------------SSRLTMLE--SNFNNVF
SKINDQNSMILDLKQN--------------------------------------NSHAFLRLSSKVNKL-------ETHVAALASNNPQSA--
---FVTDL---LNSITNVSSSYLRKMKHNGAE-NLNV

QLSTHENGFVHPTPND---SPNMMGPIYYNQIETSKARGQLKSLN----------------------------------RKKTFTLNPN
GIKKRRLGHHHGNSGSRSNNLNVTFDITGQVGQPGYDTSLNSATPNNHAMTP----------SNVISSSNSYSELQSLNGLQSNTTASD--
------GRTLSPVNTVQALKTPYLTATNQHSLNHGSLNNYNLNFPQFLDNTNSVTLSRLGSSSPVEKRDGIV-------MSHSAPQLDNGS
ITYPDNRLHIRNQASQDILGGASQGQPQKI-NNIDEDGYQEDDEEEESELNKHKNKVGNLKYNTTLTDSHSINDDRKPTVNVYAS
TGHPVNNTKSEYLEDDESNDSDNSHETDEESEDEVEEYDEEDDMVNEEDRQPYS--
----------RKPKEPASERKRKKLRKINKYRNNVIE------------PRKKDDKEKDAQNS-------DLNYMLLK-APSSVKTIWEEYVN--GIDG
NPSIRGLEEKYG-NKWR----IKRNKKTFSRRKRLYKFI--------LNGIDKGKTA----DEM-----------------IDMLEKQRLYR-DENGEIKRR
TIGWL--QQ--SLIGI--

>Hot1_Zr
MSDTTNPLVVGGTTAMTPNGNGLLDTSQPVTNVQVGDGPSPSLNMTTAAGGSASGQQPEPLALNLNLALESDP----------------------
---LAANNHTAPNNNNS
SRASTAGQTSTNTAAAAAAAAVAAANSTGNASTNMNSNSNTTMPTPVMEGSNPGSTNSAGLTGTYPSMNSIPSASPSRTIPNDRST
-------------NTVRMFQRMDEL--SARFIVME-----------
---EMFQKLCKTVEQQSLCMADLKLQ---------------
--------------------NSQMCQEVSGKLDEIC------QQQRHYGEQPNDQDS---
--FVTDL----LNSITNVSSSYLRKIRSRSRP-GSTKVSP---
-------QQQGITPPGGQWTQYDQAATTQNFAQTQSNSVP---------------------CDKMFTLNPNGIKRRRHNPPH-----------------------------------
-----------------SNVTSGAPSYTDLASLNNLGTISLPNL-------ALDHTGIT--------------------------------PLIRSGGSGGANGPLGFPTHPTQDQQ
QQQPQQPQQPPQHSSRGRGIHLEIGSEL--------------------------ASANEDEDGYQEEDDDDDNSVLAKTHSSSDGGSSS-----------------
SEDASAEEEDEADDEIDNATKESSGNRSNNVNLNARAKRKRPRLADQNMRNLIGNSDDVSVRNTPD------------------------------------
-------------------------RNERLVLKNSRSAIGGQLVGSSTNSLPD------------VVGNNVVNTSPNSR-------DLNYTLLK-APTDVRTIWKEYV
S--GIGGDPPIKKLEEKYG-NKWR----LNHNRKTFARRKRLYKFI--------INGMNKGKSA----DEM----------------IDALERRRLYR-DEN
GEVKRRTIGWL--QQ--SLTGI-----------------------------

>Hot1_Kl
MS----------AMENRKNSLHGSPNGLSGLNMVLPREGDSNNGIMTTEDARNGKLKGDKDLNMKMHVGMGTTPINTRLGNTMNSD
VANSMSYLLEQPQSGTLIGDRLILETSEEPVGVNLV--
---------DGQNARNNQQMLNNASHMDPAFTLPADDTMNNSAAAAAAAAAAAAAASTGPSQPTMQSTPSISTGGSTN---------------------
----------------------------FQVQIAQRLADI--DQRILRM
E--MLMDNVCNKIDSHSQQQSLWKND
------------------------------------MHQMENKVIDVLDEI------KSNIFSLKRQVSNQGDGA--
---FAAEL----INAISNVSNKHIRKNTYGFPA-GNPQGNDMG-------------------
------------------------------QTMNGYNNAMQLSSMAAPMSYVPGEQVNLNQLGNSKDQIDRFLTKSANE---------FMLDPSGLKKRRKN
PSSSNES--PITLSHTKTHSTSQPINYSASL---PNLNLDALSKVML
PQHQQLTHNGGSQHPLSDKSIKFGISSTSHSSSNSSDSDDEEDD----------------------IDDEEDEAEDEEDENDDSRDVNDHDN------
------------------VEGRETLTKKSMKPGNSIEGRDPSGTNGTVNSTTNNHMNTIDTATNTSSQLQGTVIRSTADTENPKQ-----------------
--ESNASAAATSGAATGVATGTTTANIAT------------SASDKLKLHGDSKK-------EPKFTMIK-APSSV
KEIWREYTQ--GIDGRPSIKSLDQKFG-NKWR----ANKNKKTYSRRKRMYKFI--------LNGIKKGKSE----EEM----------------VQMLE
NKRIYK-DSDGNQKKRTIGWL--QQ--SLSGI--------

>Hot1_Ag
M------------------------------------IDPVTHIVTPSNDEQRQGRSDGGMQLGLEDLHGGGGGLGQQK--
--VSGMAEGTDSIVDVLTNPSSSVNLHSNILSVSTN
VEEGQGMQPAAIQGGGTAAGRRFSSVPISDSPVGCGQHGMGTASTQVPSHGSTANLLSMGNPVA--------------------TNVQVCQRM
DEV---SARLIVME---
---ETVNKLLGNIDAQQQQIASMRSE------------------------------------SMEMLGS
LMKEVREL-------KQQVPVLDDPDNNEKDK--
---------------------------FVTDL----LNSITNVSSNYLKKVSSRNLQ-RFKRQRDIM---STSDLPS
LGNTQSSSHFMQDLVNPYASTQDFQSFTVLKD--------KAKEFTLNPTAINKRRRLSAQPLN---TL
QMSQDQLQQQRGSVQSLISIPTSGQ------------------------KNANFGPQLQNYLIGLQNIPMSEAEKVSSPTVLKQFQNSTTDPDLKTG
RRNPGLDFQIGSGDDENDEND--------------------------GASGEDEDGYLEDDE---------------------------------------GYRSQSQRTMSSEDD
EQFTGGNQGEDEAGPRAAGRNPKSLPSSIVRRFANSGSQ--VSVSGTPETERL
VLKNNDKYDKNTMPA------------PNTKPRAVLASNPE----DTLTDVQYTMVK-APDSVRAIWDEYTI--GVGGKPSIRQLEQTYG-NKW
R----TKRNKKTFARRKRIYKFI--------LNGVRNGKSE----SEM----------------IDILESRRVYR-TEDNELKKRTIGWL-----------------------
--QQ--SLSGI--

>Hot1_Ct
M--
---NETYNSQESNTSMYYNGNSTQTTGNNTQPNNTNGNPSTEAASPT
AINNHSINDIYLVLQSLQGQFNKFQNIHFRELLLKVNYLQD----------SLEHVKQSVQDLN--
---QQMIYAVQNNNNNGNNN---
-----------------------------NLMSSNNYMLNNQFKAFEVFTKH---------------------------------------LNKLNKEIEEI------SSGTPVQQSPTTHH
HHQQQQ---QRSPH
NVSNQPPRQIQQQPSS-SQQQSQIPQQP--QSSLPPLPAQQLPSQVNQFGSTHLSVPSTNHGSSQ
FDEEVDNMARKNLS--LRTTASTSPNPTSHYVFDVGPFGSRNM-------NG
NTSNHSLYDKPPIPPDQQQPPQQQPPSDQSQEPTRKKRKPSRK---SVSQD
AFRNHTAESIQQNARQSPPQQQQSSTQRQQQQQSQQQQQQQQQQVRNTGNTSSFGNTYILPMPPSTSGGNEATSQFILPRDANG
TVMTSALRQTSNQTRSSTSQPGTSVSTTGSTAAATAAFPFDDEI--ESIEGLDNDGSTNGSTTSNGGG
TTIDG------------SNKKSKHKADDQQNKVILTALDVPQYKLER-SLKALSDIWKEYAH--GMNNKPPLKSLENKYG-TKWR----NETES
RTFLRRKKIYEAI--------ENGMSKGYTE----DQV----------------IEELEEHRTY--RKNGSVKRKPLSWL--
--ST--NMPEKFSTPN---

>Msn1_Sc
M---
---ASNQHIGASNLNE--
------------NEAILTNRVAEL--ERRMSMFE-------------
--GIFHALSNRLDLHFKKYDVVVNS-------------------
-----------------QQQQINELTAFLSTLL---NDQQRHAEILSE--
---KLSGT---LHGVSATSISLSQTLDP---
---QGFTDGTTAPGAPRNYTSVPMNNDQTAHPQNEGAVSNETLFE--DILNGNSQ
ENDKSQQQTNSSNSISQEN-------NSTNPSV---

--DTRFNKPQNYNSNLVPSLEE------YSANPPNNDGGQSQGLYISSNSS-------------
-----------------------------------QSRQSPNLQKVSPNHENAVESNAQESVPTF--------EEEQYETKTGLKRK----RIVCTRPFEFIK-SPH
SVMEVWKEYTE--GVNGQPSIRKMEALYQ-TAWRR---DPAVNKRYSRRKVLWKAI--------QTGLNRGYSL---NYV----------------VEIL
ENSRY---VNDKQKVKQPIGWL---CHSSHIPETLK--

>Msn1_Cg
MN--
---REGISGPGGAIITHDSVSTGSSSNSKNNGGNNNNSNIPNGNSSMG------
--DDIMVLSRINDI--
----ERRMMMFE--NMFHALSGRLDHHF
KKYDVLVSS-------------------------------QQQQISELNAVITTLL---NDQYRHAEFVRD--
---KLSHS----LHGISSTSISLNGISNQ----------------------------------
--------------------------NNDNRSNGFANNNSSGVASGSSNGVNSQGNTNNNSNRSSSTSHLNNPNML----------------------------------
--------------------------GNNSSGTGNNHNNNSVGASNSNQSVTH-------TILDDILNEHD---------LQDKKYSGNRNPGDMNSRNDV
NDK--SQQSPRDPMSLKKTKFI
HHRQYNDVTDGDSVI------PYTNPYVDQSGHQQNNLTSNILFNDSTSG--QNAGNQGTQQATQ
NREEGMPSSP--------------GRKSYSSKRGPKKK----KVMYHKPFQFIK-SPHSVMEIWKEYTE--GIDGQPSIREMESLYS-TGWRR---
DAAVNKRYSRRKVLWKAI--------ETGLSRGYTL----DYI---------------IDLLENYRI---IDPEKNTKQPIGWL------------------------------
---CQVNNIPDLLK---

>Msn1_Ag
MDIQANM--

-----ELGGLERRVLDL--ERQISMYE-------------------
---RLFQTFSAKLDHHFKKYDLVINA-----------------------
---------QQQQINVLTDIVSTML---NDQYRYAGILRD--
-------------------------------------KLRGS----LDGIVTTSTSIRGMQPN---GG
MPEQEPKPHDDDAVHAAHRANHHHAAADDGNRDLTNVDAILGEFIPPQVSPDENELHAAPAAPVPDAQT---PAP------------------
------------KRNGGKKHPKGSVEHKFIISNGLKVPR-------RAVDPPAKRR--
--REDPQKEYEFRDAEFSALA------SDGHPLDAQSAPPA
PQHHDYDHDDRALPL---QRADRPLSTGASPDPSAPSSSRLNSNI------------KEEQYYTHRGLKK
K----RKIYVGKFEFLN-SPQTVLDIWKEYTE--GFNGQPSLKDMETMYQ-TSWRR---DPAVNKRFHRRKVLCKAI--------ERGLERGY
DL----QDV----------------VRVLEDSRL---IDASRNLKQPIGWL---
------------CQGVNIPDLFK--
>Msn1a_Vp
MS--

DDTQLDKRVSDL---ERQISMFE-----------------------
---KMLLTLSGALDQHFKKYDLVIST----------------------------
----QQQQIVDLNAVISTLL---NDQFRHTEITRE---
----------------------------------KLSST---LHGISATSISVANTINTEHRV---PSYSSMKN
VSNQNSISISKPNNESINNSNPDQRTNLHILQSLPTRTTTA------------RTTTISSSSSSSTVPTTGLNTTAPTTITSSNGTANNDSSMS
ENAVTDLTQATQLMNTSSLVGPNQTNTSSTDAINASHHDLNDNR-------INVNSILQQSESTGSLPIHIGRQDNKNSSIAPQFTDNH
DNDIHFT--DVSNSVLGNSSST
VKNGTNTTSTTLSSSIV---QGNPTIVPSVDASSLAKSISSTSTNSPSVTKDGLAMLQLGKNDSGNNTGNNNNDE------------------------
-TRNEGISDTNENQDDNEISGTGTPNNE------------DDDIFKSKSGRKKK----KNVYVGDFQFLK-SPHSVMDLWKEYTE--GINGQPS
IRELESIYQ-TGWRR---DPAVNKRFSRRKVLYKAI--------ETGLNRGYSL----DYI----------------IKILEDYRY--VDRNKNQKRPIGWI---
--FHSANIPDILR---

>Msn1b_Vp
MN--

NMSLLINRVQDL---ERQVLLYE-----------------------
---NIATNLSDDLDKHFKRLENIISS-----------------------------
--QQSQINDLNKSLLNILDKKNLRTPNKNIMNT--
-------------------------------------NMNTTGGVSVNTSNSTSLSNSNITTPSYKY-SPPQMPTLRHHSGPLMAPTVPVPLPHQPLQILPQ
QQQQLIDPYSSVGAATGSGAGSVTGSTTPLIVNPIISTTGPQQQPPSSSSSLSSNPHQQQQQQQQQLQQEHKNILVNH---------PLS
FRSHSNVNISNTNPGSNANTLLLRPYIPGSLGNSS----------NMAPQLPLALDNHNSNNNNNNNNNNNNNNNRHINNNNHNNNNNH
NN-------NNNHNNNNNNNNNSSDTSHSHNHSHNHNNPSITSSDNNNNNRRSTSLS--
--TVNLQTNNNGRLGSRNSAPMVVMTNPSNKIQFPGNSGNYIPTSSLISKSHSNPHAHPHI
QQNPGLHPYPNNAGAIHYTNEDHLGFVQLPHLQQSIIVNPHTT------------------------TNNSLTNVETNIKGGNGGSNGPGNTNIG
LGVNHHPYHHQRHQQQHQHHTNSKNP----RTIYHGNFEFIK-SPQTVLEIWKEYTE--GFHGQPSIKEMESIYK-TGWRR---DPAVN
KRYARRKVLWKAI--------ENGIAKGFSL----EYI----------------VQMLEDHRY---TDKSKGIKEPIGWL-------------------------------------
---CQKSSIPEIFK--

>Msn1_Kl
M--

DDSMLQKRVEGL---ERQISMYE----------------------
---RLFQSFSSKLDHHFKKYDVLVSS---------------------------
-----QQQQINILTEALSTML---NDQYRYSEILRD---
-----------------------------------KLGTA----LSSVSATHVNIAGLSNQRMQD---NQKQQ
QQQQQQQQQQQQQQQQQQQQQQQQQQQQQQQQTQPLSNVTAFLEDFIAPDVPLSNAVPRRAKLGSFQTKQTTALNPTP----
--------------------------DQDTGFR---
---PLEVDVPVPSGVAVEDSQQQVQQQQIQNNSGSGKRKHRTTN
GDGSSSKK--INRSLPMPDSTSADGHRQSQEPQASHI------------ADEKYYTSRGQSKK----RKVY
TGNFKFFN-SPQSIMDIWKEYTE--GFEGQPSIKEMEQMYH-VSWRR---EPAMNKRYHRRRVLCKAI--------ERGLEKGFHL----DDV
---------------LRMLEDARV---IDAERGTKHSISWI---
CQFSHIPEILR--
>Msn1_Lt
MD--

DVSPLIARISGL--ESQIAMYE-----------------------------
---KLFQTLSAKLDHHFKKYDTVVNA---------------------------------
----QQQQINSLTDVLFTLL---NDRCRYADIQRE--
--KLGNT---LAGVASTGASLSGVVAT--AGLGRQ
PKQATGPQTSSRHVSGLQSDHQSASLYAEDTPSRRE---

---------------DERRSSDSYYSDNSVTSAE--SDESEDRENSEL
-------------RPELHESADAAHKK----RKLFDRKFRFLA-SPQSVAEVWKEYTE--GLAGQPSLKEMESTYR-SSWRR---DAAVSRKFYRR
KVLCRAI---------ETGLARGYAL----EEI------------------IDMLESHRQ---QREGRQDKLPIGWL--
--CRRSNLPSIFK---

>Gcr1_Sc
M--
---VCTSTSSNFYSIA
QYILQSYFKVN-VDSLNSLKLVDLIVDQTY-------------------PDSLTLRKLNEGATGQPYDYFNTVSRDADISKCPIFALTIFFVIRWSHP
N------------PPISIEN------------------FTTVPLLD---SNFISLNSNPLLYIQ
NQNPNSNSSVKVSRSQTFEPSKELIDLVFPWLSYLKQDMLLIDRT-----------------------------------NYKLYSLCELFEFMGRVA------IQD
LRYLSQHPLLLPNIVTFISKFIP-ELFQNEEFKGIG--SIKNSNNNALNNV-TGIET
QFLNPSTEEVSQKVDSYFMELSKKLTTENIRLSQEITQLKAD----MNSVGNVCNQILLLQRQLLSG---
---------------------NQAIGSKSENIVSSTGGGILILDKNSINSNVLSNLVQSIDPNHSKPNGQAQTHQRGPKGQ-----------------------------------
-----------------------------SHAQVQSTNSPALAPINMFPSLSNSIQPMLGTLAPQP---------------------QDIVQKRKLPLPGSIASAA------------T
GSPFSPSPVG---------ESPYSKRFKLDDK---PTPSQ-----TALDSLLTKSISSPRLPLSTLANTAVTESFRSPQQFQHSPDFVV-------GGSS
SSTTENNSKKVNEDSPSSSS---KLAERPRLPNNDSTTSMPESPTEVAGDDVD--REKPPESSKSEPND
NSPESKDPEKNGKNSNPLGTDADKPVPISNIHNSTEAANSSGTVTKTAPSFPQSSSKFEI---------------INKKDTKAGPNE----------AI
KYKLSR-ENKTIWDLYAEWYI--GLNGKSSIKKLIENYGWRRWK----VSEDSHFFPTRRIIMDYI---------ETECDRGIKL----GRFTNPQ
QP----REDIRKILVGDLEKFRI---NNGLTLNS-----L--
SL--YFRNLTKNNKEICIFENFKNWNVRSMTEEEKLKYCKRRHNTPS---

>Gcr1_Cg
S--
---------------------------AFAFIMNFLGLSPITKGDTTNVSKTNKADEESVSNLNRGMSAPSLSTSIFAMPRTIPTTNNDKQKQNWNVISE
NQTNPAQETIDARKFNEDATSPKQVDITNNKLKSLLLKQEEH---------SNINSADTTTNFYAISQYILQSYFKAN-SSDLASLKLVDLI
VDQTY-------------------QDSLTLRKLNETTAMQPYQYFNTVSRNPDISRCPIFALAVYFVIRWSHPN------------PPITINN------------------Y
HLISLLD---SNFIDPNAKLVNNENPDHQQLSQNTKILRSEIFHPSP
DMIELVFPWLPALKQDMLLIDRT-----------------------------------NYKLFSLCELFEFMGKVI------IQDLRYLSQHSMLLPKIVEFISKFIP-
ELFENEQFKSSS--EDEFMMERQGSDFQPNSYDTNLQSI-SREGTQNGYINNSVLEKMI
ESQFVSLSKKLTTENIRLSQEITQLKSD----LNSVTSMCNQILQLQRKIANG--ENQT
RDRSMGNSVNGEGNIIILDKNALNSQLINQLVHSADDT---SQQKGLES
SQLAPIGTFNSLQSKLQ--------------------------------NSEAQKRKLPFPSHQAATSNIVNVPMNNANGGASPYSPSINMQP-------DSPYNK
RYRLEDK---STPSQ-----NALDSLLSKSVASPRVGGSSVNGSNIMDSPRSNRNNSIDKKFFMTGQYNSPGGNQGTFTDGSFNRILSDI
PYKAASREETIIKPLSPNVPILPVNTTIS---------HRRQQGALKKLVERPTLPITESTTSLPPSPDAQSAIDSN---QLPKPQLASRASTIV
DPD--------------LSVEVMHTIPAPGHSNLQHTLEKGYEGKSSRDDIEKNKKMETQK-------------RRKGDGASKPEHLP--------PLKYKL
SR-DNKTIWDLYAEWYI--GLNGQPSIRKLIEDYGYRRWK----VSDDSHFFPTRRVIIDYI---------ETECDRGIKL---GRFTNPNQP----R
EDIRKIIVGDLERFRI---NNALTLNS-----L---SV--YF
RKLIKENNEICIFENFNNWQVRAMTEEEKIRYCKRQHGPADRNVTTTNESHSSVTDSQRPNMIAKQTFNNPIENPTTTSIERESS
SSSEKNSSPRQEDAEVGNTLPKNADADDDRNNEHNDNVTNNEFNDKDETA
>Gcr1_Zr
MNLDPFDRNRISSS--
---NRNASSPSSSSSQAIPLFPLNNISNAN-------------------DDLY
HVLNSQDQQ---------ARISSTNISSNFYLITQYILQAYFKVS-FTELRSLKLVDLIVDQTY-------------------AESLTLRKLNEGASVRSYEY
FNTVPRQEDITRCPIFALATYFVIRWSHPN------------PPISVEN------------------FDRIPLLD---
-----------------------------------PTTITWNKGFQELNHTKEGYKVSRSVSFEPANELADIIFPWLPSLRQDMEIMDRS------------------------------
-----NYKLHSFLELFEFMARTI------VQDLKYLQLNSGLLPNIVTFVAKFIP-DLFQHPKFQKAK--
---PVFNERGGDDEDSHFLQLSKRLTTENVRLAQQITQLKTD---LSNVQYMCDQILKLQQQQLVN------
---NDRNYRQNNNNNNNGVIVIDKNSVNSSIWNNLAQNTAAVSASGGNNS-----------------
--TSNNEEPQESSNLAPMLASFPLVN------------------------------------PEVNRKRKLPPP--------------
---QPGLSPFLPSPGPVLPTVA---ESPYTKRLRIDDK---RTPSQ-----SALDLLLSKTASSPRFPSLPNNHVAKMPSRY--------------------SSP
TAFAMTGSPSAVLPPNPVSLAPR-TTQPEAHSQPRNISGPIVQNSPTITAAT--AARAAPATVSAIDNAS-SKNPNTNSLPNTPIVNNGA
DEP---NEEAEEDASEEDGRDDEN------DDSKALSPKSSEARPRPVS----SQQVPSSNPDNTSSGPNNK-----------------------QNRPMEKL
GPNR----------HIKYKLSR-DNKTIWDLYTEWYI--GLNGQSSIKSLIETYGLRRWK----VSDDSHFFPTRRIIMDYI---------EMECDRGIK
L----GRFTNPDQP----REDIRKIIVGDLEKFRI---NNGLTLNS-----L--
---------------------SM--YFKNLTRENKEICIFENFKNWSVRAMTEEEKNKYCKRQHTKETL---
----

>Gcr1_Lt
MNS---
---TSMASRPKSGSNSPLVVGNRNQTPSPRSSTSEMSGSGKN-------AH
RTSLLPQL----------------FASPQLNMSVAMQYVLQRFFEIQDASLAQSLRAVDLIVDQTY-------------------AESLSLRQLNDSFSNKQYRY
FNTVSRNKQVSKCPIFCIAAYAAARWGDHR----------NGLSIRFEG------------------FQNVPLLG---
-----------------DTSSFAALAASQVAGVSPSP-------------DEEPSPSSVWGKEKTVRTLEPPEDLINFVFPWLHNLQDDLETKDRT------------
-----------------------NYNLHSLCELFEYLARVL------IQDLAFLSC-TNELPSLLSNILEYVP-KLKSSSAFQQFK------------------------------------
--------------------------------------SEMQRQISSR------TKQSEWSDRVLSRVEESYIEISRRFVLHNQSLSEEIRELKSE---LRSMKQLISELL
SSQRQFTSF---NIPNGALSANGPNSTGTMGSVEKNALPTNLFNNLVTSGEDP---------
--AQTTPSLAPINMFHSLSSSA---------------------------------VPETQRRKLPLPN
H-------------------SGFSPPP-----------IGSPFKRFKFDDP---KSNQQQNNLNATLDSLLSKAAVSPRLAVPSLNSGPVSSQLS-------------------
---PQFSSSDAHTQLLLKKPP---
---------------------------TSSVLSGGSTHE----------VFKYKLSR-ENKTIWDLYTEWYV--GLNGKPSIKSLIDTYGWRRWK----VSEDSHFFP
TRRIIIDYI---------EREIDRGLRT---GRFLNTD-----REAMRKVITDDLEKFRA---ANGLTLNS-----I---
---SM--YFRNLTRKNTEICIYDNFQDWSIMLIDEEEKNKYCKRQQNSGA-------------------
----

>Gcr1_Ag
MDT---
-------------------------------------I-------NLSTNLHDW------LEDPMLQVSDSRGAGS-------VGASTTLSPPGMKHLELSQSNPTPSPRSSVS

MNRG----------SHCRSGLYEL---------------VQRVPLDENSLLAYVLQQYFHVHEKKEAELLTLVDIILDQTY------------------PHSLTLRK
L--RYDSTYFRYFGTISRENDITRCPVFYVSLYFYMTWGLPA----------RKQISWDT-----------------FSTIPLLS-----------------------------------
--NPPGDEYGPGASCSKPESQNHSMSNKITR--SVSVPAQLKQSVYPWLAQLKEDMNQKDR
V----------------------------------NYKLYSLIELFQYLSECI------VQDIAFLEC-TGQLPAVRELLREGNQ-KLFGSAMFAKYK---------------------
--EEMRQQLDGE------VVNKDWHNSILLRMEEKFNQLTAKTAQDNTKLNSEISDLKGK----LN
SMSSMISQLLESQRQLISR--SSSHVPALSGTAVSALDKALQQPLSLN--------------
--DTASALAPISVLPSLSQQ--------------------------------HLADGKRKLPLPSA
PSPLDHS-----------------------------AGATLKKFRFEDRVLEGTAGTPNNLGSPLEPLLSRAISSPRIPITSLTTQIPPTLAAATP--------------
---PLHVRPQNTRILQEPRQAADPTTAAMTLTQDCINTVMAD--
---------------------ELELQKAPLDESVHNLAV------------NNTASSNGGPNE----------SIKYKLSR-DNKTIWDLYTEWYK--GFDGKPSIKS
LIERYGWRRWK----VSDDSHFFPTRRIIINYI---------EKECDRGVAM----GKY-----PSTLDREAVRKLVAKDLENFRV---TNSLTLNS-----
L--SL--YFRNLTREQREICIYNNFTDWSLLILPEEE
KTKYCKRKQGSSSE--

>Gcr1_Kl
MDK--
---------------------------------------D--------NLNINLQDW-------LNDPMLAAQLNRAQSSTP------TIFNGMDDTANGSSVVQQKSDPTKTQLQ
SVDNASA----------SELSNFL-------------------MNSRLNLGTLMKFVCIEFFKCN-KDELANLRLLDLTQDSTF------------------PQTLTLR
NM--QTENSHYKFFNTINRDKSITSCPVFAIALYSFITWKQTA-------------ISWKN-----------------FMNLPLIF----------------------------------
------------------------------------DSKDIIMLQGNNQLRLPVYTS----------------------NMIRSTVTPKKEMLSFVFPWLPALERDYLNHDRS----
------------------------------NYILFSIVELFYFLAKIV------LQDFAFLQC-TGQLPHLQELIKSELSWQFLESDDWNLFK-------------------------
---DEMQRQIDNE------LLQMDCFNSVCGKIEDRFTKLSKDLVTENDKLSNELRTMKSQ----MASMT
AVVNQIFHTQRQLLSY--QIHN--PANNITSTMANFNSQAMNSTNNVTNNENNNVNSNTMI
NTSNNTMIP--TSNTNPNIAYSVNYSKMM--------NDKRHIYNQP---
----------MNSIKRMKLD------------------GKDMSNFD---------------SSSRRLSQPQP---QSPLQ---IGSPLEALLSKPIPSPKITVSMLNNSVA
SPPPN--PMAMSPLPYISEPIELNNNGNSISDGGQRNVNVEPSSSLNHVENASNVRSNAQTTSK
INSLGSNEK--DELRNGNDSENEPKVMTRN------------NSKGKRTGNPNM---------DIKY
KLSR-DNKTIWDLYNEWYH--GLNGKLSIKELIEKYGYRRWK----VTEDSHFFPTRRIIIDYI---------ETETDRSINL----KRF-NLNSPL
LKDRDAVRKHIVKDLETFRE---ENGLTLNS-----L--
SL--YFRNYTRWGKEICIYDNFKDWSLVTMNEEEKVKYCKRKTLSSKEN---RDDN
YSQDDNE-------------

>Gcr1a_Vp
M--
--NFYNTNIDYNSINNNSNSNKHTYLNNSHGNNNNAKNNINESGNNKGFFHPLQNSEENQSRNN
TSISQDQLKSNFT----------NKLQSLLYKQDQNFIPLNNTSISNLNTSNITTNFYSITQYILSKYFKVNDINDLNSLKLIDLIVDQTF-----
-------------PYSLTLRKLNDNNSNTPYKYFNTVSRVNDITKCPIFALSIYFIIRWSNPSIMNTVNGSNAMASLITVDN-----------------YNS
IPLLE---SNLISLLDFSNNNLNVPLLS------------SESTNKVSMNLKLARAEAFSPS
DQLIYLVFPWLPQLKSEVLFVDRT-----------------------------------NYKLNSICELFEFIGRTV------VQDLKYLISNPSVLPNIVNFISKLLP-
NLFMNEHFKASN---IINWNDNIFSENNNNNELANGNNIIFDSGNTNINDV-----TSISGTGNNIIPK
QLEEQFSILSKRLTTENVRLNQQISLLKSE----LNSVTNMCNQILQNQKQLLTT--PNN
NQYSNPANNNSNNSDGIIILNKNMLNSNTLGNLVQYIEGMQRPQSQPQQQPQ---
-SQPQQQQQQQSQILPENNPQFNQQSMQPYNNPPNSNYQPQSYSRGNNNLYRNDNSGTNSSNIQKRKLPFPYHTNQIA---------N
NTTYPQSPSTNIAELNVENSYNPPPNKRLRLEDK---QTPSQ-----AALDSLLTGTLPTTKIPLNLTKAIEKGNIINSYI----------------GG
ETPIAANGFEQNVDGANSEDNSPSDSIEIVARSRLRRTMQNFGTNGEQSSMASR-AHNLRSTNVSPVASPNIPKD---KSLSPEKSIE
TGIDET-------NIDINDKDNIDDMD--------------SDEIHRVNSSQYQSDNIHFDRKGQATT---------------------------TSGRPVKHNPNE-----
-----SIKYKLSR-ENKTIWDLYTEWYI--GLNGKPSITQLIKNYGYRRWK----VHDDSHFFPTRRIIMDYI---------ETECDRGVKF----GRF
TNPNQP----REDIRKILVGDLEKFRI---NNGLTLNS-----L--
---------SL--YFRKLSKDNIEICIFENFKTWNVKQMTEDEKNKYCKRQHFSTNQGEN--
----

>Gcr1b_Vp
M--
--EFNDNLKLLLLQKDQVLRENPTN
DENNAELDNFCSVVEFILSHFTNYD-KDILKRLRLTDLTIDTKG------K---NYLQLHIVKANDNNDDDNNTTTTTVPIENFLQISRSN
DITKCPIFALSLYFLFKWHNPK----------DGNITLDN-----------------YTSIPILS--SKDLAAIPTTNKILNKDDTTFK---------------------------
---TDLKLGLELKDYSDIPKDLVNVVFPWLPFLRQNLLLANAM---------------------------------D
YSFYSLLELFEFLGSVL------IQGLLVLVNANSLWLSMLQYIKNFIP-QLFSNETFKNLLKSYTYEL-------------------------------------
--------------KNYDNGTDLNENVFDYDLGKLGLVLKNNKEKSLNAMASEFNTFSNMITLENEKINTEIQDLFAK----VRDMSSRCKEI
LQLQNQILDQ-SETKAID---SNAKESKIDQNQNLIIHLDEKQASHISKERMNSIPIRKLSNSMH
IDIPGASQGA---PIGTELSKDSPTFTPTTMLPSLSQQMS-----------------------------
----
---MFSPVQQGQSHQQK--DVSQLLK
LPLPISN-TPRN-----------SLPYSPSPRDLD------------------LFNPQFLLNEH----------RQSISQSHTD----NRL-----------------KHPLRPPVV
SKHDNPYRVSK---SQVRVSNNYVNKNVP------

>Cdg1_Ct
M--
---SDSEDSSLDVNE------------YTIDDYYKLVKPLLQARN
----SNTSYLNDHKLLRTLVDFNLSHSLSLK-NLAKRELEIGDLIPTKIN----------YIDTVVVKFNAADPTAFAPTTGTPYTTHYAGCFR
NKYVEFCPHFAISAYLFSRFHIPDEYG--SYEFIFSDSSKKIS------------------LENVKLIK--GNNKLSAISYSQQHKSSINALSISGL-NY
KDINLTKLLS----------------------------TH----DIETTEKLASKSIESLSNQVMLNLSGFRNFEEY------NLVRNSIEPPQSLLDKIFPFIDEI
TPEEY-------------------------------------SKEMLQVKQLLIMLRKSL------VQDMVIIKKKYPLNPLSKS--------DLFSSMEFLQF--------------
--LNSVEASNKLDNIINQSMFGPDDDKTESDVDDTNSWPSVIQESETNPKDLNKIIEFQN
TK----IKNLDNLINNVYKQQQERTNELLRSFIEN--QNGIFQSQSEALNKISNSINGLIILMTSQ
NKNALTLANQTLHETSNLIKTIEQT---NLQKGVDNTVELLNNLNQIQAGKTG
ES--SQ
PSTEPPLPSSSSVTAAGSTSATQPTSPTSVVPVDTTP-QSQMAMKPSLSTSTSVPPPAQQPQPPQQQQHNPPPPSLPPTLPPQLPSLL
H--QAQPPQLPIAAP---VSQLQHTMPLQTHHQY------YLNPSNTPPQHRSNPMSPIPQI----ALQQQ------------QLQQQPPPPPPLTA-Q
QIERQRALHRRLSR-QATTLFEMWDDFK---GLEKELRDHDITV----TEWLKVH-GSSERQFRHTRLKIIKFI---------EEEAVRRRTS----
VEY-----------------IKERLHNKMRNRVRPWTLDE-----V---
------QR--MLTANKRIDLDDNS--

>Cdg1_Ca
M--
---SDSEDSE-DLNE------------YTIEDYYKIVRPLLAANN--

--INANYLDNHKLLRTLVDFSLSHSLSLK-NLQKRELEFRNLIPKKIN----------YVDSLIIET-----------KSASGTQYTGCFRNRCIEFCP
HFAIAAYLFSRFHIPDEYG--AYEFTSTESIKKVS-----------------LEHVMLLK--GNSKHQAISYSQQHKSAVNALSVSGL-DYKDVNLG
KLLA----------------------------THKNNEALESMGRLSKNSIRALSRPLMLALAGFDSFEDY------DIIRNSIEPPQQLLDKIFPFLTDIIDE
STR-------------------------------------SKELLQIKELFLMLRRSL-----CQDMVIIKKIYPSNPLSRS--------EIFNSPEFLNF------------------------
--AQSI-DNERLNTIINKSCFIPNEDNI--ICDDNEGSSRVGSSDNTNSKSASK-----ELK----LKTLNK
QMNLVLRQQQEINQA-IYKFTKS---QTEIFQKQNELIQKVNQSLNGVLILLSAQNKTSIPLVQQ
AAQETQNYLSTVGQS---NIERGINNTFELLNVLN--------------------------------------
---S
NSGHSQPQ-------SSVPPFTQQPQP---------------LPPSLSNLLHPETSNIPQAPVSEPPRVIPTQQQAMPLQQQLQYPHPQQFYMQPS
HSPPTHQSAGMMPMAPYGTAPAMGPV------------PVPGQPQPSSSTDASQNPERQRVLHRRLSR-QATTLYEMWDDFK---GLEKAL
EDYGISV---TEWLKLH-GSSERQFRHTRMKIIKFI---------EDEAERRNTN----VED------------------IKQRLHNKMRNRIRPWTLDE-----
V--QR--MLTANKRINLDDNS------------------------------

>Cdg1_Ps
M---
--SDSEDSSIDINE------------YSIRDYYKLLRPLWAAKN--
--INASYLNNHKLLRTLVDFSLTHSLYLK-NLQKRELELGDIIPTKIN-----------YVDALVIKTPNNN-----SLNNTEHAYQYYGCFRNKF
VEFCPHLAIAVYLFSRFHIPDEYG--SLEFMVSDYKNKLS-----------------LEDVKLLK--GNNKLSAISYSQQHKSSINALSLSGL-NYK
DINLNKLLV----------------------------TQ---TLDIQEKLVSLDIDHLPHSVMLSLAGFESFTDY------NIARNSVEPPQELLEQIFPFINKP
NPEE--SLAMTRIRQLLMMLRRTL------CQDMVIIKKKYPSNPVSRN--------PIFSSELFTNF----------------
--CNEEYDQNSSDN--------------------------------KVDLQKIIEIQNSK---IKNLEEQLGNYYSEQ
RVIFSN-LSDFIER---QNEVFQRQSEYMQKIQNSTNGLLVLLSTRNKNMIPLVQQSLSETSEFI
SSINNT---NIKQGLNNSIELLAKLN---
--SNT-HSQQQHI
VSITNNTQSIINQSIVQPPSER--PSSTPFQ--PPPLTP-KQ
IERQTVLRRRLSR-QATTLFEMWDDFK---GLEQELKDHEITV----TEWLKVH-GSSERQFRHTRLKIIKFI--------EDEAARRNCP----V
EF-----------------VKEKLHTKMRNRVRPWTLDE-----V---
----QR--MLTSGKRIDLDD---
>Cdg1_Pg
M---
---AYGQELQHGKKHHAWRHITPPLQRIAHTVCITEMGSSEPYLRKLNAMFLASMTVSRSNSI
NSDESINDI-------SDDSRYHQLLKPLWE------CDTDEVKPHTCLRTLVNFSLGHSFLFP-SGQRREVCIADMRTYELE-----QQGSKS
CPIALIITTNNST---------TSNGPQYAGCLRARDVDKCPVASIAMYLFSRYHIADAYG---NTESGPDFLSAEK-----------------FRNIKLMK
--GSNSSQAMSYSQQHKSAAKAVELAGLKDSKNFSLPKLLT-----------------------------TNRDEDLTRNDRPHLVNLNNLPQQIIYKLAG
FERGCDY------DIGRDRLDPPQEVLKQIFPFIDGDITIE--DPLLLHVRLLFLYLRHVL-----VQDMVLLK
HRYPNNPVLQH-------PFFNTPSFNFY--ASRVNETMALGI--------------------DMY
SSPSPESSPFSLPEPRTHSVSPST---LSQYDAVGRRMVRLQQDHYLR-LQRYMRH---QDWAIR
QQSQQLQKIQEALNGVQILHLARSNGTSGMAEQSLRSIATSLKDLSRC---
SFDSCQGIKELN---
--KTVEQNC
QKLTQFVEERKLMEV------------PTFDNEEFATKRNA-------VLHRRLSR-SAATLYEMWDDFK---SLEKELYANGIST---TEWLKVH-
GSSERQFRHTRMKIIRFV---------EQEASRRNLP----VEQ------------------VKHMLYEKMRNRQRPWTIDE-----V--------------------------------
---QR--QLTAGRRIDL---

>Cry_Cn1
M--LAGIDEPADPDTVQDERQMLLNGVNDLPDFKELRESLGYHGADSSLEHLLQC-------------------------
---N-------QSLASVRLY------LAALVDLWETQRQAGM-------NAF
PSPRTKATNSILNALRRVRNEQSILRCDDKGDDLFYDGI----ATTENMKKLFLHYLH------------RDSVEGLRDLAAQAVGIHGLLR-A
DDQLRITLSSMSLRLFE-DEGPT---PCRGVVFAIREGKT-------THDGQIQYSTLLRNKDVTRCPVSFLVLYLFARFHFSE-EPFINSD
VSFPSLKNRQD-----------------WYHIPLFVSRQSNAVTRLKYDALNKSVRKALQSCNI-HCRASTHTSRKW---------GAQLAEDGGAPE
EDIMRQGRW-CTKVMETV-------YLSKFPLKALRALAGFPK-KKGSY-----YLPRD-MEVPQELIESVFPWVDAAEAELFDPDRF---------
-----------------------QGDKAGRAFIKLMDWFRSVL------IQDAPFIRQLEPDLFVWKH--------PVFSTPTFLAF------------------------------------
-------------------------------EARALAEAQS----------------------------------AEARMSEDARQLIPELSDY----LSTNFTALFKATYNIDTTLTG-LA
ASVASNSQLIQEERRAEKY-------------------------------------DALLNGIGDAFHAMARRQHSGSISSRSNVNAQE-------------------------------
----

--------------------------------SQTTSVLEGQHHGGFNPSASSNSVASQ------------PNSASDSGDLVQLE--------ALVYKMDR-EVGDVLELW
DEYIV--GRNGRLPVREMSQR---NEFKK---NEAEKKMFNRRKPIYEAIR--------DLARGMNMGE----REA-----------------AGLIEEYRI---
KNSMGLNK-----L--SN--VVKEVVKNMIVHQSYRY
RTL---
>CryF1_CGlo
M--AATAIEDARIREAMEYYLLNLAGTVPQNTVRAYKPKQREWKEWCAKNWEPIPEDWATGQ
STWPLGRPLPGDLVDEGKLLLFMKTEVISRAPWRRPRLQKQRKRRLDAAVAVGQ--VVAAKRRRRE------ESETIEVATAPAAAVET
KVEEEDEPLIAAPALE--------LAYNSVRIY------IAAMQRLYDEQKSRHI-------NPAPRPQGIALKALKKSILAVVWARKRKEYSDRLE
GTIKDTY----TK-AQIPRHHEVAWREDS---------GEISCLLRTQVDFLFGNHMLLR-QSNRRPMELPDCFRLELP-NEGQKSKEFPTYA
LVVVMNQGKT-------NQHGRMEYGAALRHRDARCCLVSALAFYLFWRWQVEQVEP------FPTFQCSED-----------------WYDIKVLR
RSAKEATKELSAQTANSWTSRLYAACGI-RTSKISHAPRVA---------AAQNADMDGASEGQIRRAGRWNNGDQLTGC-------YLTSLPF
EFMRSTADFDPDWSGSY-----FLPRDTVKPPFELCARIWPSLDRWKDIYNTGSSS------------------------------METNKATGAFFELLDW
FREVL------LQDAVFLQKLYPRHPLFQD-------PVFQSPQFASF---ALQVENACHAA-
-------------------------------EEDSYVATIDRAIPAVAEK----LRALSSQQTAANLWTERAFVE-----L--
RQQVQRLEKKMEELAKASYTITISPGRSTVTQRVGMPKRGRPRAPGRAPTATAM---

--ATAPRDDST--APVPPSSSN
AAEAERGAVLQPDTATRL-----PL------------PPVAPSVPSAPSAP------SVPRFEVPL-DIRRIPDLWHLWRY--GRAGMPSVESLEARYG-
AAWR----PKSQKSVFCGRKAIVDFIL--------RKSRERDGLQ----SAAEHAPRV-----------IAQMEEL-----CPKWSLDK-----V-------------------
---TK--AIKNGDLERRWPPEA---

>CryF2_CGlo
M--DSDLTYTARLREVVERCLLESQQQTPENTRRAGFASIPE----------------GWPATHVPGRPLPE
DLVDEGKLLLFMADEVVSRAPRTGSRVAQERRKRRETEQAGGI--AKRKKLQGHGKDEVAEWSEDCIVVHTVPADADDADSGDV
ESSF--ESSLK--------LQYNTVRSY------VSAIQKLYDVQRTRGI--------NPAPRPQSVALKAMQKSILRTTWARKRSEYADRGENTIKDS
Y----TP-SQIPVHTSAVWNES---------KQIACALRTQVYFLLGNHMLLR-SSNRRPLELPDCFCLELP-NEGVKNKDNITRAFVVVMNQ

GKT-------NQHGRLEYGACLRHRDPLACLVGALGFWLFYRWHVEK-ES------FPSFSRRKD----------------WYDLKILRRSINEPKDV
LTPQTANDWTRRFYEKSGI-KTTKASHAPRVA--------SSQNADIAGVHEGQIRRAGRWNNGDQMTGC------YLTSLPLEFMRAVAD
FDPEWSGSY-----FIARSTAKPPGRLLARVFPGLDYWKEQHDAPESS------------------------PFAATVQQDKAAGAFLELLAWLREVV--
----LQDAAFLKPLFPNHPIFRD-------PLFQDPEFEQY---AARVRDICSQA-------------
-----------------HEDSHQTAIEKTIPSVAEK----LRSISTQQVAQNALNQRRHEE-----T---VQALRK
LERKLDLLANTRYTVTISPEGKHLEQRVELPRGRRVHRRRRESSLPC---

--SPPPHASSLASTTAAASSSNTEHLLR
TGNEKPVGQHIPTVSPSKA------------PGVAVVGPEDDESP--------PRFRFPQ-SVRTVMELWRLWRH--GLPPMPSIQSLEERWG-AR
WR----ARGDRQYFSIRRRIFDEIV-------RRSQARDQPE----EVS----------------AREMDIERG-----KISLDK-----F-----------------------------
--SK--ALRAQRGQGTG--

>MarCry1_FO
M--AADDSYTAADERARAARRAGLQAKKDEQPANTARSYAAKQREWKAHGSLYSWPDG----------
-------ELVTPDKLAAWLKEDILLRRVAPPQKKPRARGKGKGKGKAVQLRRQLEQEQLEAAALAEAESLAEALEVPLAEAAELLADD
REGYVPPTALAPAAADLAEGSLLTRGTIDAY------IAAVIELWRLQVAHGN-------ANTENPRGAAVRGFLEQRGRQRGKHDRASFK
DRGTDGIQAGY----SP-DEWLRVQDLLLSGA---------AYMPQNLRTRVDLLFGHYYLLR-GENRRKMELADLSLLDYPSSEGPT----PC
GCLVTLLRDGKL-------NKTAKKEFMGALRHKDPLFCTQGALAQLFFWRWHVAG-EP------SPSFRRRQD----------------WYRIKVLV
--GRDREQELSYPTQLQETWRIFGAAGL-MASKKTHLPRRV--------GAQDAETHGTSLAQISQAGRW-NQSVLCQA------YLTRLPRQ
FMRIVAGFSA-SPGDY-----FLARAAHEPPYVLQKQLWPWIEEWEPRFEARARR---------------------QCWAEGGLDDDDLAADGFLKL
MRRLRIVL------LQDLAVLQPRYPSLPFFAY-------APFNGPEWDEF---AVAVRSDAV
G-------------------------------ATEPLSLLVQRALPELSGV---LESTREAVLQNSQRLAIRL---
-EARLEGIQGGLDALLQGKVPVTFTGHFGAGP---

---AVSLAPAPAPSTAPTLNFNTA
PA------------PAPEPPVPG------------MPVVAALA-KVFTVRDVWKEWEE--GIAGQPAVRVLEETWG-SRWRP---GNGIRVQFCRRKVIW
DEL--------LARTASGKSE----EEA----------------VAELELLRA-----GRSLNR-----L---
-----------------------------------VD--ELKQRRRRGQGQGRIRVQVGTPVPDDPGPGPRPTRGQGHRGRWARLGRRRTAPRRR-------------
----

>CryF1_CI
M--ATPTLNSQSALMVEMEARVRHALDSALDARPKKTRMQYESRQEEWRHFCRTKGFQNG-------
----------ELVTEQKLLWFLSECVVGC---PAKRSCHSRDRTNENGEPVVQ---T-------LGQPSIKAY---
---KTAIVNLWSYQRSCRT-------NSYPHPVGQAVRALLETHSRQEHERKKAEFVDCGTGTLLDGY---HE-KDIVQLVEYCWQGW----S
ESKKGQSVEPHLRTAVDFLMGHSMLLR-GESRRTAQLADLFTLELT-NEGPT---PCFPMVLIMGNGKT-------NQMGWIEYATVMH
HRNLLLCTMAQTAFYLFYRWDIVR-EP------PPQFHNHQD----------------WYQLHLIK--RDDVRKPLSYKTQLDWIRCIYSGTGL-SG
LKKTHAGRAA--------GARHAEQVGVSEGQICRAGRW-NSDALSQC------YLTNIPRKFVQAMAGFDSRTPGNF----YLPRARVPVPE
SLERAVWPWVDDWMRWFESYDAQDPNSCLQGPELRRFSDQSGPWDQPRGPSAVQLDRDDLAAQEFLRLLHHLHTVL-----LQD
SVILQPLFPSHPLWTS-------PVFMREDYRQF---AEAVQMANTH----------------------
---------KEELYEMQLQQTVPMVADQ----LRIVQQDLGVACGHLHTAL--ETGLQKINNQ
LEALTSGQVSFLVRAFPAGQRMM---

---TAAAAFEDKDEERDSGSTASREAQAPACSLEIAPA--
-----------------SASASV-------PATYQLSR-TITTVPDLWQEWTV--GLGGGPSVQSLDALYG-AKWRP---GGTERMFYSRRKVIIDYI------
--NRQRQGSASG----SAA----------------VEELELVRQ---RGKLSLNR-----L--
--------------------------SR--MLATGSGHCVEVQTMFRCRTQGLYD---

>CryF1_CP
M--ATPTLNSQSALMVEMEARIQHALDSALDARPKKTRMQYESRQEEWRHFCRTKGFQDG--------
---------ELVTEQKLLWFLSECVVGR---PAKRSCHSRDRTNENGEPVVQ---T-------LGQPSIKAY----
--KAAIVNLWSYQQSCRT-------NPHPHPVGQAIQALLETHSRQEHERKKAEFVDHSTGTLLDGY---HE-KDIVRLVEYCWQGW----S
ESKKGQSVEPHLRTAVDFLMGHSMLLR-SESRRTAQLADLFTLELT-NEGPT---PCFPMVLIMGNGKT-------NQMGRIEYATVMRH
RNPLLCTMAQTAFYLFYRWDIVQ-EP-------PPQFHNRQD----------------WYQLHLIK--RDDVRKPLSYETQLDWIRRIYSGTGL-SGL
KKTHAGRAA--------GARHAEQVGVSEGQIRRAGRW-NSDALSQC------YLTNIPRKFVQAMAGFDSRTPGNF----YLPRARVPVPES
LERAVWPWVDDWMRWFESYDAQDPNSCLQGPELRRFSDQSGSWDQPRGPSAVRLDRDDLAAQGFLRLLHHLRTVL-----LQDSV
ILQPLFPGHPLWTS-------PVFMREDYHQF---AEAVRVANTH--------------------------
-----KEEPYEMQLQQTVPMVADQ----LRIVQQDLGVACGHLHTAL--ETGLQKINNQLE
ALTSGQVSFLVRAFPAGQRMM---

--TAAAAFEDKDEERDSGSTASREAQAPACSLEIAPA-----
-------------SASASV-------PATYQLSR-TITTVPDLWQEWTV--GLGGGPSVQSLDALYG-AKWRP---GGTERMFYSRRKVIIDYI--------N
RQRQGSASG----SAA----------------VEELELVRQ---RGKLSLNR-----L---
----------------------SR--MLRVKKKLP--

>CryF1_MC
M--FLQEEVMYR---EIRPSRYKKSRT
NIEGESVKQ---T-------LSEASIKAY------IAALVDLWGVQVSMRT-------NTHASPRGALVKGLIRT
RRQQESKRKREQYEDRGLNSLQDGY----TS-AEFVRLIRFCWSSGYNVS-----GQLEPFLRTAADLLLAHTLLLR-GEVRRKVQMADL
FTLTLD-NEGPT---PCHAMVVLIDNGKT-------NQDGRREYGTAVRHKNPLLCTMASIGFYFFMRWNIMG-ET------PPNFQRREL---
-------------WYDLLVIK--GQSNVKPIAYETQLQWSNRVYKGIGL-GIKKKTHAGRSQ--------GAKFAELAGVNEGQIRRAGRW-NSDS
LTTS------YLTNIPRKFVRAMAGFQPVETGAY----YLPRTKIEPPASLKQALWPWVDEWLLWQASYTGSGSEDVQLPPESVRF-------
-----DSGKVDHGDLASQGFLYLLDYLRTVI-----LQDSVLFMQEFPGHPIWSH-------SLFARDDYLCF--------------------------------------
-----------------------SQLVRDSLID-------------------------------VEEPQDVQLRRTVPAIATR---LQTIEQSIVQTLKVSEERN----------------
--VGFLQTIDRNIKDIVDGTVSFTVRANAPGSPLS--

--AAATATAT
AASTPYATPRCMHGAIPHPSLSPSCSPH---------------PPSSSAVAAAPHTG----PPPLSMGFSQSR-TIGTVMDLWREWAE--GLGGRP
SVRSMEEAYG-PRWRR-H-RPGESMMFSRRKVVVDEIV-------RRQRVQKKSL----SVV----------------IEELETNMD---WCVASLASPP
PSFLLLFLLLLSLFHVHVRIEYQDRFQSISTPILFLSSFFSSFEMPFNNNTAGDGMALVLYDRQIARMTDTPGILRVGGQELAPYVH
NDLGRAVLSLPGLHVRYVPYSIDARQILSHRASYINGLLVQSRGRPEVHACTDCRGRTGLRPFTECVRLPGHFGGACGNCKWRDR
ASHCSVRDEIQQARTEVIVLDDDSGDDRQVIDLTGDGPVRRPRARITGGGNGSSASRAIVLL--------------------
>CryF1_TS
MQCGVTEIDALSRIAAGLIVRVDYAMDRLLSLLPRTAPLQIQVPDIATTEIEPPRPEDIAIQKALDEVLEYHRVHRPKNTTKNYEPK
QKEWKAWCKKMGFKEG-----------GRYLPGDYVDEGKLLLFIKEEVASRPPRRGQRLKAERKRKRT-AAAEVLSEGPPSKRKREK---

--------ISVPSMAFEELPVE----SDDDEACSELV--------LMYNTVRSY-------CSAINELWAHQTSLGL--------HNAARPQRVAMTALKTSIA
RGQHQRRRDEFTDRGLATIRDGY----VA-SQIPDLTRKVWAQCLGQ-------NQIEQQFRTQLCFLFGNSMLLR-LSNRLPMELPDLFS
MPLP-NEGPKG---KGWCLVTVMDQGKT--------NQHGRLEYGAALRHRDHQSCLIGALATYFFWRWHCSG-ES-------FPCFRTSQD---
---------------WYNIKVLKRDNNHLTEPLSDSTAASWTRRLYSEAGI-KSSKVTHAGRVS---------GARLAELNGVSEDQIRRGGRW-NAD
QMTGC-------YLTTLPRSFMRGIADFDPDWSSSY-----YLPRETVCPPPALLKRVWPDLDRWQAAHLERTDA----------------------------TE
RVEPNIAAGGFLELLQRLRTVF------LQDSVLWRTEFPSHPIFRD--------PLFQMAEYKAF---
----------ELDIHMAVTTAM---------------------------------EEDPHSIAIQKAIPAVNDR----LRTMTAAIQTGQVTHSQALRS-LEDLM------------
--VSRIDQLTATINDFIGGTFTCQFVPRGQIVPP---

------------------------------PITGAAFGPTAPIQ--------LPVTALEKASQAPQYQLWKEWTV--GLNGQLSIERLDELYG-SGWRSGPESSAER
QFYSRRKTLINEIR--------RLATVEDASLGDPCQTV-----------------VAKLEEERI---RAGASLSKISNPKI---
----

>CryF2_TS
M-------------------------------------ESISNGLLAEQDRQLELHAKIQLHSQKVAHRIQKNRPAATTRQYDSRQKEFIDFCTKEGFPDG------
-----------QVVTEKKLVYFLDHYVINR---PIRPSRYLRNRTDSQGAAVVQ---T-------LGLPSVKAY---
----TSAIVDLWRFQQSLGT--------NPYPNPRGHLVGAMIKNHQFDETQRKRTQFTDRGFNTLQDGY----TS-ENIRAIVRYCWAGWLS
DQTRGRKPQAQEAYLRTTVDFLFGHNMLLR-GEDRRHLELADLFTLRM--DEGPT----PCWPMILMKLNGKT--------NQFGRLEYM
GVVRHKDPLLCTICHTAFYLFHXWEIMH-EP-------VPQFYQRQQ-----------------WYKXVLFK--GSDSEHSFSYETQLKWINQVFQSI
GL-NSKKKTHSGRSS---------GARHAELQGVDENSIRRAGHW-NQDSMSNC-------YLSELPRPFIRTLAGFKPTDQGNY-----YLPRAAI
EPPETLVRALWPWIDQWLAWFSPSESN---------PVELSKLDLPPLPLLVQQGVEKCDQDDLAAQSFLKLLSSFRTVI------IQXAVFLQ
QEFPGHSMWTH--------PLFQRSDFQSF---SQQIIDLVRT---------------------------------S
ETPHEIKLRQTIPLVANR----ITTIGENLEHIIQLNHQQT---QDSIRAIQSQMDQLFSGEV
TFTARLTGSDEKPS--

--TPGNTTSQSTEQNTAHTVVQSLQVTPNPIQD----------------HPPGIH
LPNEPSGS--------PPFYRMSR-TIQTVRELWEEWHV--GIHGNPSIQSLEDSYG-CRWRS---DNKERVFFSRRKVIIDWI---------QARVS
KGILL----ADA------------------IDEIELMRR---NSQRTLYQ-----L--
-------------QA--LLKKGV---

>CryF1_NF
MESKVTETDALLRSVAGLVVSVDRAMDRLLSLLPRTAP--IQAPEIATPETEAPRPEDIPIRRALDEVLEYHRAHRPKNITKNYEPKQ
KEWKAWCRNMGFKAG------------GRYLPGDYVDDGKLLLFIKDE----------RLKAERERKRTAAAAELTSRAPPSKRKRGK-----------TS
APSAASGELPVEGDDADDDDKACSELV--------LMYNTVRSY-------CSAINELWAHQTSSGL--------YNAARPQRVAMTALKTSIAQGQ
HQRRRHEFTDRGLATIRDGY----VA-SRIPDLTRKVWGQCLGQ-------NQVERQFRTQLCFLFGNSMLLR-LSNCLPMELPDLFSMPL
P-NERPKG---RGWCLVTVLDQ---VLKRDNNHLTEPLSDTTAASWT
QRLYSEAGI-KSPKVTHAGRVS---------GARLAELNGVSEDQIRRGGRW-NADQMTGC-------YLTTLPRAFMRGIADFDPDWSSSY-----
YLPREAVCPPPALLNRVWPDLDRWQAAHLERADV----------------------------TERVEPNLAAGGFLRLLQRLRAAF------LQDSVLWRM
EFPGHPIFRD--------PLFETAEYKAF---ELDVRGAITTAV-------------------------------EE
DPHSIAIQKAVPAVNDR----LCTMTAVIQSGQV--

--PQYHMSR-TIRTIPDLWQEWTV--RLKGQPSIE
RLDDLYG-SGWRSGPEKSAERQFYSPRKTLITEIR--------RLAAAEDPSLGDPYQKV-----------------VA--
----

>CryF1_RO
MSH----------------------------DSNAGPSTRDNDIALPDAEHYEDMIRARLAMDKNTQMVIAENQTYRPKNTTAAYKSKQREWFEWCAN
KEKVAD----G-------------TIVYDAKLAFFLKDYVLTR---------GNKFKKNADGSPA---P--------LGRE
SVLAY-------VEA-----------GF--------NKHTMARGPIVKRFLDTHTKKEARRKRTEYEDRGKNTLNDGY----TD-QELLRINQYFLI---------
---QNNIFSLRNKVCFSMSHAMLMR-SETALGTQLPDLFIMELK-NQGPS----TCFAIVATITFGKT--------NKDGKIQYGSALRHRDVE
VCPHGAFAQYFFSLFHHQN-LP-------FPNFSTRRD-----------------WY-------------------------------CGV-HSSKLTHINRKS---------AINMVA
NEGVSGDQQRQVGRW-GSDRMVGC-------YLSGLPVDAIKVLAGFTT-RKGDY-----FINRGSIEPSEELRKMVFPWIEYWREKFYGK
EVE------------------------------------DDIAGPNFLDLMDYLRTVF------LQDSVVLKGKYPGSFIWSH--------SIFDTDIYKDY-------------------------
--EERLSSAIAAND---------------------------------EKFKMSQHLEVLLPEVAAA----MKTGFDSMNAMLNIV
QSQNQL-----T--LNAVKQLEAENRTMLADSFL--

--------------QISNMLRQGDENTQIQQTSTILPSSSV-------------PLSSIPSPSAANSR-------GLPSFKMSR-SLISVTDVWREYSV--GLAGK
PSIKSMEEQYG-ADWRK-VDNRTESRFFTRRLPLYKKIE--------SLSNERGISC----EEA------------------AQFLEFRRV---SDKLSINQ-----
M---CK--LVADNQI---

>CryF1_PI
M--EPPGLQVELEESAHATLDRCREARPANTIRAYAPKQREFKAWCERKGFHET-----------------T
RYQVTAAKMHLFLQEEVVDRQVRTKGSAR--K--------VSVATVEMY-------VNAVSDL
YSDQQSRGA--------NSHPHPRNSLIKGLLTSLKRESHAKNKREYADRGVGSLLDGY----CTTNDLVSISRYYMN------------LNTGSDLR
NRMSHFLCHACLLR-GESARNLDLPDLFSVILE-HEGFT----ECRALVMIMEQGKT--------NQFGRREFGSCIRHRNVEVCPVGALAL
YLFWRWSVQK-EA-------VPDFLVPER------------------WYDIKLLK-SNKDITTPMTYRAHYDATVKAFSALGM-RSKAKTHAARGS------
---GARMAELAGATESQIRRLGRW-NASAMEGC-------YLSALPREAMRSLAGFPP-DRRTF-----FLDRAAIDPPECLLRDVFPFVETYM
AAYMQQSAP------------------------------------HVATGGFLELLLYLRVVL------LQDAVQLRDLHPTHKIWMH--------PPFNSTGFDSF----------
--ALELKAKMLT-----------------------------------EKSPQTTHLHEVVPDLMDH----LKQQQQQTI
AHIDNKINEI---STSINHIATAFSRLTSGSSAVRLTVDWGSAQ-------------------------------------
----

---SSTSTPVAASQHAP-------LPPTYKLVR-SIKTVQQVWQEWTT--GIHGGPAVRD
LEERYG-SNWRN---SPAEKRFFFRRKRIIDRVT--------LVAQQQHVSE----AQA------------------VCILEGQRT---QSQLTLNA-----L-----------
--SE--SLKGRQ--

>CryF1_PS
M--QVALKESVQRTYEKATTLRPKNTQRAYSSRQQEFLDWCSEKGAAFN--------------NLTR
FTVTGEKLHLFLQERVIGR---------TKRHKSGSTPSDRIE--T--------VGRSTVNSY-------VAAMVDL
WKQQSRAKV---------NSNPSPRDEAVTTLLKLTEYEEDERKRKIFEDRGADTLLDGY----TTIDQVKEIARYFWTPA----------RDSGKNL

RNLVAFLLSHYALMR-GESARMMELADLHSIVLE-NEGYT----PCRALVMVMRQGKT--------NQVGRIEVGACMRNKHVEICPHGIL
GFYLFWRWHVDG-EA-------FPDFTSSER------------------WYPIKLLK-TGKHPTKPMSYKVHNAAITAALKHVGV-HSKAKTHVGRGS-
--------GSRMADLGGASESQIRRLGRW-NGQSMEKC-------YLTSLPRQAMRTLAGFEP-SIGSF-----FVARASVDPPLALQSMVFPQVE
VWQQAIASGTAE-----------------------------------QSLAAGGFLELLQYLRKVV------LQDAVFLQKSAPTHPIWQH--------SIFQSREFTEF-----
---KSEVMQAVDA------------------------------------MEDPTEQRLQKAVPVLNAK----IDGLHQD
FKSSLAQVCSTMRT-----V--QGSLSDVMRVLAPLQGGTAVVQVQLLSGGRE-----------------------
----

--GDGDTAHV-------------VPSASPIAIDVQSD--------RTKYKLRR-GLATVPDLWKEWSV--
GLDGARAVRDLEDQFG-TKWC----SADERRFFNRGRPIYSLVAAVADDILTSARVSDVDAT----NLA------------------VQVLEEYRR---TH
NKSLNW-----I---AK--HAGEIKDAVQAIAVSRCSVST
TSISDPSPND---
>CryF1_SaPa
M--LRPSAADVQEAALAFAMQRSAMETYEMAADKRPKATKAAYSAKAQEYVDWFKAKPGNAN------
----------KLPLVDAQTLHYFIKDKVIGRTARPKTKKDTGAGSTKESTK--V--------IGYATVKQY--
-----VNAIVDLYQEQVRQRA--------NTNPHPRNNLVKTLLKQVSLAEDERKRANYEDRGAGTLIDGY----TTQEQLSQIAKHYWTPA---
-------SFFGVRLRDWLAFALSHYYLLR-GETARMLELADLQSVQLE-NEGCD------------------------------GVLPFLALARRRRA-----------------
-------------LPRDDHECR------------------LVRVQAAQ-ERQEPEKEMTYSTHHKAVSDALTATGL-STRAKTHVARGS---------GARMAEMG
GASEAQIRRLGHW-NNQAMEGCYLTKLPRYLTKLPREAMRVMAGFSP-DPRLY-----YLERGQVEPDQELQALVFPDAANWLAKLN
DGKCE-----------------------------------ATIAARGFLELLMHLRVVL------LQDSVLLKKLYPAHPMWSA--------PLFSHPLYTAF-------------------
---EIKMEHLLA---LTTSMNLVTSG----LEDVRAVVNNL-----------------
---RTDVAAQGQVVSNLASGRFVMRLEMEPDGAPR--

---------QSPPRATQKPSAKPSTQPTTTASAMSN-------------NPSTTRVAIGNQMS-----------------RVQTLPELWREWFH--GLGDRPSVVSI
EQTPS-ARWR----NDSARRFYNRRRVVIRAVQ--------AYASRTRMPI----DAA------------------VARLEERRL---QQNRTLDW-----I-------------
---AK--HDNTFIHDD--
