# Supplementary material for: Crypton transposons: identification of new diverse families and ancient domestication events
Source: Mob DNA. 2011 Oct 19;2:12. doi: 10.1186/1759-8753-2-12 (PMC3212892; doi:10.1186/1759-8753-2-12)
Supplement: Additional file 4 — PDF file showing alignment of Cryptons and Crypton-derived genes in animals in fasta format. [file 1759-8753-2-12-S4.PDF]

Additional file 4. Alignment of *Cryptons* and *Crypton*-derived genes in animals in fasta format.

```
>WOC_Dm
MFFNTKHFNLTTVEEH---------MQLSFSHIMKHWKRSSQN---SKVPG------SRNVLLRFY----------PPQAGLDAN---------------------
PRKKKVYEQQENEENPLRCPVR-----LYEFYL-SKCPESV----------------------------------------------------------------------KTRNDVFYLQ
PERSC----------VPDSPVWYSTQA---------LGQDAL----QRMLHRVK----------------------------------------------------------------MVKEINIA
----------------LLTT
>WOC_Tc
MFFNTKHFNLTSVQEH---------MQLSFSHIMKHWKRNPNQPGVTRVPG------SRNVLLRFY----------PPQTAIQNN-------------------
--TRKKKVYEQQENDENPLRCPVK-----LYEFYL-SKCPESV----------------------------------------------------------------------KTRNDVFYL
QPERSC----------VPDSPVWYSTTA---------LPQEAL----EKMLHRVK----------------------------------------------------------------MVKEINV
A----------------LLTS
>WOC_Sm
LYYNTKHIGLRLGTVH---------RQLAFSQFQIS--------------------ETAVLCHLP----------------------------------GNMFGESDDSITTLSLD
ANPQHPQRCPVK-----LYKAYF-ARCPPEL----------------------------------------------------------------------LDSDSVFYLNPLY------------PP
QSDLWFSTNP---------VRPTEL----QVILNRIK----------------------------------------------------------------MVKEIQEA---------------FMNSQ
>WOC_Acal
IYFHTKHFILKTAQEH---------MSLSFAQILKHLKKGVPTKGSTAS--------SKNVALRYY--------------------CLANGKKDGLAARKS----
---KEGVPVYEVTENFDNPLRCPVK-----LYEFFL-SKCPESI----------------------------------------------------------------------KNRHDIFYP
VPERSC----------VPDSPVWYSTQT---------MDLHTM----DKMLTRIL----------------------------------------------------------------LVREIQES
---------------HLHAQ
>WOC_Sp
FFFNCRNLRMRSVAEH---------SKLAFFRMQKLVKKAPVPS------------GKVSLLRYF----------PPKKKNEAE--AGKKGAVLNIGXR
KKREY---DYDAETLDQSENTENPLRCPVK-----LYEFYL-SKCPESI----------------------------------------------------------------------KMR
NEIFYLLPEHSC----------IPDSPVWYSATT---------LPDEEV----ERMLRRYG----------------------------------------------------------------MI
QEVQAG---------------WQQKM
>ZMYM2_Hs
FYFNTKYFGLKTVEQH---------LRLSFGTVFRHWKKNPLTM------------ENKACLRYQ----------------VSSLCGTDNEDKITTGKRKH
-------EDDEPVFEQIENTANPSRCPVK-----MFECYL-SKSPQNL----------------------------------------------------------------------NQRMDV
FYLQPECSS----------STDSPVWYTSTS---------LDRNTL----ENMLVRVL----------------------------------------------------------------LVKDI
YDK---------------DNYEL
>ZMYM2_Xt
LYFNTKYFNLKTVEQH---------LRLSFGNVFRQWKSNPLTN------------ESKACLQYQ----------------VTSLCESDSEDKIAPGKRKH--
-----EDEEPVFEQLENTADPSRCPVN-----IFDYYL-SKSPQNL----------------------------------------------------------------------NQRMDVF
YLKPESSA----------STDSTIWYTSSS---------LERNIL----ENMLIRVL----------------------------------------------------------------LVKDIYD
K---------------KSYEL
>ZMYM2_Dr
FYLNTKYFGLRTPEQH---------LRLSFANVYGPSSSKPHSH------------ETTVCIRIP-------------------SISQEQHEQSGSKRRRK------D
YCDSNFDSDSGSGGSAHCPVKKHECRLYELYL-SKCPESV----------------------------------------------------------------------RQRTDFFY
MKPEVSA----------SSDSPLWFSSTP---------LEKSVL----SRLLTRAL----------------------------------------------------------------LVREVYK
D---------------NQPEE
>ZMYM3_Hs
MFFNTKFFGLQTAEEH---------MQLSFTNVVRQSRKCTTPRGT----------TKVVSIRYY----------APVRQRKGR-------DTGPGKRKR--
-----EDEAPILEQRENRMNPLRCPVK-----FYEFYL-SKCPESL----------------------------------------------------------------------RTRNDVFY
LQPERSC----------IAESPLWYSVIP---------MDRSML----ESMLNRIL----------------------------------------------------------------AVREIYEE
---------------LGRPG
>ZMYM3_Gg
MFFNTKFFGLQTAEEH---------MQLSFTNVVRQSRKCTTAREH----------LFVPFLAF-----------PVMSQLXSYSVPPPFADGSTGKR
KR-------EEEMPMLEQRENKMNPLRCPVK-----FYEFYL-SKCPESM----------------------------------------------------------------------RNR
SDIFYLQPERSC----------IAESPLWYSVIP---------MDRSML----ESMLNRIL----------------------------------------------------------------VVR
EIYEE---------------HSRLS
>ZMYM4_Hs
LFFNTKYFQLKNVTEH---------LKLSFAHVMRRTRTLKYS-------------TKMTYLRFF----------PPLQKQESE-----PDKLTVGKRKRN
ED----DEVPVGVEMAENTDNPLRCPVR-----LYEFYL-SKCSESV----------------------------------------------------------------------KQRNDV
FYLQPERSC----------VPNSPMWYSTFP---------IDPGTL----DTMLTRIL----------------------------------------------------------------MVREV
HEE---------------LAKAK
>ZMYM4_Xt
LFFTTKYFQLKTVSEH---------QQLSFAYVMRRTKTMKYN-------------TKTTYLRFM----------PPCHKSDTE-----HEKPSVGKRKRV
ED----EDGQFVMEMAENTDNPLRCPVR-----LYEFYL-SKCSESV----------------------------------------------------------------------KQRND
VFYLQPERSC----------VPNSPMWFSTMP---------IDPGTL----DSMLTRIL----------------------------------------------------------------MVR
EVHEE---------------LAKAK
>ZMYM4a_Dr
IYFFTKYFNYRTAEQH---------RLLSFGHIVRCSRSKGN--------------TKVACLRFY----------PPKDEADG---------VPAKRRKEEGE---
EEDETVYEIKENSDNPLRCPVR-----LYEFYL-SKCSPSV----------------------------------------------------------------------RQRTTDFYLSP
ERSC----------VPNSPMWFSISA---------LSDEAL----NSMLTRIL----------------------------------------------------------------TVRELHLD-----
-----------TEKTP
>ZMYM4b_Dr
LFFGSKLLNLKTVEEH---------RRLAFSNVSHCSKTTKS--------------GLTSYLRFR----------LPNKEEEKE------KKSPAGKRKRDEE
----SVEEEFLEMPENVENPLRCPVR-----LYEFYL-SKCSNSV----------------------------------------------------------------------KQRPDLFY
LQPEASC----------HPSSPVWYSAQP---------LDSTMM----ESMLTRIL----------------------------------------------------------------AIQDIHL
D---------------SKHQQ
>QRICH1_Hs
MFFNTKYFLLKTVDQH---------MKLAFSKVLRQTKKNPSNPK-----------DKSTSIRYL---------------------------KALGIHQTGQK----V
TDDMYAEQTENPENPLRCPIK-----LYDFYL-FKCPQSV----------------------------------------------------------------------KGRNDTFYLTP
EPVV----------APNSPIWYSVQP---------ISREQM----GQMLTRIL----------------------------------------------------------------VIREIQEA-----
----------IAVAN
```

```
>QRICH1_Xt
MFFNTKYFLLKTVDQH---------NKLAFSKVLRQTKKNPSNPK------------DKSTSIRYL----------------------------KALGIHQAGQQKGF
SITDDMYAEQTENPENPLRCPIK-----LYDFYL-FKCPQSV-------------------------------------------------------------------------KGRNDTFYLT
PEPVV----------APNSPIWYSTQP---------ISREQM----EQMLTRIL-------------------------------------------------------------------LIREIQEA-----
-----------IVVAS
>QRICH1_Dr
MYFNTKYFHLTTVEQH---------MKVAFSKVLRHTKKNPTNPK------------DKSTSIRYL----------------------------KGVGPHHVGQK----V
TDDMYAEQAEDPENPLRCPIK-----LYDFYL-FKCPQTA--------------------------------------------------------------------------KGRNDTYYLTP
EPVV----------APNSPIWYSTQP---------ITSEQL----EHMLTRIM-------------------------------------------------------------------MVREIQEI-----
----------ISITQ
>KCTD1_Hs
WFETCMYFCTRGRENQ---------RELEEDSFGLAMDE------------------DGRKFVYFKSLGPYHKSRSSSWSKKRA----------------ES------
--SDEENLPRMYETGT--EFCPYA-----SFVKYL-SKRNPLC-----------------------------------------------------------------------------KAFFQRPR
DHC-------SEGDV---TWYENKA---------IGKNLL----GTRMQMLS-------K------AAKL------------------SKTYTNHCIGAVSIATLNSIAGIG
TKLGSPAPQG-CYAEALNG--------------------A
>KCTD1_Xt
WFETCMYFCTRGRENQ---------RELEEDSFGLAMDE------------------DGRRFVYFKALGPYHKSRSSSWGKKRS----------CPGGSTM
E-------SDEENLPRMYETGT--EFCPYA-----SFVKYL-SKRNPLC-----------------------------------------------------------------------------KAFFQ
RPRDHG-------SESDV---TWYENKA---------IGKNLL----GTRMQMLS-------K------AAKL------------------SKTYTNHCIGAVSVATLNSIA
GIGTKLGPPY-----LTDSLNG--------------------S
>KCTD1_Dr
WFETCMYFCTRGRENQ---------RELEEDSFGLAVDE------------------DGRKFVYFKALGPYHKSRSASWTKKRS-----------------D-------
TDDDNLPRMYETAT--EFCPYA-----SFVKYL-SKRNPLC-----------------------------------------------------------------------------KAFFQRPRD
HC-------SDTDT---TWYENKA---------IGKNLL----GTRMQMLS-------R------AAKL------------------SKTYTNHCIGAVSIATLNGIAGIGSK
LAPLR----ASPETVNG--------------------A
>1958a_Hs
VKYNSQYLNMRTLQEH---------ADLMYGDIELLKDP------------------QNQPYFART------------------------DSVKRESRSGS-------TRVC
HGKIYHEHSRGHKQCPYC-----LLYKMYIHRPPTQ--------------------------------------------------------------------------MEAKSPFYLTARK
EA----------TDMGSVWYEEQR---------MGLRSL----RGIVPNLA-------K------KVKLEN----------------CENFTFVSFTQVSRRLGS-----------
----------------------------HSCCQ
>1958a_Xt
VKYNSQ-LNMRTLQEH---------ADLMYGDIELLKDA------------------NNRPFFART------------------------DSVKRDKQANP-------NKMC
YGQIYHEHSKGPRRCPYC-----LLYKMYTYRPPTQ--------------------------------------------------------------------------IEVHSPFYLTARKE
V----------TGNEKIWYEEQR---------MGLRSL----RGVVPKLA-------K------RVKLDQ----------------CENYTFVSFTQTLKKFG---------------
-------------------------PLHNY
>1958a_Cm
VKFNSQYFNMRTLQEH---------VDLTYGDIELMKDA------------------ENRPFFART------------------------DMVKRENKVCG-------NKLF
FGQIYHEHSEGLKRCPYC-----LLYKMYAHRPLSH--------------------------------------------------------------------------REPKSPFYLVARKD
F----------SQLDSVWFEEQR---------MGLRSL----RAVVPKLA-------K------KVRLEQ----------------SENFTFVSFTQAS-------------LLKPG
KF------------------------LSVNS
>1958b_Hs
WLNNTKAFGHCTGFHG---------STLKWGDIRLRVTE------------------TGLEYLEWM-----GQDTGDLNAKTKRGGTDS--------------------
-------RVYATQHAPQTCPVQ-----DYKEYA-QRRPPAM--------------------------------------------------------------------------RYEDAPFYLSIKPV
V-------NLAAL---HWYNCQA---------LGKNKL----AKMVKTMC-------E------KGNI------------------PGRKTNFSVYQSCSTL----SEAQSNQ
LVLICNN-LSQQAAQ-----------------SVAGH
>1958b_Xt
WLNNTKAFGHCTGFHG---------STLKWGDVRLRVLE------------------TGLEYLEWM-----GQESPD--VKIKRAGTEC----------------------
-----RVYATPHSPQTCPVQ-----DYKEYA-QRRPPAM--------------------------------------------------------------------------RYEDAPFYLSIKPVV-
-------NLAAL---HWYNCQA---------LGKNKL----AKMVKTMC-------E------KGNI------------------PGRKTNFSVYQSCSTL----SEAQSNQL
VLICNN-LSQQAAQ-----------------SMASH
>1958b_Dr
WLNNTKAFGHCTGFHS---------STLKWGDVRLLTTE------------------TGSECLEWT-----YQDLSDPNARNRRSTTEC---------------------
------RIYATPHAPQTCPVE-----DYKHFA-QRRPQAM--------------------------------------------------------------------------LYEDAPFYLSIKPVV-
-------NLAAL---HWYNCQA---------LGKNKL----AKMVKTMC-------E------KGNI------------------PGRKTNFSVYQSCSSL----SEAQSNQL
VLICND-LRQQAVH-----------------SGSSH
>1958L_Oa
ILNIVRGFGALQCFQN---------VTVTWGQIILKKR-------------------GNLEILEWH-------------------------------GDEN-------PSPDKKNYIT
EKPESPEDCPIL-----DYKEYS-RKRPLDM--------------------------------------------------------------------------LSCNSPFYLAPKPVC-------SV
CDQ---IWFSKKS---------LLKTKV----KKLLKTLL-------Q---------------------------------HIKRGLS-----------------------------------------------K
>1958L_Gg
FVNIIRGFGASTHNKG---------QNLYWGQLVLRRDV------------------AGLEYLEWK--------HDLNADGAVGESVP---------------------------
HLFARPNSPENCPVQ-----DYKKYA-EKRPGDM--------------------------------------------------------------------------LHDYDPLYLSPRHMY---
-----SVWDQ---VWYSRKS---------LTTAKI----GNMLKVIL-------R------QVK---------------------------------------------------ASEKKLR-----------
----------K
>ATFIP7_Tg
WFDLQLHFAKRGREIL---------RDLAPDAFVVEKDK------------------NGRRYAMFR--------YPGKGKNGE--------------------------DPQK
MGKMYDMPGD-PNCPVF-----SLELYL-SKLPPEP-----------------------------------------------------------------------------PAFYLHPLKLT-----
--SEQMQEQPVWYKREP---------MGVNYL----GTMMPRIS-------V------AARL------------------SQRYTNHSLRTTTIQLLCE-AGLGPREI
MAVTGH-RSESAIRH----------------YWGSA
>AAVX010680
WFDLQLHFAKRGREIL---------RDLPPDAFVIKRDP------------------NGRRYAMLK--------YTGKGRNRE--------------------------DPLKL
GRMYDMPGD-INCPVT-----SLDVYL-SKLPPDP-----------------------------------------------------------------------------PAFYLHPLKLT-------
PEQIQEQSVWYKREP---------MGVNYL----GSMMPRIS-------I------AARL------------------SQRYTNHSLRTTTVRLLCD-AGLGAREIMA
LTGH-RSESSIRN----------------YWGAA
>CRE
FLGIAYNTLLRIAEIA----------RIRVKDISRTD--------------------GGRMLIHIG-----------RTKTLVSTA-------------------------------GVEKAL
SLGVTK-----LVERWI-SVSGVADDPNN-------Y---------------------------------------CRVRKNGVAAPS--------------------------------------------A
TSQ---------LSTRAL----EGIFEATHRLIYGAKD------DSGQR-----------------YLAWSGHSARVGAARDMAR-AGVSIPEIMQAGGW-TNV
NIVMN----------------YIRNL
>Lambda
AMELAVVTGQRVGDLC----------EMKWSDIV----------------------DGYLYVE-------------QSKTGVKIAIPTAL-------HID-------ALGI------
---------------SMKETL-DKCKEIL-----------------------------------------------------------------------GGETIIAS--------------------------TRREP------
```

---LSSGTV----SRYFMRAR-------K------ASGLSFE----------------GDPPTFHELRSLSARLYE--KQISDKFAQHLLGH-KSDTMASQ----------
------YRDDR
>FLP
LFLATFINCGRFSDIK----------NVDPKSFKLVQNKYL----------------GVIIQCLVT-----------ETKTSVSRHIYFF------------------------------S
ARGRIDPLV-----YLDEFL-RNSEPVL--KRVNR-------
---TGNSSS----NKQEYQLL-------------KDNLVRSYNKALKKNAPYSIFAIKNGPKSHIGRHLMTSFLSM-KGLT-ELTNVVGNW-SDKR
ASAV--ARTTYTHQITAIPDHYFALV
>FLP_Klac
LLLGAVGNCCRYSDLK----------NLDPRTFEIYNNSFL----------------GPIVRATVT-----------ETKSRTERYVNFY------------------------------
-PVNGDCDLLI-----SLYDYL-RVCSPIE---KT---------
-VSSNRP----TNQTHQFL-------------PESLARTFSRFLTQHVDEPVFKIWNGPKSHFGRHLMATFLS--RSEKGKYVSSLGNW-AGDRE
IQSAVARSHYSHGSVTVDDRVFAFI
>Tn916
EILILLKTGLRISEFG----------GLTLPDLDFE--------------------NRLVNIDHQLLRDTEIGYYIETPKTKSGE---------------------------------
-----RQVPMVEEAYQAFKRVL-ANRKNDK---RVEIDGYSDFLFLN----------------------
-----RKNYP---------KVASDY----NGMMKGLV-------------------------KKYNKYNEDKLPHITPHSLRHTFCTNYAN-AGMNPKALQYIMG
H-ANIAMTLN----------------YYAHA
>XerD
MLEVLYATGLRVSELV----------GLTMSDISLR--------------------QGVVRVIGK---GNKERLVPL
GEEAVYWLETYL-EHGRPWL---LNGVSIDVLFPS--------------------------QRAQQ---
-------MTRQTF----WHRIKHYA-------V------LAGID-----------------SEKLSPHVLRHAFATHLLN-HGADLRVVQMLLGH-SDLSTTQI-------
----------YTHVA
>At_Ti
MLLIGYAGGFRRSEIV----------GLDLK------------ADQTEDGRGWIEILDKGMLVTLR------------GKTGWREVEI----------------------------
--GRGSSDATCPVA-----NVETWI-KFAKLAH---GPLFRRVTGQGKS--------------------
-------VGSER---------LNDKEI----ARLVKRTA-------M------AAGIRGDLAEIER---------QFKFSGHSLRAGLASS----AEVDERYVQKHLG
H-ASAEMTRR----------------YQRRR
>Vlf1_AcNPV
VFCIMLGTGMRINEAR----------QLSVDDLNVLIKRGKLH--------------SDTINLKRK-----------RSRNNTLNNI-------------------------------
------KMKPLE-----LAREIY-SRNPTIL--Q---------I
SKNTSTP--FKDFRRLL-------E------ESGVE-----------------MERPRSNMIRHYLSSNLYN-SGVPLQKVAKLMNH-ESSASTKH------------
----YLNKY
>DIRS1
LVLCKMFGLARSSDLV----------KWSFKGLIIT--------------------PDSIKGPVI-----------NAKEQRSGVVSILE--------LTS-----------------LD
DTNSQVCPVR-----HLATYL-RASKGRR---KPHSGDSVFIK--------------------------N
EVNR---------SKLMIL----TQIVLSTL-------S------KSGID-----------------IVKFKSHSTRSAMASLLVS-NNVPFHVVKKMGRW-KSNDTV
DT----------------FYDKR
>Kangaroo
CCQFMWHTSYRGHDTG----------KLRLRDFRDPRG-GGPFRGFPLPLPDPFGAYPSLSLRIEQL-----------GTKTSKGRRAPPLE--------
L-------------------RPDPSPRHCFLR-----TLALYW-QLCHAPD--APPGSAISDYLFRPTDRG
HQR--------------------------FVERP---------FSSSAL----AMRVGKHL-------E------EAGV------------------YVGQTPHGFRRGTIQATQA-AGA
SRAELHAFSQI-RSAQVLER----------------YTDAS
>Pioneer1
RCRCRCRSTRRSCDTC---------------GARFASRPATPWPYWCTCATSCASCSCGRPLSAPTTLASWAWATSWTQCGRTSPTRAFRCL--
---------------------------RRGSTCAVCPAS-----ASRAGY-QDVPPGSCACHLAGASTCGAGLLHTARAGVVHVVVPPAGVPP-------------------
----------------TCCSGPSLRTSA--------------------------ASRTP---------LRTSTL----SARLKLHL-------Q------AAGL------------------YAGESCH
SFRRGALQAAAG-GGATSEELMGLSHI-RTPAVLGR----------------YLDEE
>DIRS-1_CR
CVLLLWQTAVRGHDLG----------KLGTGDFVNPDNTALPFQGFPLLPPWQWGSYLGPILCFCER-----------GTKTHKLARAPPIF------
--LM------------------PDVAEPRLSIPR-----VLALYM-ALCSAAD---APPGSAVADLLFRPLAP
DGKR--------------------------FKETP---------LSSSAM----GARLRMHL-------V------AAGL------------------YGGETVHSFRRGSLQNAQA-VG
LPPSSLLDLGQL-RTPAVLER----------------YLDSS
>SPPat1
LLILCFTGFLRYDEAR----------SLKCNDVSIY--------------------DDYISLNIA-----------KSKTDQYRQGNSVV----------------------------ISA
GVTEACPVK-----MVKKYI-NVARISL---DSNHFFFKPAYRSKGVSALI-------
----RKDKP---------LSYTTARERVISLLKEVC-------G------DANL---------------------GLHSLRAGGATMVAN-TSTKDRLWKIHGRW-KTD
KAKDG----------------YVVDT
>DIRS-1_SK
MILLCFSAFLRFDEVS----------SLRCCDVTVF--------------------DGHIAINIT-----------RSKTDQYREGNSVV---------------------------VAT
IDSVACPVS-----MLRRYV-KAASIDL---SNSLFLFRPMVRSGVKCAFV------
----KHNKK---------LSYTRARETIVCRLKEV--------G------GANL-------------------SFVLHSLRSGGATAAAN-AGVGDRCWKRLGRW-RS
ESALDG----------------YMMDS
>DIRS-1_SP
CFLAAFWGFLRIGEFT--ANSRSTSDVSLEGRDVIVDGSAP-----------------RRHVRLHIR-----------SSKTDQQGQGCYIL--------M---------
-----------PEADGCPLCPVQ-----AVLDYM-AIRPQSS---GAFFCR--------------------
-------FDGNH---------VTRREF----TAVLRKCL-------S------FLDLP-----------------VARFTSHSFRIGAATSAAM-AGFDSGEIQNMGRW-SS
DAHKR-----------------YVRIN
>DIRS-32_AC
AYTTAFFGALRLGEVV-ADSKSDGSGRALQLRDVSIG--------------------HNSLVIRIR-----------QSKTDQGGRGASLN--------I-------------
------QGLEPGRLCPVR-----ALSEYL-HMRRSSP---GTLFIH------------------------
--RNGTP---------LSRYQF----MAIFRSAL-------X------GLGLP-----------------AAEYGGHSFRIGAATTAAV-GGVPVDIIKTMGRW-KSAAY
AS-----------------YIRPG
>DIRS-53_XT
LFVIVYFAAMRISEAV--APNKLTPGGILRK-DIALM--------------------GDRLRINIR-----------VSKTDAVGKGCIIW--------L-----------------
--GHFVDSLLCPVR-----NFQTFC-DIRPESD---GPFFMH--------------------------
RNGEY---------LSRFQF----NKVFSMCL-------K------KLGIP-----------------DKNYRSHSFRIGAATQAAL-WGENERMIKKIGRW-SSNR
YKL-----------------YIRPS
>DIRS-25_NV
ACNLAYFGFLRSAEFTVPNAKSFSPALHLQLSDLAFDERSN-----------------PSCVRVWIK-----------ASKTDPFRKGCYIH----------------
-----------IGRGTYPLCAVD-----ALSTYI-IRSGDIQ---GPLFQF----------------------
-----QDGRP---------LTRAKL----TTWLRSIV-------A------AAGI------------------PXNFSSHSFRIGAASVAAQ-NGIPDHLIQTLGRW-RSGAY
QL-----------------YIRTP

>DIRS-9B_DR
MFTLAFFGFLRCSEIT--TTSKFNPSIHPTISDLTVLD-------------------KETISFFVK-----------QSKTDQIRKGHHIY------------------------
--IFDIPSPTHHFQ-----TLLAFL-QFRKLQD---PNPLSPLFTD------------------------
-DYNRP---------VTRFWF----QKHLKEIL-------R------LSGLS----------------PDSFSSHSFRIGAATTAAH-NGLSQSQIQALGRW-SSDAF
NS----------------YIRFS
>DIRS-30_NV
MCLTAFFAFLRVGEMT---VNQKEANLPLQLHQLSPILNSDNKVTGYKVSFQDFKHSYNQPTFSITIN-----------RQK--------------------
----------------------GICPVT-----ALTEYL-RLRGSRA---GALFLG--------------------
------IDGSP---------VSRSTF----SSLLCQAI-------K------YCGLD----------------SSKYKGHSFRIGAASHVTN-LGFSDAQIRTLSRW-KSN
AFLR----------------YIRIA
>DIRS-23_NV
M-------ARIANIVPLSIKAFNPTRDLTRGDVLCN--------------------EHGLIVTFK-----------HTKTIQFGQRRLHI-------PL-----------------
LRIPGSLLCPVA-----AYNNMI-RLVPASA---RKPLFLLLGH-------------------------S
GPFA---------LTKSRF----VTEFRLAL-------C------SVGVAH---------------ADSYRGHSFRRGCASWAFN-HGVPGELIQLYGDW-ASDSY
KL----------------YLEFS
>DIRS-1_NV
VMLMSLVSAQRGQTIH----------MLNLKDMINE--------------------ETGITFVMS----------VPMKQTKPGSKSVTV-------QFK--------------
---PYHVDPTLCVVT-----ALREYV-LRTETLR--GECKQLFISYL-----------------------
---KPFKP---------VSRSTI----SRWVKVVM-------K------TAGIN----------------TDKFKPHSTRAASTSNANI-SSVPVDQIMSVAGW-SSAS
TFAK---------------FYNKP
>DIRS-2_NV
TMLIALLSGQRCQTLH----------ALDISTMDLT--------------------DKHCIFYIQ----------ELLKSSRPSKHFGRL-------ELK----------------
AYENDKRLCVVT-----IIKEYV-ERTKLLR---GNDSRFLISFQ-------------------------
KPHKP---------ISTDTI----GRWLKKVL-------A------KSGIN----------------INKYSAHSTRSASMSAGKT-ANISIKTIMDAAGW-SNVGTF
RT---------------YYDKP
>DIRS-1_DPu
ATLFALATLLRVSELA----------SIPFSSIKFT--------------------ENSVQFALS----------KPRKAQRNGPLQSFT-------L------------------PAC
PDSDACPVA-----SLRSYV-ERTGTNR--PSKDEGMLFISTI-------------------------AP
FGP---------VTSNTV----GRWIKNFL-------K------TAGID----------------TSIFSAHSTRSAAASLAVA-RGLSIDAVLQAGHW-ASQTTFGR--
--------------FYNRG
>DIRS-10_DR
ALLVALASVKRVGDLE----------AFSVS------------------------DSCLEFXPAYSHVVLRPRPGYVPKVPTTPFKDQVV-------SLQ-------AL
PAEE----ADPALSLLCPVR-----ALRTYV-DRTQSFR--SSDQLFVCYGGR-------------
-------------QKGSA---------VSKRRL----AHWRVDAI-------S------LAYLSQGEPC------------PPGVRAHSTRSIASSWALA-RGASLTDICRA
AGW-ATPNTFAR----------------FYNLR
>DIRS-2_DR
AFLLAISSLKRVGDLQ----------ALSVA------------------------PSFLEFAPGMSKAFLYPRPGYVPKVPT--HVARPA-------VLQ-------AFH
PPPFQSSDQEKLNLLCPVR-----ALNTYV-NRVINWR--KSEQLLVCFGPS---------
----------------KRGSP---------ANKQTI----SNWIVETI-------S------FTYQAAGRPA------------PKFVKAHSTRAVGASKASI-SGSALSDICL
AAGW-STPHTFVR----------------HYQLD
>DIRS-23_XT
VFLVAVCSARRVGELQ----------ALSCK------------------------NSCLQVFPD--RIILKADPLFRPKVSSNFHRNFEV-------ILP-------AFFA
EPRNK-VEEKLHLLDAKR-----CVLFYL-NKVKPFR--ISHNLFVSFWGK-----------
---------------NKGKR---------ASKTSI----SRWIKQAI-------S------LAYSASGKSI------------PPNLKAHSTRAVSASQAEV-GGVSVDQICRT
ASW-ASFRTFAE----------------HYRLN
>DIRS-9_XT
LFLVAVASACRVGELQ----------ALSCS------------------------PGHISFLHD--RVILKPVKSFLPKVVSTFHLKREI-------SLP-------VFSA
DVQ---NLEELQKIDAVR-----CLKHYL-EVSNSFR--RSDKLFVIPAGC--------------
-----------RKGLG---------AATSTI----SRWITICI-------E------KAYQAQGKLA------------PEGLRAHSTRAVSTSWAAW-AEVPSAQICEVAS
W-SSARTFIR----------------HYQLD
>DIRS-1_XT
VFLVAISSIRRVSELS----------ALSCS------------------------PPFLIFQED--RAVLRTTPGFLPKVVSPFHINTEI-------SLP-------SFCSNP
SNE-KEAKLHRLDVVR-----ALRTYI-SRTKSLR--RSDALFVLPSGP-----------------
--------KKGLP---------ATKTTL----ARWIKEAI-------R------RAYLAKRRTP------------PLRLRAHSTRALGASWAHR-HMASADQVCKAA
TW-ASLHTFTK----------------FYQFN
>DIRS-8_XT
TFLIAITSAKRVSEMA----------ALSSK------------------------EPWLTLHHD--KAVLRTSPGFLPKVVTERHMNQDI-------ILP-------SFC
PKPSNE-KERLLHKLDVVR-----ALRIYL-KRSADYR--QSESLLISYSTT------------
-------------QKGKA---------VSKRTI----ARWLVETI-------H------TAYDRKNVPR------------PFAVKAHSTRAQSTSWALQ-NLATADQICRA
ATW-VSPNTFIK----------------FYKLN
>DIRS-1_ACa
AFLTAITTARRACELC----------ALRAD------------------------EPYLRFHKD--KAVLRTDISFLPKVVSKFHLSQDI-------ILP-------AFFQ
NPVSP-LEHALHALDVRR-----ALAYYV-DRTRAYR--KSPRLFVKYRQD------------
--------------AMGLP---------ITSQRL----SAWIVAAI-------R------LAYNLAGREP------------PTTLKAHSTRAVATSCAFM-KGVPLEEVCRA
ATW-ATPSTFVS----------------HYKVD
>Cry_Cn1
AQAVGIHGLLRADDQL----------RITLSSMSLRLF-------E-DEGPTP----CRGVVFAIR-----------EGKTTHDGQIQYST-------LLR-------N
KDV-----------TRCPVS-----FLVLYL-FARFHFSE-EPFINSDVSFPSLKNRQDWYHIPLFVSRQSNAV---
-------------------------------TR---------LKYDAL----NKSVRKAL-------Q------SCNI-----------------HCRASTHTSRKWGAQLAED-GGAPEE
DIMRQGRW-CTKVMETV---------------YLSKF
>CryF1_CGlo
DFLFGNHMLLRQSNRR----------PMELPDCFRLEL-------P-NEGQKSKEFPTYALVVVMN-----------QGKTNQHGRMEYGA-------AL
R-------HRDA-----------RCCLVS-----ALAFYL-FWRWQVEQVEP------FPTFQCSEDWYDIKVLRRSAKEAT---------------------------------
---------------------------------------KE---------LSAQTA----NSWTSRLY-------A------ACGI-----------------RTSKISHAPRVAAAQNADM-DGA
SEGQIRRAGRWNNGDQLTGC----------------YLTSL
>CryF1_TS
CFLFGNSMLLRLSNRL----------PMELPDLFSMPL-------P-NEGPKGK---GWCLVTVMD-----------QGKTNQHGRLEYGA-------ALR--
------HRDH-----------QSCLIG-----ALATYF-FWRWHCSG-ES------FPCFRTSQDWYNIKVLKRDNNHLT-------------------------------------
------------------------------------EP---------LSDSTA----ASWTRRLY-------S------EAGI-----------------KSSKVTHAGRVSGARLAEL-NGVSE
DQIRRGGRW-NADQMTGC----------------YLTTL
>MarCry1_FO
DLLFGHYYLLRGENRR----------KMELADLSLLDY-------PSSEGPTP----CGCLVTLLR-----------DGKLNKTAKKEFMG-------ALR-----

---HKDP-----------LFCTQG-----ALAQLF-FWRWHVAG-EP-------SPSFRRRQDWYRIKVLV--GRDRE--
--------------------------------QE---------LSYPTQ----LQETWRIF-------G------AAGL-----------------MASKKTHLPRRVGAQDAET-HGTSLA
QISQAGRW-NQSVLCQA----------------YLTRL
>CryF1_CI
DFLMGHSMLLRGESRR----------TAQLADLFTLEL-------T-NEGPTP----CFPMVLIMG-----------NGKTNQMGWIEYAT-------VMH----
----HRNL-----------LLCTMA-----QTAFYL-FYRWDIVR-EP-------PPQFHNHQDWYQLHLIK--RDDVR--
--------------------------------KP---------LSYKTQ----LDWIRCIY------S------GTGL-----------------SGLKKTHAGRAAGARHAEQ-VGVSEG
QICRAGRW-NSDALSQC----------------YLTNI
>CryF2_TS
DFLFGHNMLLRGEDRR----------HLELADLFTLRM---------DEGPTP----CWPMILMKL-----------NGKTNQFGRLEYMG-------VVR---
-----HKDP-----------LLCTIC-----HTAFYL-FHXWEIMH-EP-------VPQFYQRQQWYKXVLFK--GSDSE--
--------------------------------HS---------FSYETQ----LKWINQVF-------Q------SIGL-----------------NSKKKTHSGRSSGARHAEL-QGVDEN
SIRRAGHW-NQDSMSNC----------------YLSEL
>CryF1_RO
CFSMSHAMLMRSETAL----------GTQLPDLFIMEL-------K-NQGPST----CFAIVATIT-----------FGKTNKDGKIQYGS-------ALR-------
HRDV-----------EVCPHG-----AFAQYF-FSLFHHQN-LP-------FPNFSTRRDWY--
--CGV-----------------HSSKLTHINRKSAINMVAN-EGVSGDQQRQVGRW-GSDRMVGC--
---------------YLSGL
>CryF1_PI
SHFLCHACLLRGESAR----------NLDLPDLFSVIL-------E-HEGFTE----CRALVMIME-----------QGKTNQFGRREFGS-------CIR-------
HRNV-----------EVCPVG-----ALALYL-FWRWSVQK-EA-------VPDFLVPERWYDIKLLK-SNKDIT---
----------------------------TP---------MTYRAH----YDATVKAF-------S------ALGM-----------------RSKAKTHAARGSGARMAEL-AGATESQI
RRLGRW-NASAMEGC----------------YLSAL
>CryF2_PS
DFLVGHALLARGESRR----------FIQFPDMLSLEL-------T-DEGPQV----CSPLVVIMR-----------KGKSNQVGRVEYGA-------AMR-------
-CKDV-----------MLCPLN-----AVALYL-FWRWHVDL-EP-------FPSFTDRKLWYDIRLLK--GKCAT---
----------------------------RD---------IAYNTQ----LEHVKSAF-------T------ACGI-----------------DSSVWTHANRGSGAKLAEL-QGADENQIR
RAGRW-NGERMEAC----------------YLTTL
>CryF3_PS
AFLLSHYALMRGESAR----------LMELADLHSIVL-------E-NEGYSV----CRALVMVMT-----------QGKTNQVGRIEVGA-------CMR-----
---SKKV-----------EICGHG-----LLGFYL-FWRWHVDK-ES-------FPDFTKSEQWYPIKLLK-TGKDPT--
--------------------------------KP---------MSYKVH----RAAIVDAL-------D------HIGL-----------------RSRAKTHLGRGSGSRMADL-GGASEA
QIRRLGRW-NTQAMEKC----------------YLTSL
>CryI1_NVi
VLIFGILGALRRGELT----------NITTDDIEDD--------------------STRLLIKIP-----------VTKNNVPRSFAV-----------------------------------RG
EFYQ-----ICKNYM-NLRPTEI--DKHKRFFIHYIDGK--------------------------CTRQP--
--------IGLNTI----GQMPKQIA-------D------WLKLPN----------------PHLYTGHSFRRTSATLLVD-GGGNLTDLKRHGGW-KSSTVAEG-----
------------YINES
>CryI1_TC
VTIFGIFGACRCDELL----------SLTPNDVEDT--------------------GKYIIVTLR-----------NTKNFTTRRFTIT------------------------------DEE
CRFQPCV-----LYRKYA-SLRPTQA---ESLRLFLTYRGGK--------------------------CISL
N---------AGQHTI----GGIPKKIA-------S------YLKLSE----------------PELYTGHSFRRSAATMVVD-SGGDILALKRAGGW-KSSAVAEG---
--------------YVEDS
>CryI2_TCa
ALIFGIAGALRREELY----------KMKCEDVNDT--------------------GNVLIITVP-----------DTKTHIERRFTVI-----------------------------GETP
KHNLNLID-----IYKKYK-NQRPTNV--KTDHFFLQYRQGK--------------------------C
KTQV---------VGINTF----SKIPSQIA-------T------YLNLPD----------------PSSYTGHAFRRSSASLLVD-SGGDLMQLKKHGGW-KSSTVA
EG----------------YVDES
>CryI1_RPro
VFVIGISGACRSCELV----------QLELSQIKFL--------------------EDFITINIP-----------NTKNNVQRTFFIS------------------------------NEVG
LYNFKQ-----IFQQYI-NLRPKHT--KHPRLLVGYNHGK--------------------------CNIQ
P---------VGINTI----SKIPSRIA-------K------YLKKDN----------------FQRYTGHAFRRTSASLLAN-EGGDLIQVKKHGGW-KSSTVAEG---
--------------YIERS
>CryI1_AA
AMVVGVCGACRREEIT----------KLTLNDIEDM--------------------GDSVKITIP-----------NTKTKILRQFVITA-----------------------------G
NAPGVDMLK-----LFRSYR-QLRPADT---NHLRFFVCYRSGK--------------------------
CTKQP---------IGINTI----AKMPKIIA-------E------FLKLPS----------------PADYTGHCFRRSSASLLAD-SGVDISVLKRHGGW-KSSTVA
EG----------------YVENS
>CryI1_CQ
ALIIGVYGACRREEIM----------KLSLDDIEDL--------------------KDSIKITIP-----------NTKTKIMRQFIVTK------------------------------GEA
PGVDMLK-----LFRLYA-EKRPAGT--GHSRFFVSYRFGK--------------------------CTK
QA---------MGINTI----AKLPRIIA-------E------HLKLPS----------------PELYTGHCFRRSSASLLAD-SGVDISVLKRHGGW-KSSSVAEG--
---------------YVENS
>CryA1_NV
WFNLCYYLGRRGREGW---------RGLTKTSLEFKHDD------------------LEQKYVCIK---------HTEQSKNYQGGY------------KQK--------D
QDYSDLRMYGIPGS-PLDPIA-----ALEKML-EKLHPEC--DALFQTPLVN
F--------DKKGS---CWYKNEP----------LGKNSI----SKLMPKIS-------Q------KAGL-----------------SKVYTAHSVRALTITSLHQ-AGDDAK
QICAITKH-NNEQSLSS----------------YIKDS
>CryA1_SP
FMDVMLYFCNRGRENL---------REMTLDSFDICDE-------------------GGKCSITLK---------DTLTKNNR---------------ADK--------LEKSQ
GGVMIPTNG--PRCPVA-----SFLRYK-DKLNPQC---KSFWQRPANAQAK
RELKSNPSSSD---QWYCNAP----------LGKNSI----GDKMKTIS-------S------RAG-------------------TKAYTNHCLRATSISTLQN-AGFRDR
EIMSVSGH-KAETSLKH----------------YAMTS
>CryA2_SP
FVDFMTYFCNRGRENL---------RELKPDDFRLETDE------------------DGLRYITKR---------DQLTKNNR---------------EDD--------DEVS
NNGVMYEIPGS-SKCPVE-----SFMQFV-SKLNKDC--PFLWQKPKAKK--
------PEDGD---NWYCNAP---------VGKNTM----GNKMKQIS-------Q------KAGC-----------------SKLYTNHCLRATCITTLDR-AGFESRDI
QSVSGH-HSEQSLRN----------------YCNTS
>CryA1_SK
WFSIMLYFCRRGREGQ---------RDLRGSHFMVKSDD------------------NGSKYVIQV---------GSEVSKNHQ----------------CD--------DDGV
SGGIMYANNSNPRHCPVR-----AFELYL-SKANRRC--DALFQRPRDNY--
------SADDD---VWFENKP---------IGKNTL----SEMMALIS-------K------CASL-----------------SQRYTNHCIRATSITILSE-AGFNNRHTM

SVSRH-RXELRAGY------------------XN
>CryA2_SK
WFYNTVHFGLRGNTEH---------RNMCWGDVSLCTDS-----------------SGREYLEFS---------ERQTKTRTGE-------------NPR-------DT
RKVKPKMWDVPANPRRCPVA-----IFKKYL-SLRPAGY--TNSDDPYYIATH
SRGL-------PRPGE---QWFRHQP---------IGINKI----GSLMKNMA-------T------AANLPP---------------NKRLTNHSARKHLIQKLSD-QNI
PPTQIMQISGH-RNIQSVNA----------------YSTIS
>CryA3_SK
WYNNTKLFGLRGNNEH---------RQMEWGDVVLQSD------------------ENGEFLVYN---------ERLTKTRHGE--------------PG-------NTR
SFAPKAYATPKTPDICPVR-----AYKAYA-AHRPDRM--NCSSAPFYLGIEYV
PC-------TIAKG---IWFRNQP---------MGINKL----TTIMKSMS-------K------AADL-----------------TGKLSNHSARKTCVQRLLD-AGVPPN
TAAQLSGH-KNVSSLNR----------------YSVPN
>CryA1_OL
WFDIQLCLARRGREGC---------RELTMASFSIHRDE-----------------AGAEYLSLS--------HNPDTKNHKTP-------------NDP-------HKQ
NLRGFMFARPGD-PLCPIQ-----SFKKYI-SKCPPDA--KSFYLHPKRSV----
----TAAAE---VWYSREP---------MGVNYL----GAMLKKIS-------E------EVGL-----------------SQIYTNHSLRSTAVGRLSD-AGLESRQIMS
VTGH-RCESSLQA----------------YWAPS
>CryA1_LG
LFLFGLHFALRAAQEH---------RNLRFGQLSVKYDL-----------------EGKKYLQYI---------EDVSKTNQGG-------------LDH-------RKV
SPKMTRAYENSNPLRCPVR-----LYEKYI-ALRPKNG--TVDAFYLRPKKM
P------------CSDVWYCDSP---------VGIHTI----QSTVKRLC-------Q------SAGI-----------------IGNFSNHSLRATAATRLYQ-AGVDEQLIT
EKTGH-RSNAVRA-----------------YKRTS
>CryS1_ALai
LLSLLWYMFGRASDLS----------LVRKQNVSVDA-------------------GGVFFVRFI-----------RVKTCEEQGLSIF---------------------------PD
-TDFITCPLY-----AIALAL-ITQTAPHADLL----------------------------ENLPV---QLPSTSVVLSPETPLIDLL--------DHPAAVIGHQDT----
--------------------ITTGPAMSS------TLHIHV---NRLLDRVA-------K------RAGV-----------------EQQLTSHSFRRGGAQHANSSSQLTARW
IFDRGAW-NMSTTNKA--------------FNYVFNT
>CryS1_PU
LLALLWYLFGRASDLL----------LLQKQHVSVCS-------------------RNVFFLRFV-----------RVKTAEEQGLTLF---------------------------P
DR-NFVTCPLL-----AIALAL-ATQRRASPSLL----------------------------DHLSDV---QHEEGCDALPASISLMERL--------LGSAAAVDGTK
ER-------------------------AASAP---------GVHSYV---NRLLRRIA-------A------KAGV-----------------SKPLSLHSFRRGGAQHANGEPDVS
LQWILDRGAW-NLSTTNKA--------------FAYVFNT
>CryS22_PI
LLTMMWHTFGRAVDTC----------FARKQQLSISA-------------------SGELFLHIA-----------RIKTSMVQGVSIY---------------------------KS
PERWQQCMLH-----AFGLLF-ICYDEPSEYLF----------------------------PLAPNFAESDLPGNKPYSQEEAVL------YWGQLLEDDNSTS
EPPQKR-------------------------ERKRP---------NIASYI----TEVIRDSL-------KSMPPDLSQTV-----------------TPNMTSHSIRRGAAAYAN
ASPKLSIQWISTRGAW-LLESLTKA--------------FAYIGTT
>CryS1_PTri
YLLTLRYLAGRGQEVA----------MISRSRVSLGEPSEWADSG------------DKTFVVRLW-----------RSKVSHEQDLSIV-----------------------
-----PHQSEMLLDWVF-----AFAYSA-VMNTNPNDSLFPTFAEKVELRNLSAGNINDEEIGNETTQDST-------------------------------------
-------------------------------LKAKV---------DSSKKVTKYFQALLERLIKTLTGEEE------TVGLDPPANAAYYYSGLLHKHGISAGLSTHS
AKRSAVEMANESALLLTTWVCFRAGW-LMKAVHTI--------------FDYLSFN
>CryS2_PU
LLVTQWHCMGRAVETG-----------LRHSSMKFVPKP-----------------IGALQFIVG-----------RSKTNVHQHVHAF---------------------------
CDRESWEADIFH-----AKASHL-ATCANVD---DE-------------------------FQF
RN---------VTAKMI---NTILSNVC-------G------QAGAEE---------------LSKFTSHSGRRGCASEALSHNGVSVTEVIARGGW-AFDAVSR
V--------------FLYFCGR
>CryS12_PI
LVVMMWYMYGRSSDAE----------QLEKQQLSILP-------------------GGVLFLRFK-----------RVKTALLQGISLF---------------------------K
DPSNFLTCPLF-----TLASAL-AMQTAPSKRIF------------------------------PQFLSTRRSTTCAVDVEELSLVELL-----EADSIPALLEDSQ
SAP-------------------------KRTVP---------GAQAYV---NRLLVRVK------TLGDEK-QIRL-----------------TTGLTSHSFRRGAAMHAND-G
SVAENWIIERGGW-QLDRVNKA--------------FGYMLGT
>CryS9_PS
LLNILWYLLGRSSDTM----------CLVKNQVAVYP-------------------GGCLFITFK-----------RMKSASYQGASIF---------------------------HD
PNDFSSCPIH-----ALALAT-IMQSTPSEFLM----------------------------DQLPR---DTQELLPTEIGETPLRELL-----DARTRSPATSNSES
PT--------------------PKAPAKKATVP---------GIHAYV---NRVLQKWS------GVCQTE-GTNL-----------------TPGLSSHSFRRGAAQNAN
SDSKISTPWILDRGGW-SMSAVSKA--------------FNYIVGT
>CryS18_PI
LLALMWYAFGRASDLG----------FVAKSNLTVSA-------------------DGVVFVRLL-----------RVKTSEEQGISIF---------------------------PD
QVSFVTCPLH-----AIAMAL-VMQDTPSSRLL----------------------------DHPQLA---TGDEERIVTPTDISLAEAL-AAC-DGDDTETASET
SSKPR-------------------------TKKRKTNDE----SMKIHAYV---NRIVKSAS------EAQA---RARP-----------------TPNLTSHSFRRGGAQH
ANGDASLSAQWIFDRGSW-NVTSTSKA--------------FAYVFNT
>CryS4_PS
LLSIMWYALGRASDLA----------FIQKRNLSVGS-------------------GNVLFLRLI-----------RAKTSEEQGLSLF---------------------------PD
KSSFITCPLH-----AIAMAL-AMQTFPVSSVL----------------------------DLEHLSGLNVGDEAP--ATEATPLTDAL-VHCSDDDEPANATP
EATSPR-------------------------RKSTP--------LKMQGYV---NRVLKAAS------ARQA---DAGV-----------------SSGLSSHSFRRGGAQHAN
ADASLSPQWIFDRGSW-NMTATNKA--------------FAYVFNT
>CryS1_PS
LACLMWHCFGRSSDLG----------YISKQHITVSA-------------------DNVFYVRML-----------RVKTAEEQGLTLV---------------------------P
DRDDFLTCPLH-----TLAVAL-VMQEAPCVSLL----------------------------SQLPA----LATTAGTSLDAGAPLLEVLRVSVADSSDTAAALE
ASLSPPSTASLLPTPSPPSSNPSQATTASLPAQLTASSPARSSLGRGEDGVQAYV----NRFLKRVA-------E------PAGA-----------------TSN
LTSHSFRRGGAQHANGDERLAAQWIFDRGSW-DMTRTNKG--------------FAYVFNT
>CryS1_HAra
LVCLMWHVFGRASDLS----------LVLKQGLSISA-------------------NNVLFVRLI-----------RVKTSEEQGLSLF---------------------------PD
KETFSTCPVH-----AIAVAL-LMQSAPCISLL----------------------------PQLTT---KIDVSDPASTVSIPLVELL--------EGGCHVEAGIIKE---
----------------------TTSGPANTR----APGVHSYV---NRILKNSA-------K------RAGV-----------------ESELTSHSFRRGGAQHANGNPQLS
AQWIFDRGAW-NLTATNKA--------------FSYVFNT
