# Supplementary material for: Crypton transposons: identification of new diverse families and ancient domestication events
Source: Mob DNA. 2011 Oct 19;2:12. doi: 10.1186/1759-8753-2-12 (PMC3212892; doi:10.1186/1759-8753-2-12)
Supplement: Additional file 5 — PDF file listing the accession numbers for DUF3504 genes. [file 1759-8753-2-12-S5.PDF]

Additional file 5. Accession numbers for DUF3504 genes.

Gene	Species	Classification	Accession number
WOC	*Drosophila melanogaster*	Arthropoda/Diptera	CAC01697
	Anopheles gambiae	Arthropoda/Diptera	XP_322022
	Apis mellifera	Arthropoda/Hymenoptera	XP_624959
	Nasonia vitripennis	Arthropoda/Hymenoptera	XP_00165299
	Tribolium castaneum	Arthropoda/Coleoptera	XP_972303
	Acyrthosiphon pisum	Arthropoda/Hemiptera	XP_001943720
	Pediculus humanus	Arthropoda/Phthiraptera	XP_002430505
	Lepeophtheirus salmonis	Arthropoda/Siphonostomatoida	ADND01132817*
	Ixodes scapularis	Arthropoda/Ixodida	XP_002403753
	Varroa destructor	Arthropoda/Acari	ADDG01000056*
	Schistosoma mansoni	Platyhelminthes/Trematoda	XP_002577579
	Schistosoma japonica	Platyhelminthes/Trematoda	CAX69524
	Aplysia californica	Mollusca/Gastropoda	AASC02011980*
	Pinctada maxima	Mollusca/Bivalvia	EZ420514*
	Saccoglossus kowalevskii	Hemichordata	ACQM01067576*
	Strongylocentrotus purpuratus	Echinodermata	XP_793574
	Ciona intestinalis	Chordata/Urochordata	XP_002127329
	Branchiostoma floridae	Chordata/Cephalochordata	XP_002590281
ZMYM2	*Homo sapiens*	Chordata/Mammalia/Primates	NP_003444
	Mus musculus	Chordata/Mammalia/Rodentia	NP_083774
	Canis familialis	Chordata/Mammalia/	XP_850020
	Monodelphis domestica	Chordata/Mammalia/Didelphimorphia	XP_001366267
	Ornithorhynchus anatinus	Chordata/Mammalia/Monotremata	XP_001516313
	Gallus gallus	Chordata/Aves/Galliformes	XP_001233738
	Taeniopygia guttata	Chordata/Aves/Passeriformes	ABQF01007661*
	Anolis carolinensis	Chordata/Reptilia/Squamata	AAWZ02018035*
	Xenopus laevis	Chordata/Amphibia/Anura	NP_001086553
	Danio rerio	Chordata/Actinoprerygii/Cypriniformes	XP_001923572
	Tetraodon nigroviridis	Chordata/Actinoprerygii/Tetraodontiformes	CAG08242
	Gasterosteus aculeatus	Chordata/Actinoprerygii/Gasterosteiformes	AANH01012347*
	Oryzias latipes Hd-rR	Chordata/Actinoprerygii/Beloniformes	BAAF04078680*,
	Oryzias latipes HNI	Chordata/Actinoprerygii/Beloniformes	BAAE01132309*, BAAE01132311*, BAAE01132314
	Callorhinchus milii	Chordata/Chondrichthyes	AAVX01071030*, AAVX01003033*, AAVX01219755*, AAVX01021011*
ZMYM3	*Homo sapiens*	Chordata/Mammalia/Primates	NP_005087
	Mus musculus	Chordata/Mammalia/Rodentia	NP_062805
	Canis familialis	Chordata/Mammalia/	XP_857608
	Monodelphis domestica	Chordata/Mammalia/Didelphimorphia	AAFR03060006*
	Gallus gallus	Chordata/Aves/Galliformes	XP_420201
	Taeniopygia guttata	Chordata/Aves/Passeriformes	XP_002187810

	Anolis carolinensis	Chordata/Reptilia/Squamata	AAWZ02030824*
	Xenopus tropicalis	Chordata/Amphibia/Anura	AAMC01038517*, AAMC01038515*
	Callorhinchus milii	Chordata/Chondrichthyes	AAVX01074815*, AAVX01617007*, AAVX01227604*, AAVX01012004*
ZMYM4	*Homo sapiens*	Chordata/Mammalia/Primates	NP_005086
	Mus musculus	Chordata/Mammalia/Rodentia	NP_001107871
	Canis familialis	Chordata/Mammalia/	XP_849462
	Monodelphis domestica	Chordata/Mammalia/Didelphimorphia	XP_001380579
	Ornithorhynchus anatinus	Chordata/Mammalia/Monotremata	AAPN01136168*
	Gallus gallus	Chordata/Aves/Galliformes	XP_417783
	Taeniopygia guttata	Chordata/Aves/Passeriformes	XP_002195746, ABQF01050374*
	Anolis carolinensis	Chordata/Reptilia/Squamata	AAWZ02030231*, AAWZ02030230*
	Xenopus laevis	Chordata/Amphibia/Anura	NP_001129637
	Xenopus tropicalis	Chordata/Amphibia/Anura	NP_001123397
	Danio rerio	Chordata/Actinoprerygii/Cypriniformes	AAI71718, XP_689392
	Tetraodon nigroviridis	Chordata/Actinoprerygii/Tetraodontiformes	CAG02312
	Gasterosteus aculeatus	Chordata/Actinoprerygii/Gasterosteiformes	AANH01003783*, AANH01002133*
	Oryzias latipes Hd-rR	Chordata/Actinoprerygii/Beloniformes	BAAF04001537*, BAAF04025036*, BAAF04001532*
	Oryzias latipes HNI	Chordata/Actinoprerygii/Beloniformes	BAAE01076510*, BAAE01076509*, BAAE01045156*
	Callorhinchus milii	Chordata/Chondrichthyes	AAVX01060100
QRICH1	*Homo sapiens*	Chordata/Mammalia/Primates	NP_060200
	Mus musculus	Chordata/Mammalia/Rodentia	NP_780352
	Canis familialis	Chordata/Mammalia/	XP_850904
	Monodelphis domestica	Chordata/Mammalia/Didelphimorphia	XP_001367745
	Ornithorhynchus anatinus	Chordata/Mammalia/Monotremata	XP_001505372
	Gallus gallus	Chordata/Aves/Galliformes	XP_001233527
	Taeniopygia guttata	Chordata/Aves/Passeriformes	XP_002187824
	Anolis carolinensis	Chordata/Reptilia/Squamata	AAWZ02016385*
	Xenopus laevis	Chordata/Amphibia/Anura	NP_001083416
	Danio rerio	Chordata/Actinoprerygii/Cypriniformes	NP_001020633
	Tetraodon nigroviridis	Chordata/Actinoprerygii/Tetraodontiformes	CAG06870
	Gasterosteus aculeatus	Chordata/Actinoprerygii/Gasterosteiformes	AANH01007721*
	Oryzias latipes Hd-rR	Chordata/Actinoprerygii/Beloniformes	BAAF04090792*, BAAF04002983*
	Callorhinchus milii	Chordata/Chondrichthyes	(AAVX01090275*, AAVX01277274*, AAVX01625381*),

			AAVX01006759*
	Petromyzon marinus	Chordata/Petromyzontiformes	Contig26917
KCTD1	*Homo sapiens*	Chordata/Mammalia/Primates	NP_001136202
	Mus musculus	Chordata/Mammalia/Rodentia	NP_001136203
	Canis familialis	Chordata/Mammalia/	XP_537294
	Monodelphis domestica	Chordata/Mammalia/Didelphimorphia	XP_001364001
	Ornithorhynchus anatinus	Chordata/Mammalia/Monotremata	AAPN01226528*, AAPN01261857*
	Gallus gallus	Chordata/Aves/Galliformes	AADN02021374*
	Taeniopygia guttata	Chordata/Aves/Passeriformes	XP_002195485
	Anolis carolinensis	Chordata/Reptilia/Squamata	AAWZ02009575*
	Xenopus tropicalis	Chordata/Amphibia/Anura	AAMC01108847*
	Danio rerio	Chordata/Actinoprerygii/Cypriniformes	XR_044928*, NC_007131*
	Tetraodon nigroviridis	Chordata/Actinoprerygii/Tetraodontiformes	CAAE01010805*
	Gasterosteus aculeatus	Chordata/Actinoprerygii/Gasterosteiformes	AANH01010078*
	Oryzias latipes Hd-rR	Chordata/Actinoprerygii/Beloniformes	BAAF04008989*, BAAF04028584*
	Oryzias latipes HNI	Chordata/Actinoprerygii/Beloniformes	BAAE01047086*, BAAE01024970*
	Callorhinchus milii	Chordata/Chondrichthyes	AAVX01135059*, AAVX01274183*, AAVX01152351*
	Petromyzon marinus	Chordata/Petromyzontiformes	Contig72738
KIAA1958a	*Homo sapiens*	Chordata/Mammalia/Primates	BAB85544
	Mus musculus	Chordata/Mammalia/Rodentia	NP_001015681
	Canis familialis	Chordata/Mammalia/	XM_862606
	Monodelphis domestica	Chordata/Mammalia/Didelphimorphia	XP_001375998
	Ornithorhynchus anatinus	Chordata/Mammalia/Monotremata	AAPN01441730*
	Gallus gallus	Chordata/Aves/Galliformes	AADN02057701*
	Taeniopygia guttata	Chordata/Aves/Passeriformes	ABQF01045188*
	Anolis carolinensis	Chordata/Reptilia/Squamata	AAWZ01002749*
	Xenopus tropicalis	Chordata/Amphibia/Anura	AAMC01060931*
	Callorhinchus milii	Chordata/Chondrichthyes	AAVX01026201*
KIAA1958b	*Homo sapiens*	Chordata/Mammalia/Primates	EAW59110
	Mus musculus	Chordata/Mammalia/Rodentia	NP_694798
	Canis familialis	Chordata/Mammalia/	XP_538791
	Monodelphis domestica	Chordata/Mammalia/Didelphimorphia	XP_001376013
	Ornithorhynchus anatinus	Chordata/Mammalia/Monotremata	AAPN01412958*
	Gallus gallus	Chordata/Aves/Galliformes	XP_429189
	Taeniopygia guttata	Chordata/Aves/Passeriformes	XP_002188379
	Anolis carolinensis	Chordata/Reptilia/Squamata	AAWZ01002749*
	Xenopus tropicalis	Chordata/Amphibia/Anura	NP_001123722
	Danio rerio	Chordata/Actinoprerygii/Cypriniformes	NW_001877113*(BX640460*)
KIAA1958L	*Ornithorhynchus anatinus*	Chordata/Mammalia/Monotremata	XP_001506858

	Gallus gallus	Chordata/Aves/Galliformes	XP_425905
	Taeniopygia guttata	Chordata/Aves/Passeriformes	XP_002193638
ATF7IP	*Gallus gallus*	Chordata/Aves/Galliformes	AADN02006037*
	Meleagris gallopavo	Chordata/Aves/Galliformes	NC_015011*
	Taeniopygia guttata	Chordata/Aves/Passeriformes	ABQF01015803*

* Nucleotide sequences including DUF3504 genes.
