# Supplementary material for: Crypton transposons: identification of new diverse families and ancient domestication events
Source: Mob DNA. 2011 Oct 19;2:12. doi: 10.1186/1759-8753-2-12 (PMC3212892; doi:10.1186/1759-8753-2-12)
Supplement: Additional file 6 — PDF file showing alignment of KCTD1, KCTD15 and related protein sequences in fasta format. [file 1759-8753-2-12-S6.PDF]

Additional file 6. Alignment of KCTD1, KCTD15 and related protein sequences in fasta format.

>KCTD1a_Human

---MSRP----LITRS---PASPLNNQGIPTPAQLTKSNAPVHIDVGGHMYTSSLATLTKY
PESRIGRLFDGTEPIVLDSLKQHYFIDRDGQMFRYILNFLRTSKLLIPDDFKDYTLLYEEAKYFQLPMLLEMERWKQDRETGRF
SR-------P---------------CECLVVRVAPDLGERITLSGDKSLIEEVF-PEIGDVMCNS--VNAGWNH-DSTHVIRFPLNGYCHLNSVQVLER
LQQRGFEIVGSCGGGVD-SSQFSEYVLRRELRR----TPRV--PSVIRIKQEPL-D
>KCTD1b_Human
MARMPGSGDCNT--SAGGSASAAAAAAENNGERGEGER-GAGGRGRRHSRPHYCSAGEEEEEEEEEEEDEIQEVQITGDEE----EEED
GGGGL--EEDEEEEEEEEMGLDWDEPLEPEDSAGEELEPEPVHMINMDQSAALEPEAPPRLLAPRARGGPPGDGSELDPDVL-----
---------------QRPERARLSENTRLATRYAVRIFREYLSEKAQSPDFETMDKGALCRVLRSFYAEARSKSGQLYSKSSLISIRSSLNRYLN
EPPYCRTLDLTKDPELRSANLTLAAVIRKLEEQGAGPVVQKQAITRADLRKLYTSSVFSTNTPFGLLNKVWFETCMYFCTRGREN
QRELEEDSFGLAMDEDGRKFVYFKSLG-----PYHKSRSSSWSKKRA------ESS---DEENLPRMYETGTEFCPYASFVKYLSKRNPLC
KAFFQRPRDHCSEGDVTWYENKAIGKN--LLGTRMQMLSKAAKLSKTYTNHCIGAVSIATLNSIAG-IGTK------LGS--PAPQGCYA-
EALNGA-----ARHHS-HHPPTHPSHHH--------RPQPP-------------SLGNTYILPKDSQVGPDVKSEAA----PKRALYESVF-----GSGEICG
PT-SPKRLCIRP-SEPVD----AVVVVSVKHDPLP-LLPEANGHRS-TNSPTIVSPAIVSPT----QD--
----------------------SRPN----MSRP----LITRS---PASPLNNQGIPTPAQLTKSNAPVHIDVGGHMYTSSLATLTKYPESRIGRLFDGTEPIV
LDSLKQHYFIDRDGQMFRYILNFLRTSKLLIPDDFKDYTLLYEEAKYFQLPMLLEMERWKQDRETGRFSR-------P---------------CE
CLVVRVAPDLGERITLSGDKSLIEEVF-PEIGDVMCNS--VNAGWNH-DSTHVIRFPLNGYCHLNSVQVLERLQQRGFEIVGSCGGG
VD-SSQFSEYVLRRELRR----TPRV--PSVIRIKQEPL-D
>KCTD1b_Mouse
MARMPGSGDCNT--SAGGS---AAAAAENNGDRGEGER-GAGGRGRRYGRPHYCSAGEEEEEEEEEEEDEIQEVQITGD------EEEDGG
GGL--EEDEEEEEEEEMGMDWEEPLEPEDSAGEELEPEPVHMIHMDQSAALEPEAPPRLLAPRARAGPPGDSAELDPDVL--------
-----------QRPERARLSENTRLATRYAVRIFREYLSEKAQSPDFETMDKGALCRVLRSFYAEARSKSGQLYSKSSLISIRSSLNRYLNEP
PYCRTLDLTKDPELRSANLTLAAVIRKLEEQGAGPVVQKQAITRADLRKLYTSSVFSTNTPFGLLNKVWFETCMYFCTRGRENQR
ELEEDSFGLAMDEDGRKFVYFKSLG-----PYHKSRSSSWSKKRA------ESS---DEENLPRMYETGTEFCPYASFVKYLSKRNPLCKAF
FQRPRDHCSEGDVTWYENKAIGKN--LLGTRMQMLSKAAKLSKTYTNHCIGAVSIATLNSIAG-IGTK------LGS--PAPQGCYT-DSLN
GS-----ARHHS-HHAPTHPSHHH--------RPQPP-------------SLGNTYILPKDSQVGPDVKSEAA----PKRTLYESVF-----GSGEICGPS-SP
KRLCIRPSSEPVD----AVVVVSVKHDPLP-LLPEVNGHRS-TNSPTIVSPAIVSPA----QD--
----------------SRPN----MSRP----LITRS---PASPLNNQGIPTPAQLTKSNAPVHIDVGGHMYTSSLATLTKYPESRIGRLFDGTEPIVLDS
LKQHYFIDRDGQMFRYILNFLRTSKLLIPDDFKDYTLLYEEAKYFQLPMLLEMERWKQDRETGRFSR-------P---------------CECLV
VRVAPDLGERITLSGDKSLIEEVF-PEIGDVMCNS--VNAGWNH-DSTHVIRFPLNGYCHLNSVQVLERLQQRGFEIVGSCGGGVD-
SSQFSEYVLRRELRR----TPRV--PSVIRIKQEPL-D
>KCTD1b_Dog
MARMPGSGDCNT--SAGGSAGAAAAAAENNGERGEGD---
--------------------AELDPDVL--------------------QRPERARLSENTRLATRYAVRIFREYLSEKAQSPDFETMDKGALCRVLRSFYAEARS
KSGQLYSKSSLISIRSSLNRYLNEPPYCRTLDLTKDPELRSANLTLAAVIRKLEEQGAGPVVQKQAITRADLRKLYTSSVFSTNTPFG
LLNKVWFETCMYFCTRGRENQRELEEDSFGLAMDEDGRKFVYFKSLG-----PYHKSRSSSWSKKRA------ESS---DEENLPRMYET
GTEFCPYASFVKYLSKRNPLCKAFFQRPRDHCSEGDVTWYENKAIGKN--LLGTRMQMLSKAAKLSKTYTNHCIGAVSIATLNSIA
G-IGTK------LGS--PAPQGCYA-EALNGA-----ARHHSHHHPPTHPSHHH--------RPQPP-------------SLGNTYILPKDSQVGPDVKSEAA
----PKRALYESVF-----GSGDICGPS-SPKRLCIRP-SEPVD----AVVVVSVKHDPLP-LLPEANGHRS-TNSPTVVSPAIVSPT----QD--------
---SRPN----MSRP----LITRS---PASPLNNQGIPTPAQLTKSNAPVHIDVGGHMYTSS
LATLTKYPESRIGRLFDGTEPIVLDSLKQHYFIDRDGQMFRYILNFLRTSKLLIPDDFKDYTLLYEEAKYFQLPMLLEMERWKQD
RETGRFSR-------P---------------CECLVVRVAPDLGERITLSGDKSLIEEVF-PEIGDVMCNS--VNAGWNH-DSTHVIRFPLNGYCHLNS
VQVLERLQQRGFEIVGSCGGGVD-SSQFSEYVLRRELRR----TPRV--PSVIRIKQEPL-D
>KCTD1b_Opossum
MARMPGSGDCNTSPSSSASASAAAAAAENNGERGEGERGGSGGGSSRPSHPHYCSAG-EEEEDDDDDEIQEVQITGDEEEEEEE
EEDGGGGLEEEEEEEEEEEEEEMGLDWDEPLEPEYSAGEELEPEPVHMIHMDQSTELDPESEPLLLAPRS---GPAEDPELDPDLL-
------------------QRPERARLSENTRLATRYAVRIFREYLSEKSQSPDFETMDKGALCRVLRSFYAEARSKSGQLYSKSSLISIRSSLNRY
LNEPPYCRTLDLTKDPELRSANLTLAAVIRKLEEQGAGPVVQKQAITRADLRKLYTSSVFSTNTPFGLLNKVWFETCMYFCTRGR
ENQRELEEDSFGLAMDEDGRKFVYFKSLG-----PYHKSRSSSWSKKRA------ESS---DEENLPRMYETGTEFCPYASFVKYLSKRNP
LCKAFFQRPRDHCNEGDVTWYENKAIGKN--LLGTRMQMLSKAAKLSKTYTNHCIGAVSIATLNSIAG-IGTK------LGP--PAPQGCY
A-EALNGG-----ARHHHHHHHPSTPHHHH--------RPQPP-------------SLGNTYILPKESQVVADVKAEAA----PKRALYEAVF-----GPGE
VCGPS-SPKRLCIRP-SEPVDAA--AVVVVSVKHDPLP-LLPEVNGHRS-TNSPTVVSPAIVSPT----QD--
------------------------------SRPN----MSRP----LITRS---PASPLNNQGIPTPAQLTKSNAPVHIDVGGHMYTSSLATLTKYPESRIGRLFD
GTEPIVLDSLKQHYFIDRDGQMFRYILNFLRTSKLLIPDDFKDYTLLYEEAKYFQLPMLLEMERWKQDRENGRFSR-------P--------
--------CECLVVRVAPDLGERITLSGDKSLIEEVF-PEIGDVMCNS--VNAGWNH-DSTHVIRFPLNGYCHLNSVQVLERLQQRGFEIV
GSCGGGVD-SSQFSEYVLRRELRR----TSRA--PSVIRIKQEPL-D
>KCTD1b_Platypus partial

---------------ENTRLATRYAVRIFREYLSEKAHSPDFEAMDKGALCRVLRSFYAEARSKSGQLYSKSSLISIRSSLNRYLNEPPYCRTL
DLTKDPELRSANLALAAVLRRLEEQGAGPVVQKQAITRADLRRLYTSAVLGAATPFGLLNKVWFETCMYFCTRGRENQRELEEDS
FGLAVDEDGRRFVYFKALG-----PYHKSRSPGWARKRA------EAAGGGDDDSLPRMYETGTELCPYASFVKYLAKRNPLCRAFFQRP
RDHCAHGDGTWYENKAIGKN--LLGTRMQMLSKAAKLSKTYTNHCIGAVSVATLNSIAG-IGAR------LGPQAP-------------------------
--APQ---------------------------------APAPQAPAPQAPAPL---PHD-----
---SRPN----MSRP----LITRS---PASPLNNQGIPTPAQLTKSNAPVHIDVGGHMYT
SSLATLTKYPESRIGRLFDGTEPIVLDSLKQHYFIDRDGQMFRYILNFLRTSKLLIPDDFKDYTLLYEEAKYFQLPMLMEMERWL
QERESGRFSR-------P---------------CECLVVRVAPDLGERITLSGDKSL-EEVF-PEIGDVMCNS--VNAGWNH-DSTHVIRFPLNGYCHL
NSVQVLERLQQRGFAIVGSCGGGVD-SSQFSEYVLRRELRR----TSRG--PSVIRIKQEPL-D
>KCTD1b_Chicken partial

---MDKGALCRVLRSFYAEARSKSGQLYSKSSLISIRSSLNRYLNEPPYCRTLDLTKDPELRAANLTL

AAVIRKLEEQGAGPVVQKQAITRADLRKLYTCSVFSTQSPFGLLNKVWFETCMYFCTRGRENQRELEEDSFGLAVDEDGRKFVYF
KALG-----PYHKSRSSSWSKKRA------ESS---DEENLPRMYETGTEFCPYASFVKYLSKRNPLCKAFFQRPRDHCSEGDVTWYENKA
IGKN--LLGTRMQMLSKAAKLSKTYTNHCIGAVSIATLNSIAG-IGTK------LGG--PPPPPHHH-PAPHHH-----HHHHHLLHHPAGGA
CYASAALNGGPRPRPP-------------A-ANPYVLPKDGDAAP-VKAEAAAVAPAKRALYEAVFPAGAAAGGEACGPSASPKRLCRRP-AE
PPNAA--AV-------------------------------PAVVV--V---MSRP----LITRS---P
ASPLNNQGIPTPAQLTKSNAPVHIDVGGHMYTSSLATLTKYPDSRIGRLFDGTEPIVLDSLKQHYFIDRDGQMFRYILNFLRTSKLL
IPDDFKDYSLLYEEAKYFQLQPMLGEMERWKQDRESGRFSK-------S---------------CECLVVRVAPDLGERITLSGDKSLIEEVF-PEI
GDVMCNS--VNAGWNH-DSTHVIRFPLNGYCHLNSVQVLERLQQRGFEIVGSCGGGVD-SSQFSEYVLRRELRR---TSRA--PSVIRI
KQEPL-D
>KCTD1b_Zebrafinch
MAAV--LI------
--------------PHTPLARGTEETYV--PSWPQTKPRTPYCRTLDFTKDPELRAANLTLAAVIRKL
EEQGAGPVVQKQAITRADLRKLYTCSVFSTQSPFGLLNKVWFETCMYFCTRGRENQRELEEDSFGLAVDEDGRKFVYFKALG-----
PYHKSRSSSWSKKRA------ESS---DEENLPRMYETGTEFCPYASFVKYLSKRNPLCKAFFQRPRDHCSEGDITWYENKAIGKN--LL
GTRMQMLSKAAKLSKTYTNHCIGAVSIATLNSIAG-IGTK------LGGLHPAGGGCYTPAALNGG-----P------------------------RPWPP-------
-------A-ANPYMLPKDGDAAP-VKAEAAAVAPAKRALYEAVFPAGAAAGGDACGPSASPKRLCRRP-AEPLDAAAPAVVVVSVKHDPL
PHLLPEANGHRS-TTSPTVVSPAIVSPT----QD---SRPN----MSRP----LITRS---
PASPLNNQGIPTPAQLTKSNAPVHIDVGGHMYTSSLATLTKYPDSRIGRLFDGTEPIVLDSLKQHYFIDRDGQMFRYILNFLRTSKL
LIPDDFKDYSLLYEEAKYFQLQPMLGEMERWKQDRESGRFSK-------S---------------CECLVVRVAPDLGERITLSGDKSLIEEVF-PEI
GDVMCNS--VNAGWNH-DSTHVIRFPLNGYCHLNSVQVLERLQQRGFEIVGSCGGGVD-SSQFSEYVLRRELRR---TSRA--PSVIRI
KQEPL-D
>KCTD1b_Lizard partial

--QD----------------
---SRPN----MSRP----LITRS---PASPLNNQGIPTPAQLTKSNAPVHIDVGGHMYTSSLAT
LTKYPDSRIGRLFDGTEPIVLDSLKQHYFIDRDGQMFRYILNFLRTSKLLIPDDFKDYSLLYEEAKYFQLQPMLGEMERWKQDRE
TGRFSK-------S---------------CECLVVRVAPDLGERITLSGDKSLIEEVF-PEIGDVMCNS--VNAGWNH-DSTHVIRFPLNGYCHLNSV
QVLERLQQRGFEIVGSCGGGVD-SSQFSEYVLRRELRR----TSRS--SSVIRIKQEPL-D
>KCTD1b_Frog
MARMPVTGDCTPGTADTDHLHAAPRAAQHRLQPGSAGE-------------------EEEEEEDDDDEIQEVQITGEEEEDDDDDE------LLLLE
DEDDLEDDMQLEWDP------EEGALLEPPYPH-------TPCHPAQRPALPA------GPRSAMVLGEDVLLQLGSAGGGGDGGGGCVGSPS
ERPERARLSENTRLATRYAVRIFREYLSEKSQSPDFESMDKEALCRVLRSFYTEARSKSGQLYSKSSLISIRSSLNRYLNEPPYCRTL
DLTKDPELRSANLTLAAVIRKLEEQGAGPVVQKQAITRADLRKLYTSCVFSSSTPFGLLNKVWFETCMYFCTRGRENQRELEEDS
FGLAMDEDGRRFVYFKALG-----PYHKSRSSSWGKKRSCPGGSTMES---DEENLPRMYETGTEFCPYASFVKYLSKRNPLCKAFFQ
RPRDHGSESDVTWYENKAIGKN--LLGTRMQMLSKAAKLSKTYTNHCIGAVSVATLNSIAG-IGTK------LGP--P-----YLTDSLNGS----
-RH-------------------------RPAMP----------------GNTFILPKPTGGLSEAKTET-----TKRSIYNSAHSIY--ASGDMGSPP-SPKRLCIRP-ADPSD--
--CVHVVSVKSDPQP-SCPETNGHMVLAASPAVTSPSVMSPVQVTKQD--T
RPN----MSRP----LITRS---PASPLSNQGIPTPAQLTKSNAPVHIDVGGHMYTSSLATLTKYPDSRIGRLFDGTEPIVLDSLKQHYFIDR
DGQMFRYILNFLRTSKLLISDDFKDYSLLYEEAKYFQLQPMLLELERWKQDKEAGRFSR-------P---------------CECLVVRVAPDLGE
RITLSGDKSLIEEVF-PEIGDVMCNS--VNAGWNH-DSTHVIRFPLNGYCHLNSVQVLERLQHRGFEIVGSCGGGVD-SSQFSEYVLR
RELKR----TSRA--SSVIRIKQEPL-D
>KCTD1b_Zebrafish
MARMPGSGDHCCRDVDEPDPMAGDR------------------------------------ERDGEEEIEEIQITGEEGEVS--------------------EEEAELEWEEC
GEPDSCAAAVETGEAVLMRNTDQLESV--AEGDLLFGPEGDMHRSRLSENTRLATRYAVRIFREYLTE
KAQSTDFESMDKHALCKVLRSFYSEARSKSGQLYSKSSLISIRSSLNRYLNEPPFCRTLDLTKDPELRSANLALAAVIRKLEEQGA
GPVVQKQAITRSDLRKLYTSAVFSASSPFGLLNKVWFETCMYFCTRGRENQRELEEDSFGLAVDEDGRKFVYFKALG-----PYHKS
RSASWTKKRS------DT----DDDNLPRMYETATEFCPYASFVKYLSKRNPLCKAFFQRPRDHCSDTDTTWYENKAIGKN--LLGTRMQ
MLSRAAKLSKTYTNHCIGAVSIATLNGIAG-IGSK------LAPL--RASPE--------------TVNGAHVLHCE
DELELKPQKQVTKR--PRTLVYPV----------SVAGPGASPKRQCARA-DTPT-----VTARVSSRCEP--------------GESPH------MLPS----QE----
---NRSNSVGMSTRP----ILAHS---PVSPLGTAGIPTPAQLTKANAPVHIDVGGH
MYTSSLATLTKYPESRIGRLFDGTEPIVLDSLKQHYFIDRDGHMFRYILNFLRTSKLLIPDDFKDYSLLYEEARYFQLQPLQVELER
WRSEQDSRFTSR-------M---------------CECVVVRVAPELGERITLSGDKALIEDIF-PEIGDVMCNS--VNAGWNH-DSTHVIRFPLNGY
CHLNSVQVLERLQQRGFEIAASCGGGVD-STQFSEYVLRREVKR----SHRGVMTSVIRIKQEPL-D
>KCTD1b_Stickleback
MARMPASGDAGRDQMSCPKRHA------------------------------------EGDNEGEDEDEIHEVQITGEEEDEESD------------------GDGVDLEWK
SRGYVLGSCSSAAQMRAVVMPSMDREEEG------------------------------EADPFSGGGSGGLESHLEGDLQRSERNKLSENTRLATRYAV
RIFREYLGEKSQSPDFETLDKETLCAVLRSFYAEARSKSGQLYSKSSLISIRSSLNRYLNEPPYCRTLDLTKDPELRSANLTLAAVIR
RLEEQGAGPVVQKQAITRSDLRKLYESSVFHADTPFGLLNKVWFETCMYFCTRGRENQRELEEDSFGLAVDEDGRNFVYFKALG
-----PYHKSRSAAWSKKRPDP---------DEDTLPRMYETRSEQCPYASFVRYVSKRNPLCRAFFQRPRDHCSATDVAWYENKAIGKN--
LLGTRMQMLSRAAKLSKTYTNHCIGAVSIATLNSIVGAAGSTTSTTLYVATETVNGHAQSNLPYLSRVADLKPAATTTVNTSSSTTT
TTTNKVIADEDPGAPH--AKRLCVRP-GTHAESL-------------------IERDQSEKGPAAA
TESPMHTHPV-----T--ARTQDSCVSSQQ----QVSVS---IVSHLGTAGIPLPAQ
LTKTNAPVHIDVGGHMYTSSLATLTKYPESRIGRLFEGTEPIVLDSLKQHYFIDRDGPMFRYILNFLRSSKLLIPDDFKEYSLLYEE
ASFFQLNSLQAALERWQTEQKCGKASL-------A---------------CECVVVHVAPELGERISVSADRALIKDVF-PEVRGVIFNS--QNTSGN
Q-ESSHVIRFPLNGHCHFNSVQVLERLQQRGFWITGSCGGGVD-SSQFSEYILRRESQE----SRHP--PTLIRIKQEFQ-N
>KCTD1b_Medaka_Hd-rR
MARMDGGRTAAQEEQRSPEG---GGGPEEVQRVQNSGDEEESD--------------------YDAEDWLWGSETT
VLDSSGSARQTRAVVMRSTERGPSEGNLEPHRCITP------------------------------------PGVDDKFRRGRLSENTRLATRYAVRIFREFLRDT
AQSPDFETLDKHALCARLRSFYVEARSKSGQVYSRSSLISIRSSLNRYLNEPPHSRTLDLTKDPEMRSANLVLAAVIRRLEEQGAG
PVVQKQAITRADLKKLYESSVFDTGTAYGLLNKVWFETCMYFCTRGRENQRELHEDSFGLAVDRNGRKFVFFKALGPNPRSPSH
GTRYVAWTRRHLDP----------QEASLPRMYETGTELCPYASFARFRSKRNPLCAAFFQRPRDQCSVSDVTWYENKAIGKN--LLGMR
MQMLSRAAKLSKTYTNHCIGAVSIATLNSIVGNRGYR------CAAETVKGHAKSMMQRVRRV-----SASLLLHPLSSSSSASRRRCQVG
QNGASPPAKKLRVHTQPDDVTSAKESHMTHMGEQVILQTCRQQNRKNDMGAEIRGGGDRA--
VPRGQRASCAGSRSTAPALFLPVLARCSSRLFASIYLSRNNPRPRPISYQRLSRDGGNGYHGLNASESEEAPASGQFCGRVRARVP
QTAADAGSGVQGARADVCGQRRASPRSIPEAERPLGEFLSYRSHAGSVSPLSNQGIPAPAQLTKSNAPVHIDVGGHMYTSSLATLT
KFPESRIGRLFDGTEPIVLDSLKQHYFIDRDGHMFRYILNFLRTSKLLIPEDFKDYSLLYEEARYFQLQPMLAELERWRQDQELGR
VSR-------P---------------CECLVVRVAPDLGERITLSGDKALIEDVF-PEIGDVMCNS--VNAGWNH-DSTHVIRFPLNGYCHLNSVQR---
----------SC--V
>KCTD1b_Medaka_HNI partial

--RENQR
ELHEDSFGWAVDRNGRKFVFFKALGPNPRSPNHGTRGAAWTRRHLDP----------QEASLPRMYETGTELCPYASFVRFRSKRNPLC
AAFFQRPRDHCSASDVTWYENKAIGKN--LLGMRMQMLSRAAKLSKTYTNHCIGAVSIATLNSIVGNRGYR------CAAETVKGHAK
SMMQRVRRV-----SASLLLHPLSSSFSASRRRCQAGQNGASPPAKKLRVNTQPDDVTSAKESHMTH--
---MGEQ----DSSKI---PS
TSPNSTGIPQVAQLTKSNAPVHIDVGGHMYTSSLATLTRYPDSRIARLFNGTEPVVLDSLKQHYFIDRDGPMFRYILNFLRTSKLLV
PEDFREYCLLYEEAVFFQLAPLLKELERWKAEQESPSGCR-------Q---------------CDCALVHVAPGLGEKVGVSACRSVIDEVI-PEV---
TMP--LSAGWTP-DCTHLSRFPLGSRCRLSSVQVSQTSIN-------ACPDLSD--PQVQAFLFQ--------------------------S
>KCTD1b_Pufferfish
MARMAGSGAAKEQVSWPQRT------------------------------------CTEELHEDQEDIQEVQISGEEEDEESDTGGV---------------------EPGRE
ARRASLEPGETRAVVMRSTDRGEEG--------------------------------------GEEGYPLEEEPQRPERNQLSENTRLATRYAVRIFREFLSETA
QSPHFETLDKDALCALLRSFYAEARSKSGQLYSKSSLISIRSSLNRYLNEPPYCRTLDLSKDPQLRSANLALAAVLRRLEEQGAAPV
VQKQAVTRSDLRRLYQSPVFDPDSPCGLLNKVWFETCMYFCTRGRENQRELREDSFGLAADQDGRRFVYLRAPG-----PSHRPRC
ALGTRRRPDA---------EEEPWPRMYETGTRLCPYASFLRYLAKRNRSCRAFFQRPRERSSAGDLTWYENKAIGKN--LLGTRMQML
SRSAKLSKTYTNHCIGAVSIATLNCLLGAADGR-----AATLEKVKGQKASPLRPERR--------------------------GPEDPSGPPRAKKRCVR
AG--
--------------------------------------SAAEGLGRGEERPGAAG------------------GGHAQEEPPG---PCPGQE
FSLLYEEACFFQHTPLQSQLQRWRRQRGCRSSWP-------------------------ECLLVHAAPELGEEVSVSAQRAVIQEVF-PEVGDLLRPA--
MNSCRNP-ESTHVSRFPLSSCCGLSWVQVLERLQRSGFCITCSCGGGVD-SSQFTEYFLQRE-------------------------
>KCTD1b_Shark partial

-------------RLSENTRLATRYAVKIFRDYLSERDHPPDFERLDKDALCRVLRSFYAEARSKSGQVYSKSSLISIRSSLNRYLNEPPYSRT
LDLTKDPELRSANLTLAAVIRRLEEQGAGPVIQKQAITR---
---------------------------CKAFFQRPRDHCNESDVTWYENKAIGKN--LLGTRMQMLSKAAKLSKTYTNHCIGAVSIATLSSIAGIQNHQ--
----

--GEIIGVKVETAMTPTLYEPVYSDDTCRP-------PSQKRLCVR--
------PSDCQVLERLQQKGFEIVGSCGGGVD-SSQFSEYVLRRQIRR----LCRV--PPVIRIKQEPL-D
>KCTD1_Lamprey partial

---IGRLFDGTEPIVLDSLKQHYFIDRD
GQMFRYILNFLRTSKLLIPDDFK---

>KCTD15_Human
MPHRKERPSGSSLHTHG---

-S---TGTAEGGNMSRL----SLTRS---PVSPLAAQGIPLPAQLTKSNAPVHIDV
GGHMYTSSLATLTKYPDSRISRLFNGTEPIVLDSLKQHYFIDRDGEIFRYVLSFLRTSKLLLPDDFKDFSLLYEEARYYQLQPMVRE
LERWQQEQEQRRRSR-------A---------------CDCLVVRVTPDLGERIALSGEKALIEEVF-PETGDVMCNS--VNAGWNQ-DPTHVIRFPL
NGYCRLNSVQVLERLFQRGFSVAASCGGGVD-SSQFSEYVLCREERR----PQPT--PTAVRIKQEPL-D
>KCTD15_Mouse
MPHRKERPSGSSLNAHG---

-S---SGTAEGGNMSRL----SLTRS---PVSPLAAQGIPLPAQLTKANAPVHIDV
GGHMYTSSLATLTKYPDSRISRLFNGTEPIVLDSLKQHYFIDRDGEIFRYILSFLRTSKLLLPDDFKDFNLLYEEARYYQLQPMVRE
LERWQQDQEQRRRSR-------A---------------CDCLVVRVTPDLGERIALSGEKALIEEVF-PETGDVMCNS--VNAGWNQ-DPTHVIRFP
LNGYCRLNSVQVLERLFQRGFSVAASCGGGVD-SSQFSEYVLCREERR----PQPT--PTAVRIKQEPL-D
>KCTD15_Dog
MPHRKERPSGSSLHAHG---

-S---AGTAEGGSMSRL----SLTRS---PVSPLAAQGIPLPAQLTKSNAPVHIDV
GGHMYTSSLATLTKYPDSRISRLFNGTEPIVLDSLKQHYFIDRDGEIFRYVLSFLRTSKLLLPDDFKDFSLLYEEARYYQLQPMVRE
LERWQQEQEQRRRSR-------A---------------CDCLVVRVTPDLGERIALSGEKALIEEVF-PETGDVMCNS--VNAGWNQ-DPTHVIRFPL
NGYCRLNSVQVLERLFQRGFSVAASCGGGVD-SSQISEYVLCREERR----PQPT--PTAVRIKQEPL-D
>KCTD15_Platypus partial

LLYEEAKYYQLQPMIKELERWKQEKEQRKHFQ-------P---------------CDCLVVRVTPDLGERIALSGEKALIEEIF-PETGDVMCNS--V
NAGWNQ-DPTHVIRFPLNGYCRLNSVQV---
>KCTD15_Chicken

--MSRL----SLTRS---PVSPLAAQGIPLPAQLTKSNAPVHIDVGGHMYTSSLATLTKY
PDSRISRLFNGTEPIVLDSLKQHYFIDRDGEIFRYILSFLRTSKLLLPEDFKDFNLLYEEAKYYQLQPMIKELERWKQEKEQRKHF
Q-------P---------------CDCLVVRVTPDLGERIALSGEKALIEEIF-PETGDVMCNS--VNAGWNQ-DPTHVIRFPLNGYCRLNSVQVLER
LFQKGFNMAASCGGGVD-SSQFSEYVLCREDRR----PQPT--PT-IRIKQEPL-D
>KCTD15_Frog

---MSRL----SLTRS---PVSPLAAQGIPLPAQLTKSNAPVHIDVGGHMYTSSLATLTKY
PDSRISRLFNGTEPIVLDSLKQHYFIDRDGEIFRYILSFLRTSKLLLPEDFKEFNLLYEEAKYYQLHPMVKELERWKQDKEHRKHF
Q-------P---------------CDCLVVRVTPDLGERIALSGEKALIEEIF-PETGDVMCNS--VNAGWNQ-DPTHVIRFPLNGYCRLNSVQVLER
MFQKGFHVAASCGGGVD-SSQFSEYVLCREDRR----MQPN---T-MRIKQEPL-D
>KCTD15_Zebrafish

---MSRL----SLTRS---PVSPLAAQGIPLPAQLTKANAPVHIDVGGHMYTSSLATLTKY
PDSRISRLFNGTEPIVLDSLKQHYFIDRDGEIFRYILSFLRTCKLLLPDDFKDFNLLYEEARYYQLSPMIKELERWKQEREQRRLAN
-------P---------------CDCLVVRVTPDLGERIALSGEKVLIEEIF-PETGDVMCNS--VNAGWNQ-DPTHVIRFPLNGYCRLNSVQVLERLF
QKGFNVAASCGGGVD-SSQFSEYVLCREDRR----SHNT--NTPIRIKQEPL-D
>KCTD15_Shark partial

---D
FSLLYEEAKYYQLQPMLKELERWKQDREFRKFSQ-------P---------------CDCVVVRVTPDLGERITLSGEKSLIEDIF-PETGDVMCNS
--VNAGWNQ-DPTHVIRFPLNGYCRLNSVQVLERLLQKGFSVVGSCGGGVD-SSQFSEYMLHRQDRR----LQLT--PTPVRIKQEPL-D
>Lancelet XP_002597685

--MTGIPRVASPCREKAPVHIDVGGHIYTSSLSTLAKYPESRLGRL
FNGSEPIVLDSLKQHYFIDRDGEMFGFILNYLRTGKLLLPDDFGTFDNLYEEARFFEIHPLVADLEKWREGKYYNKI------------------
-------CDVIVVHVSPELGERVCVSGERALIEELF-PEVSGAVCNS--AHAGWNH-DAHQVIRFPLNGFCSLNFVQVLQRMLQNGLRIR
ASCGGGVE-NSQFSEYVLFRERRV----------PPVKIKEEPIED
>SeaSquirt XP_002123395
MKR---
--SQDKKIHFADGPSIVGVASPAVPGSIHQNIQYPLKKKLRHAISAPP------------------------------------
--MPEHDGR
RITKQRTDKPPTSND--TRPPRTPPDIRYN---
---QPNIRT
TPNHEQPTLVGVPKAAVPSRYAAPVHIDVGGHIYTSSLETLTKFPDSKLGRLFSGAEPIVLDTLKQHYFIDRDGNMFRHILNFLRT
SNLALPDDFHELDQLLQEARFFELKLMVGQLEQFKESRRVSKTHK------------------------DCVVIRTVPDLGERICLGGNKSIIASIF-P
EVYDVITSA--DKCTWNQ-DPTHVIRFPINAFCQINSIKVLETFLSSGFSILAASGGGSESSSHFTEWVVARPRHNHDDVINTTSSTST
NKNSKLNSKT
>SeaUrchin XP_785600
MS---
---GVKRKMSSSLSPSP---
---VTTPRANS---------------------
--VTPPPTSTGIT--
--TPSTTQHQSSSQTQHSGVPRVAPNTRYTAPV
HIDVGGQIYTSTLETLTKFPESRLSKLFTGHIPIILDSLKQHYFIDRDGHLFRYILNYMRTARLLLPGDFTEMEALYEEARYYNLTP
MMEALEQMKSSEESSKTTQ-------EKREKTPAIKLESGCTGNECVIVHTSPDCVERISLSGDKNIIYELF-PEVGSIICNSSPTAAGWA
Q-DSNYVIRFPLNGFCKMNTVQVLQRLLQNNFGVIASSGGGIE-GSQFSEYVLSRKYGQ--------------------LT
>FruitFly CG10440
M--

---DRERERDVKALEPRDLSSTGRIYARSDIKISSSPTVSPTISNS---
--SSPTPTPPASSSVTPLGLPGAVAAAAAAVGGAS--
--SAGASSYLHGN
HKPITGIPCVAAASRYTAPVHIDVGGTIYTSSLETLTKYPESKLAKLFNGQIPIVLDSLKQHYFIDRDGGMFRHILNFMRNSRLLIAE
DFPDLELLLEEARYYEVEPMIKQLESMRKDRVRNGNYLVAPPTPPARHIKTSPRTSASPECNYEVVALHISPDLGERIMLSAERAL
LDELF-PEASQATQSSR-SGVSWNQGDWGQIIRFPLNGYCKLNSVQVLTRLLNAGFTIEASVGG-----QQFSEYLLARRVPM------------
----
>Mosquito AGAP007044-PA
MERDRDRDR--

---DRERDRAAERDVKPLEPRDLSAT-RLFTATQIKISTSPTTS
PTISNS---SSPTPTPPIP---------AVSPAVTGVG--
---SGYHHANHK
QITGIPCVAAASKYTAPVHIDVGGTIYTSSLETLTKYPDSRLAKLFNGCIPIVLDSLKQHYFIDRDGGMFRHILNFMRNSKLLVSED
FPDLELLLEEAKYFDIVPMIKQIELLKKERQRSGNGI--PPFGGGSRSKCK-GTVQTDTASHDVVALHISPDLGERILISAERAVLDEV
F-PETNQAILDAR-TGAAWNQFDGRQVIRFPLNGYCKLNSIQVLTRLLNAGFSVEASTGGGVE-TQQFSEYLLIRKSAM----------------
------
